# Causal Discovery with Generalized Linear Models through Peeling Algorithms

**Published:** 2024

**Authors:** Minjie Wang, Xiaotong Shen, Wei Pan

**Affiliations:** Department of Mathematics and Statistics, Binghamton University, State University of New York, Binghamton, NY 13902, USA; School of Statistics, University of Minnesota, Minneapol*is*, MN 55455, USA; Division of Biostatistics, University of Minnesota, Minneapolis, MN 55455, USA

**Keywords:** Generalized linear models, large directed acyclic graphs, hierarchy, nonconvex minimization, mixed graphical models

## Abstract

This article presents a novel method for causal discovery with generalized structural equation models suited for analyzing diverse types of outcomes, including discrete, continuous, and mixed data. Causal discovery often faces challenges due to unmeasured confounders that hinder the identification of causal relationships. The proposed approach addresses this issue by developing two peeling algorithms (bottom-up and top-down) to ascertain causal relationships and valid instruments. This approach first reconstructs a super-graph to represent ancestral relationships between variables, using a peeling algorithm based on nodewise GLM regressions that exploit relationships between primary and instrumental variables. Then, it estimates parent-child effects from the ancestral relationships using another peeling algorithm while deconfounding a child's model with information borrowed from its parents' models. The article offers a theoretical analysis of the proposed approach, establishing conditions for model identifiability and providing statistical guarantees for accurately discovering parent-child relationships via the peeling algorithms. Furthermore, the article presents numerical experiments showcasing the effectiveness of our approach in comparison to state-of-the-art structure learning methods without confounders. Lastly, it demonstrates an application to Alzheimer's disease (AD), highlighting the method's utility in constructing gene-to-gene and gene-to-disease regulatory networks involving Single Nucleotide Polymorphisms (SNPs) for healthy and AD subjects.

## 1. Introduction

Discovering causal relationships among variables is crucial for scientific inquiries in various fields, including genetics, artificial intelligence, and social science. For instance, in genetics, biologists aim to uncover gene-gene regulatory relationships, while neuroscientists focus on causal influences between different regions of interest in a patienťs brain. However, unmeasured confounders can arise when randomized experiments are unethical or infeasible, which distort the discovery process and obscure the relationship between exposures and the outcome variable, leading to false discoveries. Consider our motivating case study on inferring regulatory networks from the Alzheimer's disease gene expression data. We study a subset of genes while other genes are not included and removed by the prescreening procedure, which introduces unmeasured confounders. Meanwhile, in neuroscience, existing technologies can only record from a small subset of neurons in the brain at once, also leading to confounders. This article proposes a novel approach to causal discovery using instrumental variables to correct confounding effects, yielding accurate causal discovery, particularly for discrete outcomes such as binary, count-valued, and multinomial.

Causal discovery necessitates estimating parent-child relationships, or equivalently, the graph structure of a directed acyclic graph (DAG). DAGs are an effective tool for describing directional effects in causal discovery, but reconstructing a DAG structure poses computational challenges due to the acyclicity constraint. Two popular approaches for reconstructing a Gaussian DAG structure without confounders are the sequential conditional independence tests, such as the PC algorithm (Spirtes et al. 2000), and the likelihood-based methods subject to the acyclicity constraint (Zheng et al. 2018; Yuan et al. 2019). Recently, Li et al. (2023) proposed a linear causal discovery method without confounders through interventions. Going beyond, for non-Gaussian outcomes, Zheng et al. (2020) extended the algebraic characterization of DAGs by Zheng et al. (2018) to nonparametric and semiparametric models including GLMs; Shi et al. (2023) proposed a new hypothesis testing method for nonlinear DAG models. However, despite recent work, causal discovery for discrete outcome data, particularly in the presence of confounders, has received limited attention, and unique challenges arise when handling such data. One challenge is the non-identifiability of the logistic DAG model, even without confounders (Park and Raskutti 2018). Moreover, in the presence of confounders, unmeasured confounders can distort causal effect estimation, making structural equation models non-identifiable. Another challenge is the typically intractable form of the marginal likelihood, despite an interpretable conditional likelihood and data-specific noise or variance. It also remains unclear how to separate confounders from causal effects in the discovery process. Some recent proposals focus on simple situations, such as the two-stage least squares (Theil 1992), an instrumental variable (IV) regression of continuous outcomes given a known causal order, the two-stage predictor substitution (2SPS, Cai et al. (2011)) and two-stage residual inclusion (2SRI, Hausman (1978); Terza et al. (2008)) for general nonlinear outcomes, including discrete outcome data. However, none of these approaches apply to causal discovery with an unknown causal order and multiple primary variables.

This article proposes a new approach called GAMPI (Generalized Linear Models with Peeling and Instruments) for causal discovery of multiple primary variables from various data types. GAMPI involves a two-step process. First, we propose a fidelity model as a simple surrogate for the original intractable marginal model, which retains intervention characteristics. Then, we design a bottom-up peeling algorithm to reconstruct the super-graph consisting of ancestral relationships while identifying valid instrumental variables (IVs) for each primary variable by exploiting the connections between the primary and instrumental variables to determine the causal order. For each primary variable, a constrained generalized linear model (GLM, Nelder and Wedderburn (1972)) subject to the truncated $\ell_{1}$-penalty constraint (TLP, Shen et al. (2012)) is fit on the instrumental variables to identify nonzero-coefficient IVs, followed by a difference-of-convex (DC) algorithm to solve the corresponding nonconvex minimization. In the second step, given the identified super-graph, we develop a top-down peeling algorithm to estimate the direct causal effects of each primary variable while identifying its parents from ancestors. In this peeling process, we propose a novel deconfounding approach using the estimated confounders from the parents' equation models to correct the confounding effects of a child's equation model. This approach fits a TLP-constrained GLM to each primary variable on its ancestors and residuals from its ancestors' models to identify parents and estimate the direct causal effect of each parent-child relationship.

This article contributes to causal discovery. It introduces a comprehensive approach capable of handling diverse data types with unobserved confounders, ensuring the identification of parent-child relationships through valid instruments for each primary variable. This involves generalized linear models, addressing both discrete and mixed (continuous and discrete) outcomes while considering confounders beyond Gaussian data without confounders by Li et al. (2023). In particular,

1. It establishes the identifiability of generalized structural equation models with confounders and instruments, valid and invalid. This result does not require additional assumptions for each primary variable with a nonlinear link, unlike the Gaussian case which requires valid instrumental variables to be the majority of the instrumental variables (Kang et al. 2016; Windmeijer et al. 2019).
2. It introduces a fidelity model to handle intractable likelihoods and eliminate the confounding effects for identifying ancestral relationships.
3. It designs a projection-based difference-convex (DC) algorithm to solve nonconvex minimization for a constrained generalized linear model regression. This algorithm delivers a global minimizer with high probability and a computational complexity of $q^{2}\;\text{max}(q,n)\text{log}\;K^{0}$, where q,n, and $K^{0}$ are the numbers of regressors, the sample size, and the nonzero regression coefficients.
4. It develops bottom-up and top-down peeling algorithms to estimate the causal order and the causal effects for primary variables. These algorithms require solving at most p generalized linear model regressions subject to the truncated $\ell_{1}$-penalty constraint, where p is the number of primary variables.
5. It shows that GAMPI yields the correct discovery of all parent-child relationships, providing statistical guarantees for GAMPI.
6. It demonstrates the superior performance of GAMPI for logistic and Poisson models over state-of-the-art methods, NOTEARS (Zheng et al. 2018, 2020) and a faster version of NOTEARS, called DAGMA (Bello et al. 2022), especially in the presence of confounders. It suggests that GAMPI corrects the confounding effects without imposing additional noise variance structures to reconstruct a causal graph.

The rest of the article is structured as follows. Section 2 introduces generalized structural equation models with confounders and instruments. Section 3 introduces the fidelity model and three algorithms, one DC and two peeling algorithms, for identifying the ancestral and then parent-child relationships. Section 4 investigates the statistical properties of the proposed approach. Section 5 performs simulation studies, followed by Section 6 with an application to Alzheimer's disease to reconstruct a gene-to-gene and gene-to-disease regulatory network. Section 7 concludes the article. The Appendix contains illustrative examples, technical proofs, and additional simulations.

## 2. Generalized Structural Mean Models

### 2.1 Directed Acyclic Graphs, Confounders, and Interventions

Given a vector of primary variables of interest $Y={\left(Y_{1},\ldots ,Y_{p}\right)}^{⊤}$, the joint probability of a generalized structural equation model (SEM, Pearl (2000)) with confounders $h={\left(h_{1},\ldots ,h_{p}\right)}^{⊤}$ and instrumental variables $X={\left(X_{1},\ldots ,X_{q}\right)}^{⊤}$ can be factorized as:

(1) $$P(Y|X,h)=\prod_{j=1}^{p}P\left(Y_{j}|Y_{\text{pa}(j)},X,h_{j}\right),$$

where $P\left(Y_{j}|Y_{\text{pa}(j)},X,h_{j}\right)$ denotes the conditional probability of $Y_{j}$ given $Y_{\text{pa}(j)},X,h_{j}$, which follows an exponential family distribution. Here, unmeasured confounders refer to variables that are not included in the model, but nonetheless affect the primary variables of interest. Confounders $h_{j}$ and $h_{j^{\prime }}$ can be correlated among equations for $j\neq j^{\prime }$. An instrumental variable (IV) is a variable that affects the primary variables of interest, but not vice versa, i.e., the primary variable should not have an impact on the IVs. In practice, one may choose candidate IV sets based on scientific knowledge, as in Section 6. Note that (1) characterizes a DAG under the acyclicity constraint. Moreover, the conditional distribution of $Y_{j}$ is characterized by a generalized linear model:

(2) $$\psi_{j}\left(E\left[Y_{j}|Y_{\text{pa}(j)},X,h_{j}\right]\right)=U_{\text{pa}(j),j}^{⊤}Y_{\text{pa}(j)}+W_{\text{in}(j),j}^{⊤}X_{\text{in}(j)}+h_{j},\;j=1,\ldots ,p,$$

where $\psi_{j}(\cdot )$ is a monotone link function for a GLM chosen to be appropriate for the data type of $Y_{j}$ (cf. Table 1), $\mathrm{pa}(j)\equiv \left\{k:u_{kj}\neq 0\right\}=\left\{k:Y_{k}\to Y_{j}\right\}$ denotes a set of parent variables of $Y_{j}$, defined by the parent-child relationship $Y_{k}\to Y_{j},\text{in}(j)\equiv \left\{l:w_{lj}\neq 0\right\}=\left\{l:X_{l}\to Y_{j}\right\}$ denotes a set of the associated instrumental variables of $Y_{j}$, defined by an intervention from $X_{l}$ to $Y_{j}:X_{l}\to Y_{j}$, and $Y_{A}={\left(Y_{k_{1}},\cdots ,Y_{k_{M}}\right)}^{⊤},k_{m}\in A$, is a sub-vector of Y indexed by A. Here, $U=\left(u_{kj}\right)$ and $W=\left(w_{lj}\right)$ are the p×p adjacency and q×p intervention matrices, and $U_{\text{pa}(j),j}={\left(u_{kj}\right)}_{k\in \text{pa}(j)}$ and $W_{\text{in}(j),j}={\left(w_{lj}\right)}_{l\in \text{in}(j)}$ are sub-vectors of the jth column vector of $U,U_{•j}=\left(u_{kj}\right)$ and the jth column vector of ${W,W_{•j}=\left(w_{lj}\right).}^{⊤}$ denotes the transpose. Note that the p structural equations can possess different $\psi_{j}\text{s}$, depending on the data type of $Y_{j}$, reminiscent of the mixed graphical models framework (Yang et al. 2014). We refer the reader to Section 6 for an illustrative example.

The adjacency matrix U specifies a directed acyclic graph (DAG) with each primary variable as a node, and its non-zero elements represent directed edges between nodes. To prevent directed cycles, U is subject to the acyclicity constraint (Zheng et al. 2018; Yuan et al. 2019).

### 2.2 Identifiability

Model (2) encodes a DAG model describing multiple parent-child relationships, which, however, is generally not identifiable in the presence of unmeasured confounders h. Note that (2) may not be identifiable even in the absence of confounders h, for instance, a logistic model without instrumental variables and confounders (Park and Raskutti 2018). However, as suggested by Proposition 1, with suitable instruments, (2) is identifiable.

To proceed, we first categorize instrumental variables (IVs) into valid IVs and non-valid IVs (covariates). A valid instrument $X_{l}$ for primary variable $Y_{j}$ satisfies:

- Relevance: it intervenes on $Y_{j}$;
- Exclusion: it does not intervene on other primary variables.

Otherwise, it is a non-valid IV that intervenes on none or multiple primary variables. Let ${\text{in}}_{*}(j)$ denote a set of valid IV of $Y_{j}$. Next, we make some assumptions on instruments for model (2).

**Assumption 1** *Assume that for* j=1,…,p, *model* (2) *satisfies*:

*(A) (Local faithfulness)* $\text{Cov}\left(Y_{j},X_{l}|X_{\left\{1,\cdots ,q\}\setminus \{l\right\}}\right)\neq 0$ *when* $X_{l}$ *intervenes on an immediate parent of* $Y_{j}$, *where Cov denotes the covariance*.

*(B) (Instrumental sufficiency) Each primary variable is intervened by at least one valid IV. If* $\psi_{j}$ *is linear, then the number of valid IVs for* $Y_{j}$ *is required to exceed 50% of its total number of IVs, known as the majority rule. Otherwise, the majority rule is not required for a specific nonlinear* $\psi_{j}$.

*(C) (Validity) Confounders* $h={\left(h_{1},\ldots ,h_{p}\right)}^{⊤}$ *and valid instruments* $X_{in_{*}}={\left(X_{in_{*}(1)},\ldots ,X_{in_{*}(p)}\right)}^{⊤}$ *are independent. That is, for each pair of* $\left\{\left(l,j),l\in \cup_{j=1}^{p}in_{*}(j\right)\right\},X_{l}$ *and* $h_{j}$ *are independent*.

Assumption 1(A) guarantees that other interventions donť offset an intervention from $X_{l}$ to $Y_{j}$, while Assumption 1(B) ensures that each primary variable has at least one valid IV. Both are necessary for the identifiability of a Gaussian structural model (Li et al. 2023). The second condition in Assumption 1(B) requires the majority rule for a linear link, which amounts to the so-called majority requirement for Gaussian data (Kang et al. 2016; Windmeijer et al. 2019). However, such a majority condition is not required for a nonlinear link function. We provide an illustrative example of the majority rule in Appendix A.2. Note Assumption 1(B) considers a GLM with the canonical link as well as the non-canonical link, defined by model (2). Assumption 1(C) is also required by the two-stage least squares and residual inclusion methods for the IVs (Lousdal 2018; Terza et al. 2008), known as the instrumental validity assumption. Further, the instrumental variables X and confounders are independent by parameterization, i.e., projecting $h_{j}$ onto the space spanned by the non-valid IVs. Given Assumption 1, Proposition 1 suggests the identifiability of model (2).

**Proposition 1 (Identifiability)** *Under Assumption 1, model* (2) *is identifiable for model parameters* (U,W).

Proposition 1 suggests that a nonlinear link function permits the identification of the parents of a primary variable, which is unlike the linear link for Gaussian data. This new result highlights the importance of a link function concerning the model identifiability of causal effects.

## 3. Method

This section estimates (U,W) to identify parent-child relationships and the corresponding interventions in (2). Due to the model identifiability issue of (2), direct estimation of U is impossible without the help of instrumental variables X. To estimate parent sets pa(j),j=1,…,p, and thus U, we first need to determine the causal order, which amounts to determining ancestral relationships, including all parent-child relationships. Here, $Y_{k}$ is an ancestor of $Y_{j}$, or $Y_{j}$ is an offspring of $Y_{k}$, denoted by $Y_{k}⇝Y_{j}$, if there exists a directed pathway $Y_{k}\to Y_{k_{1}}\to \ldots \to Y_{k_{m}}\to Y_{j}$, where $Y_{k}\to Y_{k_{1}}$ is a parent-child relationship defined by U. Subsequently, an (j) denotes a set of ancestors of $Y_{j}$. Once an (j) is identified, we then pinpoint pa(j) via a deconfounding approach in Section 3.3.

### 3.1 Fidelity Models

This subsection introduces a working model termed as the “fidelity model”, to identify all ancestral relationships. The term “fidelity model” is named as it yields the same support as the marginal distribution of the original model. Towards this end, we exploit the connections between a primary variable and the associated instrumental variables, described by the conditional distribution of $Y_{j}$ given X from (2), $P\left(Y_{j}|X\right)$, to identify the causal orders among primary variables. However, $P\left(Y_{j}|X\right)$ is generally intractable even given an analytic expression of $\left.P\left(Y_{j}|Y_{\text{pa}(\text{j}}\right),X,h_{j}\right)$ in (2). To overcome this difficulty, we introduce the fidelity model that is also a GLM:

(3) $$\psi_{j}\left(E\left(Y_{j}|X\right)\right)=V_{•j}^{⊤}X,\;j=1,\cdots ,p,$$

where $\psi_{j}$ is set to be the same as in (2). Here, $V_{•j}=\left(V_{1j},\ldots ,V_{qj}\right)$ is the jth column vector of a q×p matrix $V=\left(V_{•1},\ldots ,V_{•p}\right)$. This model (3) is motivated by the observation that the conditional distribution of $Y_{j}$ given X, denoted by $P^{*}\left(Y_{j}|X\right)$ and defined by (3), satisfies $\frac{\partial P^{*}\left(Y_{j}|X\right)}{\partial X_{m}}\neq 0$ if and only if $\frac{\partial P\left(Y_{j}|X\right)}{\partial X_{m}}\neq 0$ based on (2) due to the properties of GLMs, as shown in Proposition 2, where $\frac{\partial }{\partial X_{m}}$ denotes the partial derivative with respect to $X_{m}$.

The conditional distribution $P^{*}\left(Y_{j}|X\right)$ defined by the fidelity model (3) not only provides a simple form to work with, but also has the same support as the intractable marginal distribution $P\left(Y_{j}|X\right)$ under (2), although with different intervention magnitudes. In particular, a nonzero l-th element of $V_{•j}$ indicates that $Y_{k}$ is an ancestor of $Y_{j}$ if $X_{l}$ is a valid IV of $Y_{k}$. This property permits the identification of the super-graph characterizing all the ancestral relationships, as shown in Proposition 3.

We define the index set of $X_{1},\cdots ,X_{q}$ with nonzero coefficients in the fidelity model (3) and in the true model $P\left(Y_{j}|X\right)$ marginalized from (2) as $S_{j}=\left\{m:V_{mj}\neq 0\right\}$ and ${\tilde{S}}_{j}=\left\{m:\frac{\partial P\left(Y_{j}|X\right)}{\partial X_{m}}\neq 0\right\}$, respectively, for j=1,…,p. The following Proposition 2 establishes the connections between the fidelity model and the marginal distribution of the true model $P\left(Y_{j}|X\right)$.

**Proposition 2 (Support preservation)** *Assume that Assumption 1 is satisfied and the link function* $\psi_{j}s$ *in* (2) *are differentiable. Then*, $P^{*}\left(Y_{j}|X\right)$ *defined by the fidelity model* (3) *has the same support as* $P\left(Y_{j}|X\right)$ *under the full model* (2), *that is*, $S_{j}={\tilde{S}}_{j},j=1,\ldots ,p$.

Proposition 2 suggests that the fidelity model (3) retains the intervention structure of $P\left(Y_{j}|X\right)$ in the original model concerning the presence or absence of a specific intervention. It is worth mentioning that the fidelity model (3) eliminates the confounding effects when identifying the support of $P\left(Y_{j}|X\right)$ and hence the ancestral relationships or the causal order among $Y_{1},\ldots ,Y_{p}$. This property is due to Assumption 1(C) that X are independent of confounders h. Consequently, the confounders are marginalized for X and thus have no impact on the support of $P\left(Y_{j}|X\right)$.

**Figure 1:**
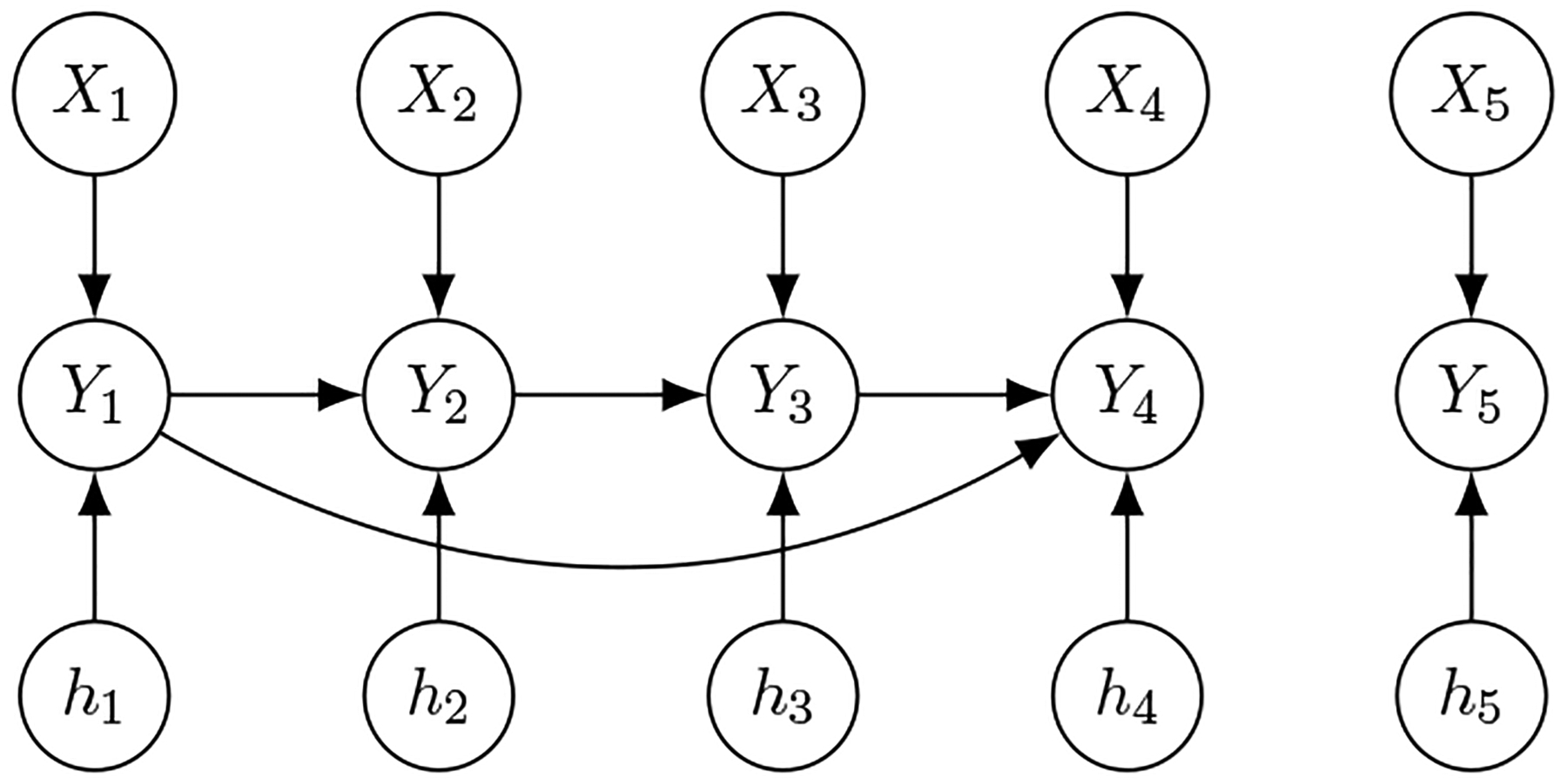
Example DAG defined by model (4).

To illustrate the fidelity model and Proposition 2, we here include a motivating example. Consider a generalized structural equation model for binary outcomes with p=q=5:

(4) $$\begin{array}{ll} \psi \left(E\left[Y_{1}|X_{1},h_{1}\right]\right)=2X_{1}+h_{1}, & \psi \left(E\left[Y_{2}|Y_{1},X_{2},h_{2}\right]\right)=1.5Y_{1}+2X_{2}+h_{2},\\ \psi \left(E\left[Y_{3}|Y_{2},X_{3},h_{3}\right]\right)=1.5Y_{2}+2X_{3}+h_{3}, & \psi \left(E\left[Y_{4}|Y_{3},Y_{1},X_{4},h_{4}\right]\right)=-1.5Y_{1}+1.5Y_{3}+2X_{4}+h_{4},\\ \psi \left(E\left[Y_{5}|X_{5},h_{5}\right]\right)=2X_{5}+h_{5}, & \; \end{array}$$

where $\psi_{1}=\cdots =\psi_{5}=\psi$ is the logit link function. Here, (4) defines a DAG shown in Figure 1. Note that marginalizing each equation in (4) over $Y_{-j}$ does not lead to closed-form expressions for $Y_{j}|X$.

The proposed fidelity model that has the same support as the marginal model of (4) is:

$$\begin{array}{ll} \psi \left(E\left[Y_{1}|X_{1}\right]\right)=V_{11}X_{1}, & \psi \left(E\left[Y_{3}|X_{1},X_{2},X_{3}\right]\right)=V_{13}X_{1}+V_{23}X_{2}+V_{33}X_{3},\\ \psi \left(E\left[Y_{2}|X_{1},X_{2}\right]\right)=V_{12}X_{1}+V_{22}X_{2}, & \psi \left(E\left[Y_{4}|X_{1},X_{2},X_{3},X_{4}\right]\right)=V_{14}X_{1}+V_{24}X_{2}+V_{34}X_{3}+V_{44}X_{4},\\ \psi \left(E\left[Y_{5}|X_{5}\right]\right)=V_{55}X_{5}. & \; \end{array}$$

Note that the fidelity model has the same support as the true marginal model and in the next section, we show that ancestral relationships can be identified via V.

### 3.2 Identifying Ancestral Relationships

This subsection proposes a novel structure learning method to identify the ancestral relationships. To start with, we introduce a proposition demonstrating the connections between the primary variables and instrumental variables via V in the fidelity model.

**Proposition 3 (Identification of ancestral relationships via** V**)** *Assume that Assumption 1 is met. Then*,

*(a) For a valid instrument* $X_{l}$, *if* $V_{lj}\neq 0$, *then* $X_{l}$ *intervenes on* $Y_{j}$ *or an ancestor of* $Y_{j}$.

*(b)* $Y_{j}$ *is a leaf variable with no children if and only if there exists a valid instrument* $X_{l}$ *such that* $V_{lj}\neq 0$ *and* ${\left\|V_{l•}\right\|}_{0}=1$.

*(c) If* $V_{lj}\neq 0$ *and* $X_{l}$ *is a valid instrument for* $Y_{k}$, *then* $Y_{k}$ *is an ancestor of* $Y_{j}$, *that is*, $Y_{k}⇝Y_{j}$.

Proposition 3 suggests that the topological order of a DAG can be reconstructed by recursively identifying and removing (“peeling off”) leaf variables in the graph, as long as the non-zero elements of V are obtained. We define $Y_{j}$ as a leaf variable if it has no children. Next, we first introduce a nodewise constrained GLM-based approach to estimate the non-zero elements of V and then propose a peeling algorithm to identify the ancestral relationships from V based on Proposition 3.

#### 3.2.1 Nodewise constrained GLM Regressions

This subsection proposes nodewise constrained GLM regressions subject to the $\ell_{0}$-constraint based on the fidelity model to estimate nonzero elements of V in (3).

Consider the data matrix $\left(X_{n\times q},Y_{n\times p}\right)$ where $X_{i•}$ and $Y_{i•}$ refer to the ith row of X and Y. Given independent observations ${\left(X_{i•},Y_{i•}\right)}_{i=1}^{n}$, let $ℒ\left(V_{•j}\right)=n^{-1}\sum_{i=1}^{n}\ell \left(Y_{ij},V_{•j}^{⊤}X_{i•}\right)$ denote the negative log-likelihood for a GLM, where $\ell \left(Y_{ij},V_{•j}^{⊤}X_{i•}\right)$ is the negative log-likelihood for $Y_{ij}$ given $X_{i•};$ refer to Table 1 and (12) for details. For example, $\ell \left(Y_{ij},V_{•j}^{⊤}X_{i_{•}}\right)=\left(-Y_{ij}\left(V_{•j}^{⊤}X_{i_{•}}\right)+\text{log}\left(1+\text{exp}\left(V_{•j}^{⊤}X_{i_{•}}\right)\right)\right)$ for a logistic model.

For j=1,…,p, the nodewise constrained GLM regression solves the following minimization with a nonconvex constraint:

(5) $${\hat{V}}_{•j}=\text{arg}\;\underset{V_{•j}}{\text{min}\;}ℒ\left(V_{•j}\right)\;\text{subject}\;\text{to}\;\sum_{l=1}^{q}I\left(V_{lj}\neq 0\right)\leq K_{j},$$

where $1\leq K_{j}\leq q$ is an integer-valued tuning parameter. Note that $K_{j}\geq 1$ ensures that each variable $Y_{j}$ receives at least one valid IV, as required by Assumption 1(B). Here, we impose the $\ell_{0}$-constraint to obtain the exact number of non-zeros as opposed to the $\ell_{1}$ version. Note many other penalty functions in the literature induce sparsity such as the $\ell_{1}$-penalty (Tibshirani 1996) and the minimax concave penalty (MCP, Zhang (2010)). However, these penalty functions do not yield the exact number of non-zero coefficients to ensure that each variable $Y_{j}$ receives at least one valid IV, i.e., $K_{j}\geq 1$, required by Assumption 1(B).

To solve the nonconvex minimization (5), we propose a projection-based difference-convex (DC) algorithm for efficient computation. The constrained problem is equivalent to solving a penalized version of (5) by adding a penalty term to the objective function. Specifically, we minimize $ℒ\left(V_{•j}\right)+\lambda_{j}\sum_{l=1}^{q}I\left(V_{lj}\neq 0\right)$, where $\lambda_{j}>0$ is a computational parameter corresponding to the constrained parameter $K_{j}$ in (5). Next, we replace the $\ell_{0}$-indicator function with its computational surrogate, the truncated $\ell_{1}$-function (TLP) denoted by $J_{\tau }(\cdot )$, where $J_{\tau }(z)=\text{min}(|z|/\tau ,1)$, as suggested by Shen et al. (2012). We decompose $J_{\tau }$ into a difference of two convex functions: $J_{\tau }(z)=S_{1}(z)-S_{2}(z)\equiv |z|/\tau -\text{max}(|z|/\tau -1,0)$, to construct an upper approximation of the cost function iteratively. At the t-th iteration, we approximate $J_{\tau }$ by $S_{1}(z)-S_{2}\left(z^{\left[t-1\right]}\right)-\nabla S_{2}{\left(z^{\left[t-1\right]}\right)}^{⊤}(z-\left.z^{\left[t-1\right]}\right)=\frac{\left|z\right|}{\tau }\cdot I\left(\left|z^{\left[t-1\right]}\right|\leq \tau \right)+1-I\left(\left|z^{\left[t-1\right]}\right|\leq \tau \right)$ based on the DC decomposition. Then, we solve the unconstrained minimization problem:

(6) $${\tilde{V}}_{•j}^{\left[t\right]}=\text{arg}\;\underset{V_{lj}}{\text{min}\;}ℒ\left(V_{•j}\right)+\gamma_{j}\tau_{j}\sum_{l=1}^{q}I\left(\left|{\tilde{V}}_{lj}^{\left[t-1\right]}\right|\leq \tau_{j}\right)\left|V_{lj}\right|,$$

where $\gamma_{j}=\lambda_{j}/\tau_{j}^{2}$. The DC algorithm iterates until a stopping criterion is met. In particular, let $\overset{‾}{f}(\cdot )$ denote the objective function in (6). The DC algorithm terminates at iteration T when $\left|\overset{‾}{f}\left({\tilde{V}}_{•j}^{\left[T\right]}\right)-\overset{‾}{f}\left({\tilde{V}}_{•j}^{\left[T-1\right]}\right)\right|\leq \epsilon_{\text{tol}}$, where $\epsilon_{\text{tol}}$ is the tolerance level. Finally, the estimated solution ${\hat{V}}_{•j}$ is computed by projecting the penalized solution onto the constraint set $\left\{{\left\|V_{•j}\right\|}_{0}\leq K_{j}\right\}$. In this paper, $\|\cdot {\|}_{q}$ denotes the $\ell_{q}$-norm of a vector and $\|x{\|}_{0}=\sum_{j}I\left(x_{j}\neq 0\right)$. In practice, we use either 5-fold cross-validation or the extended Bayesian information criterion (EBIC, Chen and Chen (2008)) to choose $\left(\tau_{j},K_{j}\right)$. We recommend EBIC due to its computational efficiency and strong empirical performance. Algorithm 1 summarizes the DC algorithm for solving nonconvex minimization (5).

**Algorithm 1:** DC algorithm for nonconvex minimization (5)

1. **(Initialization)** Specify tuning parameters $\left(\tau_{j},K_{j}\right)$. Initialize ${\left\|{\tilde{V}}_{•j}^{\left[0\right]}\right\|}_{0}\leq K_{j}$, and choose a sequence of $\gamma_{j}$ so that $\left|C_{j}\right|\geq K_{j}$ in Step 4.
2. **(Relaxation)** Compute the penalized solution ${\tilde{V}}_{•j}^{\left[t\right]}$ of (6).
3. **(Termination)** Repeat Step 2 until a termination criterion is met. Compute $\tilde{V}:{\tilde{V}}_{•j}={\text{arg}\;\text{min}}_{V_{•j}}ℒ\left(V_{•j}\right)$ with $V_{•j}\in {\left({\tilde{V}}_{•j}^{\left[t\right]}\right)}_{t=1}^{T}$, where T is the iteration index at termination.
4. **(Projection)** Let $C_{j}=\left\{l:\left|{\tilde{V}}_{lj}\right|>{\left|{\tilde{V}}_{•j}\right|}_{\left(K_{j}+1\right)}\right\}$, where ${\left|{\tilde{V}}_{•}\right|}_{\left(K_{j}+1\right)}$ is the $\left(K_{j}+1\right)$th largest absolute value of the coefficients. Set ${\hat{V}}_{•j}={\text{arg}\;\text{min}}_{V_{•j}}ℒ\left(V_{•j}\right)$ subject to $V_{lj}=0$ for $l\notin C_{j}$.

Remark: Computing $\hat{V}=\left({\hat{V}}_{•1},\ldots ,{\hat{V}}_{•p}\right)$ amounts to applying Algorithm 1 p times. The computational complexity of Algorithm 1 to solve one $\ell_{0}$-constrained regression in (5) is the number of DC iterations multiplied by that of solving a weighted Lasso regression for a GLM, which is $q^{2}\text{max}(q,n)\text{log}\;K_{j}^{0}$ (Efron et al. 2004).

#### 3.2.2 Identifying Ancestral Relationships via Peeling

Given the nonzero elements of $\hat{V}$ obtained by Algorithm 1, we now introduce a bottom-up peeling algorithm to estimate ancestral relationships through the nonzero elements of $\hat{V}$ using Proposition 3. This algorithm constructs a hierarchy of different layers of primary variables, defined by the causal ordering of the variables. The algorithm begins with leaf variables at the bottom, and proceeds by recursively identifying and peeling off one leaf layer of primary variables along with the associated instrumental variables in the graph. Specifically, at iteration h, based on Proposition 3 (b), the algorithm first identifies all leaf nodes $Y_{k}$ in the subgraph with ${\hat{V}}_{lk}^{\left[h\right]}\neq 0$ and instrumental variables $X_{l}$ such that ${\left\|{\hat{V}}_{l•}^{\left[h\right]}\right\|}_{0}=1$. In practice, the condition ${\left\|{\hat{V}}_{l•}^{\left[h\right]}\right\|}_{0}=1$ may not hold due to estimation error. To address this issue, we identify the rows of ${\hat{V}}^{\left[h\right]}$ with the smallest positive $\ell_{0}$-norm, that is, ${\{l^{*}:l^{*}=\text{arg}\;{\text{min}}_{l=1}^{q}\left\|{\hat{V}}_{l•}^{\left[h\right]}\right\|}_{0}$, s.t ${\left\|{\hat{V}}_{l•}^{\left[h\right]}\right\|}_{0}\geq 1\}$, followed by identifying the largest absolute value element index $k^{\text{*}}={\text{arg}\;\text{max}}_{k=1}^{p}\left|{\hat{V}}_{l^{\text{*}}k}^{\left[h\right]}\right|$ of the $l^{*}$th row for each $l^{*}$. By Proposition 3(b), $X_{l^{*}}\to Y_{k^{*}}$. Moreover, the algorithm identifies the ancestral relationship $Y_{k^{*}}⇝Y_{j}$ if an instrument $X_{l^{*}}$ for the primary variable $Y_{k^{*}}$ also satisfies ${\hat{V}}_{l^{*}j}\neq 0$ for a previously peeled off $Y_{j}$, according to Proposition 3 (c). The algorithm continues by peeling off all the current leaf-instrument $X_{l}\to Y_{k}$ pairs (i.e., removing the lth row and kth column from the current ${\hat{V}}^{\left[h\right]}$) to focus on the subgraph. This peeling process repeats until all primary variables are removed. The super-graph $\hat{𝒮}$ contains all the ancestral relationships identified during this process. Lastly, the algorithm computes the causal ordering from the super-graph $\hat{𝒮}$, which is defined as a linear ordering of the nodes where each node appears before all nodes to which it has edges.

**Algorithm 2:** Peeling algorithm for identifying all ancestral relationships

1. **(Initialization)** ${\hat{V}}^{\left[1\right]}=\hat{V}$ and $\hat{𝒮}=\emptyset$.
Begin iteration h=1,⋯: at iteration h,
2. **(Leaf-IV pairs)**
(a) Identify rows of ${\hat{V}}^{\left[h\right]}$ with the smallest positive $\ell_{0}$-norm. Store indices of all IVs associated with leaf variables in $A^{\left[h\right]}=\left\{l^{*}:l^{*}=\text{arg}\;\text{min}{\left\|{\hat{V}}_{l•}^{\left[h\right]}\right\|}_{0}\right\}$.
(b) Identify the largest absolute value element index of the $l^{*}$th row for each $l^{*}\in A^{\left[h\right]}:B_{l^{*}}^{\left[h\right]}=\left\{k^{*}:k^{*}=\text{arg}\;\text{max}\left|{\hat{V}}_{l^{*}k}^{\left[h\right]}\right|\right\}$. Identify all leaf-IV pairs: $X_{l^{*}}\to Y_{k^{*}}$. Let $B^{\left[h\right]}=\underset{l^{*}}{⋃}B_{l^{*}}^{\left[h\right]}$.
**(Ancestral relationships)** Identify ancestral relationships $Y_{k^{*}}⇝Y_{j}$ if i) $X_{l^{*}}\to Y_{k^{*}}$ for $l^{*}\in A^{\left[h\right]}$ and ii) ${\hat{V}}_{l^{*}j}\neq 0$ where $Y_{j}$ has been previously removed. Update $\hat{𝒮}=\hat{𝒮}\cup \left\{\left(k^{*},j\right)\right\}$.
4. **(Peeling)** Remove leaf variables and associated IVs. Let ${\hat{V}}^{\left[h+1\right]}={\hat{V}}_{\setminus \left(A^{\left[h\right]},B^{\left[h\right]}\right)}^{\left[h\right]}$ where ${\hat{V}}_{\backslash \left(\left(A^{\left[h\right]},B^{\left[h\right]}\right)\right.}^{\left[h\right]}$ is a submatrix by removing the rows and columns indexed by $A^{\left[h\right]}$ and $B^{\left[h\right]}$ from ${\hat{V}}^{\left[h\right]}$.
5. **(Termination)** Let h→h+1 and repeat steps 2–4 until all $Y_{j}$’s are removed. Update $\hat{𝒮}=\hat{𝒮}\cup \left\{(k,j):Y_{k}\to \cdots \to Y_{j}\right.$ in $\left.\hat{𝒮}\right\}$. Compute the causal ordering $\overset{ˆ}{\pi }=\left({\overset{ˆ}{\pi }}_{1},\cdots ,{\overset{ˆ}{\pi }}_{p}\right)$ from $\hat{𝒮}$. Return the ancestors and IVs identified for each $Y_{j}$, $\left(\bar{\text{an}}(j\right)$, $\bar{\text{in}}(j))$.

Algorithm 2 summarizes the peeling process for identifying all ancestral relationships or the causal order among primary variables. We include an illustrative example of the peeling algorithm in Appendix A.1. In Step 3, the peeling algorithm identifies all ancestral relationships via Proposition 3, reconstructing a superset that includes all parent-child relationships. Given the superset, we propose a deconfounding approach to identify parent-child relationships.

### 3.3 Identifying Parent-Child Relationships via Deconfounding

This subsection identifies parent-child relationships given the estimated ancestral relationships from Algorithm 2.

#### 3.3.1 Deconfounding

Given estimated ancestral relationships from the first stage, we develop a novel deconfounding approach based on residual inclusion, called DRI, to estimate parent-child relationships in the presence of confounders. From (2),

(7) $$\psi_{j}\left(E\left(Y_{j}|Y_{\text{pa}(j)},X,h_{j}\right)\right)=U_{\text{pa}(j),j}^{⊤}Y_{\text{pa}(j)}+W_{\text{in}(j),j}^{⊤}X_{\text{in}(j)}+h_{j},\;j=1,\cdots ,p,$$

where $h_{1},\ldots ,h_{p}$ may be correlated. When there is no confounder, we could identify parents by fitting a constrained GLM regression of $Y_{j}$ on its ancestors $Y_{\text{an}(j)}$ and instruments $X_{\text{in}(j)}$. However, in the presence of confounders, unobserved confounders $h_{j}$ and $h_{\text{pa}(j)}$ can be correlated. Thus $Y_{j}$’s parent variables $Y_{\text{pa}(j)}$ depend on $h_{j}$ through $h_{\text{pa}(j)}$, which biases the estimation of $U_{\text{pa}(j),j}$ as $h_{j}$ is one resource of the model error for the regression of $Y_{j}$.

To address the confounding issue, we propose a novel deconfounding approach, DRI, to correct the confounding effects in the child structural equations by treating the residuals from its parent GLM regression as predictors. In this way, this approach utilizes the connections between confounders in a parent and its child equations. To facilitate DRI, we make the practically sensible assumption that the confounders $h_{1},\cdots ,h_{p}$ are jointly normal. Assumption 2 simplifies the implementation of DRI and makes it computationally efficient.

**Assumption 2** *The confounders* $h_{1},\cdots ,h_{p}$ *are jointly normal with an unknown mean and an unknown covariance*.

Remark: Assumption 2 can be relaxed to the assumption that each confounder can be represented as a linear function of other confounders along with an independent error, $h_{j}=\sum_{k}\beta_{kj}h_{k}+\epsilon_{j}$. In the literature, most assume one common underlying confounding (i.e., one h across all equations) while we here consider a more general case of $h_{1},\cdots ,h_{p}$. For complex problems, Assumption 2 is sensible as the confounder is in fact an ensemble of many confounding effects. Under the dense confounding setting in Figure 3, the confounder for each variable $h_{j}$ is added up by many independent confounding effects from unobserved variables. Therefore, asymptotics holds and the confounders are jointly normal by the central limit theorem. In practice, many variables are unobserved and each is associated with many primary variables of interest, satisfying the dense confounding setting.

To implement DRI, we estimate the confounding effect $h_{j}$ using the parent equations for each $Y_{j}$ based on Assumption 2, that is, $h_{j}|\left\{h_{k},k\in \text{an}(j)\right\}\sim N\left(\sum_{k\in \text{an}(j)}\alpha_{kj}h_{k},\sigma^{2}\right)$, or $h_{j}=\sum_{k\in \text{an}(j)}\alpha_{kj}h_{k}+e_{j}$, where $e_{j}\sim N\left(0,\sigma^{2}\right)$ is the unobserved error orthogonal to the projection space spanned by $\left\{h_{k}:k\in \text{an}(j)\right\}$, and uncorrelated with and thus independent of $\left\{h_{k}:k\in \text{an}(j)\right\}$ and $Y_{\text{pa}(j)}$. By Assumption 1(C) and reparameterization (projecting $e_{j}$ onto the space spanned by the non-valid IVs), $e_{j}$ is also independent of X. Then,

(8) $$\psi_{j}\left(E\left[Y_{j}|Y_{\text{pa}(j)},X,h_{j}\right]\right)=U_{\text{pa}(j),j}^{⊤}Y_{\text{pa}(j)}+W_{\text{in}(j),j}^{⊤}X_{\text{in}(j)}+\sum_{k\in \text{an}(j)}\alpha_{kj}h_{k}+e_{j},$$

where DRI replaces $h_{k}$ with the residuals ${\hat{h}}_{k}$ estimated from the parent equations of $Y_{j}$. As a result, $e_{j}$ is independent of $Y_{\text{pa}(j)},X_{\text{in}(j)},\sum_{k\in \text{an}(j)}\alpha_{kj}h_{k}$ in (8), resolving the dependence issue of $Y_{\text{pa}(j)}$ on $h_{j}$ in (7) due to confounding.

We propose a top-down algorithm to estimate parent-child relationships through deconfounding, given the causal ordering of the primary variables $\overset{ˆ}{\pi }$, and $\left(\bar{\text{an}}(j\right)$, $\bar{\text{in}}(j))$, j=1,…,p, identified by Algorithm 2. Note that the causal ordering represents the direction of edges in a DAG in that for every directed edge (k,j), i.e., $Y_{k}\to Y_{j},k$ appears before j in the ordering. The algorithm proceeds from the top to the bottom of a hierarchy defined by the causal order while identifying the parents for each primary variable and iterates this process until the last element of the ordering.

The algorithm starts from a root variable $Y_{k}$ that has no parents. First, a GLM regression of $Y_{k}$ is fit on its valid IVs $X_{\bar{\text{in}}(k)}$ via the model: $\psi_{k}\left(E\left[Y_{k}|X\right]\right)=W_{\bar{\text{in}}(k),k}^{⊤}X_{\bar{\text{in}}(k)}$. Then, we compute the residuals $Y_{ik}-\varphi_{k}\left({\hat{W}}_{\bar{\text{in}}(k),k}^{⊤}X_{i,\bar{\text{n}}(k)}\right)$ to estimate the confounding effect $h_{ik}$, where $\varphi_{k}(\cdot )$ is the inverse link function for the k-th GLM model. It is important to note that the confounders do not bias the estimation of residuals in root equations by the independence assumption of the IVs and confounders. Our simulations and theory suggest that this approach works well, as in the IV regression (Johnston et al. 2008). Alternatively, we can also fit a generalized linear mixed-effects model for root equations when the data has repeated measurements. Details are given in Algorithm 5 of the Appendix.

The algorithm then moves to a non-root variable $Y_{j}$ and considers the GLM regression on its ancestors $Y_{\bar{\text{an}}(j)}$, its IVs $X_{\bar{\text{in}}(j)}$, and the estimated confounder ${\hat{h}}_{k}$ from the ancestor equations via the model: $\psi_{j}\left(E\left[Y_{j}|Y_{\text{pa}(j)},X,h_{j}\right]\right)=U_{\bar{\text{an}}(j),j}^{⊤}Y_{\bar{\text{an}}(j)}+W_{\bar{\text{in}}(j),j}^{⊤}X_{\bar{\text{in}}(j)}+\sum_{k\in \bar{\text{an}}(j)}\alpha_{kj}{\hat{h}}_{k}+e_{j}$, where ($\left.\text{pa}(j),h_{k}\right)$ in (8) is replaced by $\left(\bar{\text{an}}(j),{\hat{h}}_{k}\right)$. Specifically, it fits TLP-constrained GLM regressions:

(9) $$\left({\hat{W}}_{\bar{\text{in}}\left(j\right),j},{\hat{U}}_{\bar{\text{an}}\left(j\right),j},{\hat{\alpha }}_{\bar{\text{an}}\left(j\right),j}\right)=\overset{\text{argmin}}{W_{\bar{\text{in}}\left(j\right),j},U_{\bar{\text{an}}\left(j\right),j,j},\alpha_{\bar{\text{an}}\left(j\right),j}}ℒ\left(W_{\bar{\text{in}}\left(j\right),j},U_{\bar{\text{an}}\left(j\right),j},\alpha_{\bar{\text{an}}\left(j\right),j}|X_{\bar{\text{in}}\left(j\right)},Y_{\bar{\text{an}}\left(j\right)},{\hat{h}}_{\bar{\text{an}}\left(j\right)}\right)\;\text{subject}\;\text{to}\;\underset{k\in \bar{\text{an}}\left(j\right)}{\sum }I\left(U_{kj}\neq 0\right)\leq \bar{K_{j}},\underset{k\in \bar{\text{an}}\left(j\right)}{\sum }I\left(\alpha_{kj}\neq 0\right)\leq \bar{K_{j}^{\prime }},\;j=1,\ldots ,p,$$

where $0\leq \bar{K_{j}}\leq |\bar{\text{an}}(j)|$ and $0\leq \bar{K_{j}^{\prime }}\leq |\bar{\text{an}}(j)|$ can be tuned as in (5), with |⋅| denoting the size of a set; $W_{\bar{\text{in}}(j),j}$ is unconstrained so that Assumption (1)(B) continues to satisfy; $ℒ\left(W_{\bar{\text{in}}(j),j},U_{\bar{\text{an}}(j),j},\alpha_{\bar{\text{an}}(j),j}|X_{\bar{\text{in}}(j)},Y_{\bar{\text{an}}(j)},{\hat{h}}_{\bar{\text{an}}(j)}\right)=n^{-1}\sum_{i=1}^{n}\ell \left(Y_{ij},W_{\bar{\text{in}}(j),j}^{⊤}X_{i,\bar{\text{in}}(j)}+U_{\bar{\text{an}}(j),j}^{⊤}Y_{i,\bar{\text{an}}(j)}+\right.\left.\alpha_{\bar{\text{an}}(j),j}^{⊤}{\hat{h}}_{i,\bar{\text{an}}(j)}\right);h_{i,\bar{\text{an}}(j)}$ denotes a column vector consisting of $\left\{h_{ik}:k\in \bar{\text{an}}(j)\right\}$ and $\alpha_{\bar{\text{an}}(j),j}^{⊤}{\hat{h}}_{i,\bar{\text{an}}(j)}=\sum_{k\in \hat{\text{an}}(j)}{\hat{\alpha }}_{kj}{\hat{h}}_{ik}$. From (9), we obtain the estimated set $\hat{\text{pa}}(j)=\left\{k\in \bar{\text{an}}(j):{\hat{U}}_{kj}\neq 0\right\}\subset \bar{\text{an}}(j)$, and $\hat{\text{in}}(j)=\bar{\text{in}}(j)$. Finally, we compute the residuals

(10) $${\hat{h}}_{ij}=Y_{ij}-\varphi_{j}\left({\hat{U}}_{\hat{\text{pa}}(j),j}^{⊤}Y_{i,\hat{\text{pa}}(j)}+{\hat{W}}_{\hat{\text{in}}(j),j}^{⊤}X_{i,\hat{\text{in}}(j)}+\sum_{k\in \hat{\text{an}}(j)}{\hat{\alpha }}_{kj}{\hat{h}}_{ik}\right).$$

Algorithm 3 summarizes the peeling process for identifying parent-child relationships using the proposed deconfounders.

**Algorithm 3:** Peeling algorithm for estimating parent-child relationships via DRI

1. Input $(\bar{\text{an}}(j),\bar{\text{in}}(j){)}_{j=1}^{p}$ and $\overset{ˆ}{\pi }$ from Algorithm 2. Input data matrix ${\left(Y_{ij},X_{ij}\right)}_{n\times (p+q)}={\left(Y_{i•},X_{i•}\right)}_{i=1}^{n}$ of primary variables $Y_{n\times p}$ and instruments $X_{n\times q}$.
Begin Iteration: for d=1⋯,p,
2. **(Estimating the confounding effects via IV regression)** If ${\overset{ˆ}{\pi }}_{d}$ is a root variable indexed by $Y_{k}$, compute ${\hat{W}}_{\bar{\text{in}}(k),k}$ by fitting a GLM regression of $Y_{k}$ on $X:E\left[Y_{k}|X\right]=\varphi_{k}\left(W_{\bar{\text{in}}(k),k}^{⊤}X_{\bar{\text{in}}(k)}\right)$. Compute the residuals: ${\hat{h}}_{ik}=Y_{ik}-\varphi_{k}\left({\hat{W}}_{\bar{\text{in}}(k),k}^{⊤}X_{i,\bar{\text{in}}(k)}\right)$.
3. **(Deconfounding)** If ${\overset{ˆ}{\pi }}_{d}$ is a non-root variable indexed by $Y_{j}$, compute $\left({\hat{W}}_{\bar{\text{in}}(j),j},{\hat{U}}_{\bar{\text{an}}(j),j},{\hat{\alpha }}_{\bar{\text{an}}(j),j}\right)$ by fitting a TLP-constrained GLM regression of $Y_{j}$ in (9). Compute the residuals ${\hat{h}}_{ij}$ in (10).

Remark: The computational complexity of Algorithm 3 amounts to solving at most p TLP-constrained regressions in (5) of size $\left|\bar{\text{in}}(j)|+2|\bar{\text{an}}(j)\right|$ via Algorithm 1, which is of order $(p+q{)}^{2}\text{max}(n,(p+q))\text{log}K_{j}^{0}$.

#### 3.3.2 Connections with 2 SRI and 2 SPS

DRI is reminiscent of, but fundamentally different from the two-stage predictor substitution (2SPS, (Cai et al. 2011)) and two-stage residual inclusion (2SRI, (Hausman 1978; Terza et al. 2008)), both of which require a known causal order between two primary variables. Similar to 2SRI, our DRI uses estimated residuals as additional predictors in subsequent GLM regressions to deconfound. In 2SRI, the residuals obtained at the first stage serve as an additional predictor as opposed to replacing the endogenous variables with their predicted values in 2SPS, which is also known as two-stage least squares for Gaussian data. However, neither applies to our situation of multiple primary variables with an unknown causal order and different confounders among equations.

For our problem, we also include a version of predictor substitution, referred to as DPS, to compare with DRI in the Appendix. In practice, we recommend DRI for causal discovery due to its superior performance and theoretical guarantees, and therefore integrate it with our top-down peeling algorithm for implementation. DRI explores the connection between parent and child equations to eliminate the confounding effect in a child equation through the residuals, whereas DPS cannot capture this aspect. This recommendation is consistent with the observation of Terza et al. (2008) and Ying et al. (2019) that 2SRI suits more than 2SPS for general nonlinear outcomes, including binary or discrete outcomes in our case. Moreover, in Algorithm 3, we use the residuals from a GLM to estimate the unmeasured confounders. Yet, one may employ different models to estimate the confounders based on their distribution. In Appendix B, we present a general framework of the deconfounding algorithm and then propose a generalized linear mixed model (GLMM) to estimate the confounders when the data has repeated measurements.

## 4. Theory

This section presents a novel theoretical analysis of the proposed approach, offering theoretical guarantees even in the presence of confounders. First, we demonstrate in Theorem 4 that the proposed DC algorithm, Algorithm 1, successfully recovers the true support of $V^{0}$, terminates within a finite number of steps, and achieves a global minimizer for the nonconvex minimization (5), with probability approaching one. Based on this, our bottom-up peeling algorithm, Algorithm 2, retrieves the true super-graph 𝒮. Secondly, we prove in Theorem 5 that our top-down peeling algorithm, Algorithm 3, accurately reconstructs the true causal graph, thereby identifying all parent-child relationships.

Consider a generalized linear model with the canonical link, where the negative log-likelihood of $Y_{ij}$ given $X_{i•}$ based on independent observations ${\left(Y_{ij},X_{i•}\right)}_{i=1}^{n}$ can be expressed as:

(11) $$\ell \left(Y_{ij},\theta \left(X_{i•}\right)\right)=-Y_{ij}\theta \left(X_{i•}\right)+A_{j}\left(\theta \left(X_{i•}\right)\right),\;i=1,\ldots ,n.$$

Here, $A_{j}(\theta )$ represents the cumulant function of an exponential family distribution, with θ denoting the regression function. For instance, in the case of the logistic regression, $A_{j}(\theta )=\text{log}(1+\text{exp}(\theta ))$. Given the canonical link, $A_{j}^{\prime }(\theta )=E\left(Y_{j}|\cdot \right)=\psi_{j}^{-1}(\theta )={\left.\varphi_{j}(\theta )\right|}_{\theta =V_{•j}^{⊤}X_{i•}}$. Hence, the log-likelihood of $Y_{ij}$ given $X_{i•}$ for the fidelity model (3) can be written as:

(12) $$\ell \left(Y_{ij},V_{•j}^{⊤}X_{i•}\right)=-Y_{ij}\left(V_{•j}^{⊤}X_{i•}\right)+A_{j}\left(V_{•j}^{⊤}X_{i•}\right),\;i=1,\ldots ,n.$$

Subsequently, we denote ^0^ as the true parameter; for example, $V^{0}$ means the true parameter values of V. Denote $S_{j}^{0}=\left\{l:V_{lj}^{0}\neq 0\right\}$. Let $K_{j}^{0}={\left\|V_{•j}^{0}\right\|}_{0}=\left|S_{j}^{0}\right|$ and $K_{\text{max}}^{0}=\underset{1\leq j\leq p}{\text{max}}K_{j}^{0}$. The following technical conditions are assumed for the fidelity model (3).

**Assumption 3 (GLM residuals)** *Assume there exists an interval* $\left[K_{1},K_{2}\right]$ *such that* ${V_{•j}^{0}}^{⊤}X_{i•}\in \left[K_{1},K_{2}\right]$. *Further, assume that for any* $\theta \in \left[K_{1}-\epsilon ,K_{2}+\epsilon \right]$ *with some constant* ϵ>0, *there exist some positive constants* $L_{1}$ *and* $L_{2}$, *such that* $\left|A_{j}^{\prime \prime }(\theta )\right|\leq L_{1},\left|A_{j}^{\prime \prime \prime }(\theta )\right|\leq L_{2},j=1,\ldots ,p$, *where* ″ *and* ‴ *denote the second and third derivatives. Moreover*, ${\left\{\xi_{ij}\right\}}_{i=1}^{n}$ *with* $\xi_{ij}=Y_{ij}-E\left[Y_{ij}|\right]$ *is sub*-*exponential with mean zero, so that for any real* t>0,

$$P\left(\left|n^{-1}\sum_{i=1}^{n}\xi_{ij}\right|\geq t\right)\leq 2\;\text{exp}\left(-\text{min}\left(\frac{t^{2}}{2M^{2}},\frac{t}{2M}\right)n\right),\;j=1,\ldots ,p.$$

Note for the fidelity model, $E\left[Y_{ij}|X\right]=\varphi_{j}\left({V_{•j}^{0}}^{⊤}X_{i•}\right)$. Similar conditions have been suggested in Assumption E.1 of Ning and Liu (2017). Assumption 3 includes a wide range of exponential family distributions such as Poisson, and holds for a large class of GLMs including the Poisson regression. In particular, Ning and Liu (2017) and Yang et al. (2015) computed the exact value and thus showed the existence of $L_{1}$ and $L_{2}$ for specific GLMs including the logistic, exponential, and Poisson regressions. For linear and logistic models, Assumption 3 can be relaxed to sub-Gaussian residuals as all sub-Gaussian and bounded variables are sub-exponential (Maurer and Pontil 2021).

**Assumption 4 (Restricted strong convexity)** *For a constant* m>0,

(13) $$\Lambda_{\text{min}}=\underset{A:\left|A\right|\leq 2K_{\text{max}}^{0}}{\text{min}}\underset{\left\{\left(\Delta ,V_{•j}\right):{\left\|\Delta_{A^{c}}\right\|}_{1}\leq 3{\left\|\Delta_{A}\right\|}_{1},V_{•j}\in \left(V_{•j}^{0}-\Delta ,V_{•j}^{0}+\Delta \right)\right\}}{\text{min}}\frac{\Delta^{⊤}\nabla^{2}ℒ\left(V_{•j}\right)\Delta }{\|\Delta {\|}_{2}^{2}}\geq m.$$

Note that (13) is the restricted strong convexity (eigenvalue) condition and requires the log-likelihood $ℒ\left(V_{•j}\right)$ to be strongly convex in a neighborhood of $V_{•j}^{0}$, where $\nabla^{2}ℒ\left(V_{•j}^{0}\right)=X^{⊤}M^{j}X$ and $M^{j}$ is a diagonal matrix with $M_{ii}^{j}=A_{j}^{\prime \prime }\left({V_{•j}^{0}}^{⊤}X_{i•}\right)$ depending on X and $V^{0}$ only. This condition has been commonly used for the analysis of the error bound of parameter estimation and the convergence analysis of optimization algorithms (Lee et al. 2015; Negahban et al. 2012; Hastie et al. 2015; Zhang 2017). Note that Assumption 4 permits correlated designs X and is a weaker condition than the irrepresentable condition required by the Lasso (van de Geer and Bühlmann 2009).

**Assumption 5 (Bounded domain for interventions)** *For some constants* $c_{0}-c_{2}$ *and* $C_{1}>0$,

$$\|X{\|}_{\infty }\leq c_{1},\;{\left\|V_{•j}^{0}\right\|}_{2}\leq C_{1},\;{\left\|{\left(X_{S_{j}^{0}}^{⊤}M^{j}X_{S_{j}^{0}}/n\right)}^{-1}X_{S_{j}^{0}}^{⊤}\right\|}_{\infty }\leq c_{2},\;\Omega_{\text{max}}\left(X_{S_{j}^{0}}^{⊤}X_{S_{j}^{0}}/n\right)\leq c_{0},$$

*where* $\Omega_{\text{max}}(\cdot )$ *refers to the maximum eigenvalue of a matrix*.

Assumption 6 (Minimum signal strength)

$$\underset{V_{lj}^{0}\neq 0}{\text{min}}\left|V_{lj}^{0}\right|\geq 100Mc_{2}\sqrt{\frac{\text{log}\;q+\text{log}\;n}{n}}.$$

Assumption 6 specifies the minimal signal strength over candidate interventions. Such an assumption has been widely used for establishing selection consistency in high-dimensional variable selection (Zhao et al. 2018; Fan and Lv 2011).

**Theorem 4 (Reconstruction of super-graph via** Algorithm 1**)** *Under Assumptions 3-6, for* j=1,…,p, *if the tuning parameters* $\left(\tau_{j},K_{j}\right)$ *of* Algorithm 1 *satisfy*:

1. *(Computation)* $\gamma_{j}\in \left[8\tau_{j}^{-1}\cdot Mc_{1}\sqrt{(\text{log}\;q+\text{log}\;n)/n},m/6\right]$,
2. *(Tuning parameters)* $8Mc_{2}\sqrt{\frac{\text{log}\;q+\text{log}\;n}{n}}\leq \tau_{j}\leq 0.4\;{\text{min}}_{V_{lj}^{0}\neq 0}\left|V_{lj}^{0}\right|,K_{j}=K_{j}^{0}$,

*then* Algorithm 1 *terminates in at most* $1+\left\lceil \text{log}\left(2K_{j}^{0}\right)/\text{log}\;4\right\rceil$ *iterations for* (5), *where* ⌈⋅⌉ *is the ceiling function. Moreover, for* 1≤j≤p,

$$P\left({\tilde{V}}_{•j}\;\text{is}\;\text{not}\;\text{a}\;\text{global}\;\text{minimizer}\;\text{of}\;(5)\right)\leq 8q\;\text{exp}(-2(\text{log}(q)+\text{log}(n)))=8q^{-1}n^{-2}.$$

*As a result*, Algorithm 1 *yields a global minimizer of* (5), ${\tilde{V}}_{•j}$, *with probability tending to 1 as* n→∞. *Importantly*, Algorithm 1, *together with Algorithm 2, recovers the true super*-*graph* ${𝒮}^{0}$ *containing ancestral relations with probability*

$$P\left(\hat{𝒮}\neq {𝒮}^{0}\right)\leq 8pq^{-1}n^{-2},$$

*where* $\hat{𝒮}$ *is obtained from* Algorithm 2 *and* ${𝒮}^{0}\equiv \{(k,j):k\in \text{an}(j)\}$. *Under Assumption 1(C) (i.e*., p≤q), *with probability tending to one*, $\hat{𝒮}$ *correctly reconstructs the true super*-graph ${𝒮}^{0}$ and thus the causal order of $Y_{1},\ldots ,Y_{p}$ *as* n→∞.

Theorem 4 ensures the consistent reconstruction of the super-graph ${𝒮}^{0}$ by Algorithm 1 and Algorithm 2, which characterizes ancestral relationships and determines the causal order of primary variables. Also, it says that Algorithm 1 (DC algorithm) attains a global minimizer almost surely as n→∞ under the data generating distribution. This result is in contrast to the strong hardness result of Chen et al. (2019) that there does not exist a polynomial-time algorithm achieving the globality of the $\ell_{0}$-constrained optimization (5) in the worst-case scenario. We here show that with probability tending to one, this problem can be solved. In other words, the probability of the worst-case scenario tends to zero. Note that Algorithm 1 is indeed a polynomial-time algorithm with time complexity $O\left(q^{2}\text{max}(q,n)\text{log}K_{j}^{0}\right)$ for solving one $\ell_{0}$-constrained regression in (5). In addition, since $K_{j}$ is discrete, the assumption $K_{j}=K_{j}^{0}$ corresponds to the requirement that the optimal parameter λ for the Lasso has to be within a range of values for consistency. In practice, $K_{j}^{0}$ is unknown and $K_{j}$ is tuned via parameter selection methods.

Next, we establish causal graph selection consistency of the estimated causal graph based on the estimates ${\hat{U}}_{•j}$ by Algorithm 3. On this ground, we ensure that all parent-child relationships are correctly identified. Let ${\bar{K_{j}}}^{0}={\left\|U_{•j}^{0}\right\|}_{0},{\bar{K_{j}^{\prime }}}^{0}=|\text{an}(j)|,s={\text{max}}_{1\leq j\leq p}|\text{an}(j)|$, $\tilde{s}={\text{max}}_{1\leq j\leq p}{\left\|W_{•j}^{0}\right\|}_{0}$, and $\tilde{Z}=\left[X_{\text{in}(j)},Y_{\text{pa}(j)},{\hat{h}}_{\text{an}(j)}\right]$. Under Assumption 5 with $\tilde{Z},{\left\|{\tilde{Z}}^{⊤}\right\|}_{\infty }\leq b_{1},{\left\|{\left({\tilde{Z}}^{⊤}M\tilde{Z}/n\right)}^{-1}{\tilde{Z}}^{⊤}\right\|}_{\infty }\leq b_{2}$, and $\Omega_{\text{max}}\left({\tilde{Z}}^{⊤}\tilde{Z}/n\right)\leq b_{0}$.

**Theorem 5 (Reconstruction of causal graph via** Algorithm 3**)** *Under Assumptions 3-5 with* $\tilde{Z}=\left[X_{in(j)},Y_{pa(j)},{\hat{h}}_{\text{an(j)}}\right]$ *in the GLM regression (9), if tuning parameters of* Algorithm 3 *satisfy*:

1. *(Computation)* $\gamma_{j}\in \left[\tau_{j}^{-1}\cdot 8Mb_{1}\sqrt{(\text{log}(2s+\tilde{s})+\text{log}\;p)/n},m/6\right]$,
2. *(Tuning parameters)* $C\sqrt{\frac{\text{log}(2s+\tilde{s})+\text{log}\;p}{n}}\leq \tau_{j}\leq 0.4\;{\text{min}}_{U_{kj}^{0}\neq 0}\left|U_{kj}^{0}\right|,\bar{K_{j}}={\bar{K_{j}}}^{0},\bar{K_{j}^{\prime }}={\bar{K_{j}^{\prime }}}^{0}$,

*where* C *is a constant depending on* $b_{1},b_{2}$ *and* $b_{0}$, *then*, Algorithm 3 *reconstructs the causal graph consistently with probability tending to one, or*

$$P\left(\hat{E}=E^{0}\right)\to 1,\text{as}\;n\to \infty ,$$

*where* $\hat{E}=\left\{(k,j):{\hat{U}}_{kj}\neq 0\right\}$ *and* $E^{0}=\left\{(k,j):U_{kj}^{0}\neq 0\right\}$.

Theorem 5 suggests that Algorithm 3 recovers the true causal graph and thus causal relationships with probability tending to one as the sample size is sufficiently large. In Appendix D.5, we prove this by establishing error bounds of the estimates ${\hat{U}}_{•j},{\hat{W}}_{•j}$ for estimating U and W by Algorithm 3.

Remark: By Theorem 4 and 5, our proposed GAMPI using Algorithm 1–3 reconstructs the causal graph consistently with probability at least $1-8pq^{-1}n^{-2}-8(2s+\tilde{s}{)}^{-1}p^{-1}$. For fixed p case, the logp term in $\gamma_{j}$ and $\tau_{j}$ of Theorem 5 can be modified to log(np) respectively and the probability is then $1-8pq^{-1}n^{-2}-8(2s+\tilde{s}{)}^{-1}n^{-2}p^{-1}$, similar to the remarks of Ravikumar et al. (2010).

## 5. Simulations

This section investigates the empirical performance of the proposed method. We assess the performance of GAMPI and compare it against the structure learning method NOTEARS (Zheng et al. 2018, 2020), under various graph structures (hub, chain, and random graphs) and types of outcome variables. Further, we compare GAMPI with a recently proposed structure learning method DAGMA (Bello et al. 2022) based on a log-det constraint. For NOTEARS and DAGMA, we use the loss type that is appropriate for the data type of the outcome variables. Note that DAGMA is designed exclusively for Gaussian and logistic outcomes.

### 5.1 Simulation Setting

The data simulation process is as follows. Firstly, we generate an adjacency matrix U based on the graph structure and construct an intervention matrix W with $W_{jj}=1$, for j=1,⋯,p and $W_{lj}=0$, for 1≤l≠j≤q. For the hub graph, $U_{1j}=1,j=2,\cdots ,p$, and 0 otherwise. The random graph is simulated similarly as Li et al. (2023). Secondly, we generate Gaussian instrumental variables $X=\left(X_{1},\ldots ,X_{q}\right)\sim N(0,1)$. We also investigate the case when the instrumental variables X are correlated in Appendix C.7. For the confounders, we simulate h~N(0,Σ), where $\rho_{ij}=0.95$. In Appendix C.1, we explore the simulation setup where the data is generated without confounders, i.e., h=0. Given X,U,W, and h, we generate random samples Y according to (2). In this section, we consider two data types for the outcome variable Y: binary and count outcomes. In the binary case, $Y_{j}$ is generated from the Bernoulli distribution with $P\left(Y_{j}=1\right)$ equal to $\frac{\text{exp}\left(\alpha_{0}w_{j}^{⊤}X_{\text{in}(j)}+h_{j}\right)}{1+\text{exp}\left(\alpha_{0}w_{j}^{⊤}X_{\text{in}(j)}+h_{j}\right)}$ if $Y_{j}$ is a root variable, and $\frac{\text{exp}\left(\beta_{1}u_{j}^{⊤}Y_{\text{pa}(j)}+\alpha_{1}w_{j}^{⊤}X_{\text{in}(j)}+h_{j}\right)}{1+\text{exp}\left(\beta_{1}u_{j}^{⊤}Y_{\text{pa}(j)}+\alpha_{1}w_{j}^{⊤}X_{\text{in}(j)}+h_{j}\right)}$ otherwise. For the hub graph, we set $\alpha_{0}=5,\beta_{1}=2.5$, and $\alpha_{1}=2$. For the chain graph, we set $\alpha_{0}=5,\beta_{1}=2.5$, and $\alpha_{1}=3$. For the random graph, we set $\alpha_{0}=5,\beta_{1}=3$, and $\alpha_{1}=3$.

For the count outcome, to avoid extreme values, we employ standard copula transforms to simulate Y, as described by Yang et al. (2015) and Nelsen (2007). Specifically, we first generate data using ${\tilde{Y}}_{j}=\beta_{1}u_{j}^{⊤}Y_{\text{pa}(j)}+\alpha_{1}w_{j}^{⊤}X_{\text{in}(j)}+h_{j}+\epsilon_{j}$, where $\epsilon_{j}$ are i.i.d. Gaussian errors. We then use a standard copula transform to ensure that the marginals of the generated data $Y_{j}$ are approximately Poisson. For the hub graph, we set $\alpha_{0}=5,\beta_{1}=0.5$, and $\alpha_{1}=2$. For the chain graph, we set $\alpha_{0}=5,\beta_{1}=0.5$, and $\alpha_{1}=3$. For the random graph, we set $\alpha_{0}=4,\beta_{1}=1$, and $\alpha_{1}=2$. We consider three different graph structures: the hub, chain (of length 4), and random graphs. In addition, we fix the sample size n=500 while varying the number of variables from 100 to 300.

To evaluate the accuracy of estimating the directed edges of a graph, we consider five evaluation metrics: the false positive rate (FPR), the false discovery rate (FDR), the F-score, the Matthews correlation coefficient (MCC), and the structural Hamming distance (SHD). The Matthews correlation coefficient is a binary classification metric defined as

$$\frac{\text{TP}\times \text{TN}-\text{FP}\times \text{FN}}{\{(\text{TP}+\text{FP})(\text{TP}+\text{FN})(\text{TN}+\text{FP})(\text{TN}+\text{FN}){\}}^{1/2}},$$

where TP, FP, TN and FN denote the true positive, false positive, true negative, and false negative rates for edge selection. A large MCC value close to 1 indicates that the estimated edge set is close to the true edge set. In addition, the structural Hamming distance measures edge directionality between two directed graphs, which is the number of edge insertions, deletions, or flips needed to transform one graph to another graph (Tsamardinos et al. 2006). A small structural Hamming distance between two graphs of the same size indicates their closeness.

### 5.2 Results

This subsection reports the simulation results in a situation where we simulate the data in the presence of confounders. Table 2 suggests that GAMPI outperforms NOTEARS across all setups in terms of causal graph recovery, as measured by five metrics: FPR, FDR, F-score, MCC, and SHD. Table 2 shows that NOTEARS can yield an empty graph with no edges selected when “NA” occurs. Table 6 in Appendix C.4 suggests that GAMPI outperforms DAGMA significantly in most scenarios, except for the simple case of the hub graph, where both methods perform equally well. Further, note that unlike GAMPI, NOTEARS and DAGMA do not guarantee acyclicity or estimate the parameters of causal effects. To conclude, our simulation results demonstrate the advantage of the proposed method for causal graph recovery in the presence of confounders.

In Appendix C.2, we further compare our deconfounding approach via DRI with employing the standard GLM in Algorithm 3 for binary outcomes. For the chain graph, the standard logistic regression without adjusting for confounders does not perform well in terms of causal discovery. This is because the unobserved confounders induce false positive edges between the node and its ancestors. By contrast, the deconfounding approach corrects the bias of the confounders and recovers the true graph structure. For the hub graph, though both two approaches recover the true causal graph, the confounding approach still outperforms the standard logistic regression in terms of parameter estimation. Note that our peeling algorithm in the first stage identifies the correct ancestral relationships (super-graph) as the confounders are independent of the instrumental variables by assumption.

In addition, in Appendix C.1, we consider the special case when the data is simulated without confounders. The result suggests that our deconfounding approach performs well even when the data is simulated without confounders. Last, we consider the simulation setup where the data has repeated measurements. Still, our deconfounding approach using a mixed-effects model outperforms the standard GLM approach. To summarize, our deconfounding approach demonstrates strong empirical performance and outperforms the existing methods in most cases.

## 6. Mixed DAG Networks: Direct Effect to AD

This section applies GAMPI to a publicly available Alzheimer's Disease Neuroimaging Initiative (ADNI) dataset. Our goal is to estimate a regulatory gene expression network of a subset of genes related to Alzheimer's disease (AD) and identify which of the genes have a direct causal effect on AD through gene-to-gene and gene-to-AD regulatory networks.

First, we download the raw data from the ANDI website (https://adni.loni.usc.edu), containing gene expression, DNA sequencing, and phenotypic data. Then, for preprocessing, we clean and merge these data to obtain 712 subjects with complete records. In addition, from the KEGG database (Kanehisa et al. 2002), we extract the AD reference pathway (hsa05010, https://www.genome.jp/pathway/hsa05010) and therefore obtain 146 genes from the ANDI data. Meanwhile, the subjects are categorized into four groups: Cognitive Normal (CN), Early Mild Cognitive Impairment (EMCI), Late Mild Cognitive Impairment (LMCI), and Alzheimer's Disease (AD). We treat the 247 CN individuals as the control group and the remaining 465 AD and MCI individuals as the case group. We then include the disease status, a binary outcome with 0/1 indicating normal/AD, as an additional variable (node) to identify which genes are directly related to AD. Moreover, we use SNPs as instrumental variables in this case study as it is known that biologically SNPs may have an impact on the genes, but not the other way around, therefore satisfying the IV requirement.

To perform data analysis, we first regress the gene expressions on the additional covariates, including age, gender, education, handedness, and intracranial volume. Next, for each SNP from a gene, we perform significance tests with the gene and disease status marginally and select the genes which have at least one SNP whose i) significance level with the gene is less than 0.05 and ii) significance level with the disease status is less than 0.02, rendering p=39 primary variables. For these genes, we extract their two most correlated SNPs with the disease status based on the p-values given the significance level with the gene less than 0.05, yielding q=39×2=78 instrumental variables. Removing duplicate SNPs and the gene that has the same SNPs as other genes results in p=38 and q=76. To summarize, we use the gene expressions along with the disease status as primary variables and SNPs as instrumental variables to reconstruct a causal network for gene-to-gene and gene-to-disease regulatory relationships.

**Figure 2:**
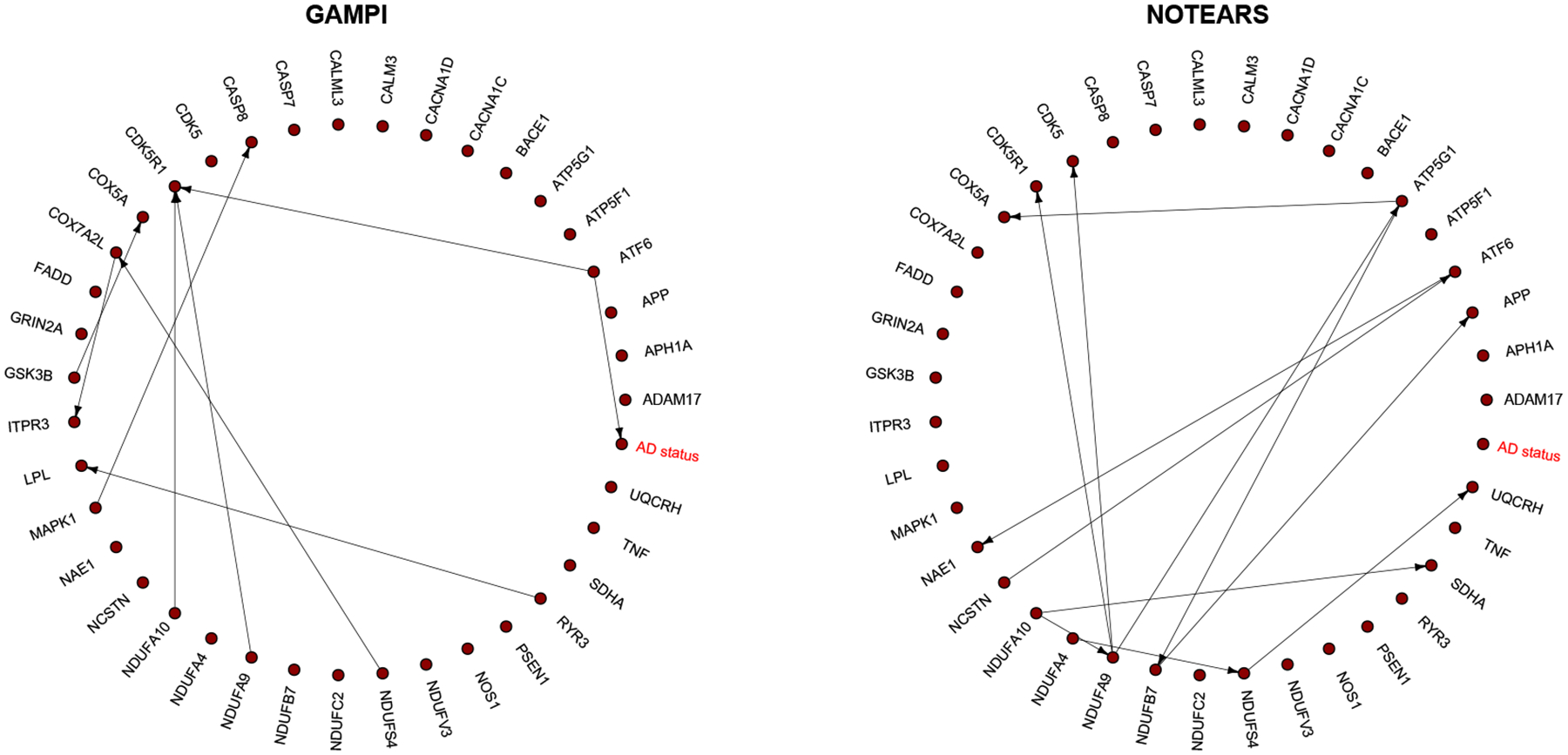
Reconstructed gene-to-gene and gene-to-AD regulatory network. “AD status” is a binary outcome with 0/1 indicating normal/AD. Directed edges indicate causal relationships identified by the proposed GAMPI (left) and existing method NOTEARS (right).

As shown in Figure 2, GAMPI identifies a direct causal effect of gene ATF6 on the AD status. In the literature, ATF6 is a transcription factor that acts during endoplasmic reticulum (ER) stress by activating UPR target genes, and ER stress is known to be closely associated with AD. Furthermore, Du et al. (2020) suggested that ATF6 could be a potential hub for targeting the treatment of AD, which protects the retention of spatial memory in AD model mice. Zhang et al. (2022) found that the expression of both ATF6 and CTH are decreased in AD patients and ATF6 positively regulates the expression of CTH so that the addition of CTH reduces the loss of spatial learning and memory ability in mice caused by ATF6 reduction. In addition, GAMPI uncovers some known regulatory relationships related to AD in the literature for both the AD and control groups. For example, for the directed connection MAPK1 → CASP8, it has been shown that phosphorylation of p38 MAPK induced by oxidative stress is associated with the activation of caspase-8-mediated apoptotic pathways in dopaminergic neurons (Choi et al. 2004). The connection ATF6 → CDK5R1 is in the AD KEGG pathway https://www.genome.jp/pathway/hsa05010. Furthermore, the approach also identifies some potential gene regulatory relationships for future biological investigations. For example, the two genes in the connection RYR3 → LPL are among the 13 genes directly associated with AD in the DEX DFC geneset analysis (Sharma et al. 2021), while the two genes in the connection GSK3B → COX5A are in the same AD-related protein association network in AD-iPS5 neurons (Hossini et al. 2015). Further, we compare our proposed method GAMPI with the existing method NOTEARS. Both find common gene-to-gene causal relationship NDUFA9 → CDK5R1. Moreover, our proposed method identifies the meaningful gene-to-disease regulatory relationship validated biologically in the literature.

## 7. Discussion

The article introduces a new causal discovery approach, GAMPI, which reconstructs a directed acyclic graph using instruments in the presence of unmeasured confounders. GAMPI involves generalized structural equation models that are identifiable with the help of instruments under certain conditions. GAMPI involves two steps. First, we proposed a fidelity model that is also a generalized linear model, having the same support as the marginal model regarding instrumental interventions. On this ground, we designed a bottom-up peeling algorithm to identify ancestral relationships and valid instruments by exploiting the connection between primary and instrumental variables. In the second step, we proposed a deconfounding approach to further select parent-child relationships from the identified ancestral relationships. This approach estimates the confounding effects from the parenťs equations and uses them in subsequent child equations to correct the confounding effects. The theoretical properties of GAMPI are also analyzed, including the globality of the DC solution for nonconvex minimization, estimation accuracy, and causal graph selection consistency. A series of simulation results demonstrate causal graph selection consistency and the practical advantages of GAMPI for handling unmeasured confounders and non-Gaussian outcomes.

Overall, GAMPI provides a promising approach to causal discovery, with potential applications in various fields beyond Alzheimer's disease. For instance, the method can be used to explore causal relationships in complex systems with unmeasured confounders, such as in economics or public health. Furthermore, GAMPI's flexibility to adapt to different distributions of confounders and link functions makes it suitable for a wide range of scenarios. For instance, it can handle directed graphical models with mixed variables (Chowdhury et al. 2022). In conclusion, GAMPI offers a valuable contribution to causal inference by providing a practical method for identifying causal relationships under challenging situations.

The R implementation is available at https://github.com/minjie-wang/GAMPI.

## Supplementary Material

1

## Figures and Tables

**Table 1:** Examples of distributions in generalized linear models

Distribution	Support	Link	Density
Bernoulli, Bern(μ)	Integer: {0,1}	$\psi_{j}(\mu )=\text{ln}\left(\frac{\mu }{1-\mu }\right)$	$\mu^{y}(1-\mu {)}^{1-y}$
Binomial, Bin(N,μ)	Integer: 0,…,N	$\psi_{j}(\mu )=\text{ln}\left(\frac{\mu }{1-\mu }\right)$	Nyμy(1−μ)N−y
Gaussian, $N\left(\mu ,\sigma^{2}\right)$	Real: (-∞,∞)	$\psi_{j}(\mu )=\mu$	$\frac{1}{\sqrt{2\pi \sigma^{2}}}\text{exp}\left(-\frac{(y-\mu {)}^{2}}{2\sigma^{2}}\right)$
Poisson, Poisson(μ)	Integer: 0,1,…	$\psi_{j}(\mu )=\text{ln}\mu$	$\frac{\mu^{y}\;\text{exp}\left(-\mu \right)}{y!}$
Multinomial, $\text{Multi}\left(\mu_{1},\ldots ,\mu_{K}\right)$	K-vector of integer: [0,…,N]	$\psi_{j}(\mu )=\text{ln}\left(\frac{\mu }{1-\mu }\right)$	$\frac{n!}{y_{1}!\ldots y_{K}!}\prod_{k=1}^{K}\mu_{k}^{y_{k}}$

**Table 2:** Comparison of causal graph reconstruction accuracy of GAMPI and NOTEARS in the presence of confounders, with GAMPI employing EBIC for tuning parameter selection and NOTEARS applying the default value of 0.1. Metrics include FPR, FDR, F-score, MCC, and SHD. NA indicates that the method returns an empty graph with no edges selected.

Binary											
Graph	(p,q,n)	FPR		FDR		F-score		MCC		SHD	
		No-tears	GAMPI	No-tears	GAMPI	No-tears	GAMPI	No-tears	GAMPI	No-tears	GAMPI
Hub	(100,100,500)	0.00 (0.00)	0.00 (0.00)	0.01 (0.01)	0.05 (0.01)	0.16 (0.01)	0.96 (0.01)	0.29 (0.01)	0.96 (0.01)	90.30 (0.78)	8.10 (1.46)
	(200,200,500)	0.00 (0.00)	0.00 (0.00)	0.01 (0.01)	0.04 (0.01)	0.13 (0.02)	0.95 (0.01)	0.25 (0.03)	0.95 (0.01)	184.90 (2.37)	20.40 (3.95)
	(300,300,500)	0.00 (0.00)	0.00 (0.00)	0.00 (0.00)	0.04 (0.01)	0.21 (0.02)	0.95 (0.01)	0.34 (0.02)	0.95 (0.01)	263.50 (3.87)	28.20 (7.61)
Chain	(100,100,500)	0.00 (0.00)	0.00 (0.00)	NA (NA)	0.16 (0.02)	NA (NA)	0.87 (0.01)	0.00 (0.00)	0.87 (0.01)	75.00 (0.00)	21.00 (2.72)
	(200,200,500)	0.00 (0.00)	0.00 (0.00)	NA (NA)	0.21 (0.01)	NA (NA)	0.84 (0.01)	0.02 (0.01)	0.84 (0.01)	149.80 (0.13)	52.30 (2.31)
	(300,300,500)	0.00 (0.00)	0.00 (0.00)	NA (NA)	0.22 (0.01)	NA (NA)	0.83 (0.01)	0.01 (0.01)	0.83 (0.01)	224.80 (0.13)	84.30 (5.17)
Random	(100,100,500)	0.00 (0.00)	0.00 (0.00)	NA (NA)	0.14 (0.01)	NA (NA)	0.74 (0.02)	0.08 (0.02)	0.74 (0.02)	73.00 (1.56)	33.90 (1.98)
	(200,200,500)	0.00 (0.00)	0.00 (0.00)	0.52 (0.08)	0.17 (0.01)	0.03 (0.01)	0.69 (0.01)	0.09 (0.01)	0.70 (0.01)	147.90 (5.32)	78.40 (3.25)
	(300,300,500)	0.00 (0.00)	0.00 (0.00)	0.61 (0.04)	0.26 (0.01)	0.03 (0.00)	0.64 (0.00)	0.07 (0.01)	0.65 (0.00)	224.30 (6.86)	144.00 (3.69)
Count											
Graph	(p,q,n)	FPR		FDR		F-score		MCC		SHD	
		No-tears	GAMPI	No-tears	GAMPI	No-tears	GAMPI	No-tears	GAMPI	No-tears	GAMPI
Hub	(100,100,500)	0.00 (0.00)	0.00 (0.00)	NA (NA)	0.00 (0.00)	NA (NA)	1.00 (0.00)	0.00 (0.00)	1.00 (0.00)	99.00 (0.00)	0.30 (0.30)
	(200,200,500)	0.00 (0.00)	0.00 (0.00)	NA (NA)	0.00 (0.00)	NA (NA)	1.00 (0.00)	0.00 (0.00)	1.00 (0.00)	199.00 (0.00)	1.40 (1.19)
	(300,300,500)	0.00 (0.00)	0.00 (0.00)	NA (NA)	0.00 (0.00)	NA (NA)	1.00 (0.00)	0.00 (0.00)	1.00 (0.00)	299.00 (0.00)	2.50 (0.79)
Chain	(100,100,500)	0.00 (0.00)	0.00 (0.00)	NA (NA)	0.00 (0.00)	NA (NA)	0.95 (0.00)	0.00 (0.00)	0.95 (0.00)	75.00 (0.00)	7.30 (0.58)
	(200,200,500)	0.00 (0.00)	0.00 (0.00)	NA (NA)	0.00 (0.00)	NA (NA)	0.94 (0.01)	0.00 (0.00)	0.94 (0.01)	150.00 (0.00)	17.80 (1.58)
	(300,300,500)	0.00 (0.00)	0.00 (0.00)	NA (NA)	0.00 (0.00)	NA (NA)	0.92 (0.00)	0.00 (0.00)	0.92 (0.00)	225.00 (0.00)	32.60 (1.45)
Random	(100,100,500)	0.00 (0.00)	0.00 (0.00)	NA (NA)	0.00 (0.00)	NA (NA)	0.92 (0.01)	0.00 (0.00)	0.92 (0.01)	73.00 (2.93)	11.10 (1.28)
	(200,200,500)	0.00 (0.00)	0.00 (0.00)	NA (NA)	0.01 (0.00)	NA (NA)	0.89 (0.01)	0.00 (0.00)	0.89 (0.01)	154.60 (3.88)	31.40 (2.79)
	(300,300,500)	0.00 (0.00)	0.00 (0.00)	NA (NA)	0.00 (0.00)	NA (NA)	0.88 (0.01)	0.00 (0.00)	0.89 (0.01)	228.60 (2.93)	49.20 (2.45)
