# Supplementary material for: Causal Discovery with Generalized Linear Models through Peeling Algorithms
Source: J Mach Learn Res. Author manuscript; Available in PMC 2025 Jan 4. (PMC11699566)
Supplement: 1 [file NIHMS2044931-supplement-1.pdf]

Appendix A. Illustrative Examples

In this section, we delve into detailed examples that elucidate the peeling algorithm, the majority rule for a linear link as outlined in Assumption 1(B), and the dense confounding setting justifying Assumption 2.

A.1 Peeling Algorithm

We illustrate the peeling algorithm (Algorithm 2) with the motivating example in (4). From (4), we generate the data of sample size $n = 500$ and compute $\widehat{\boldsymbol{V}}$ using Algorithm 1. The estimated $\widehat{\boldsymbol{V}}$ is:

$$\widehat{\boldsymbol{V}}_{q\times p} = \begin{pmatrix} 2.06 & 0.35 & 0.00 & -0.35 & 0.00 \\ 0.00 & 1.84 & 0.46 & 0.00 & 0.00 \\ 0.00 & 0.00 & 1.77 & 0.39 & 0.00 \\ 0.00 & 0.00 & 0.00 & 1.76 & 0.00 \\ 0.00 & 0.00 & 0.00 & 0.00 & 1.96 \end{pmatrix}.$$

Algorithm 2 proceeds as follows.

- *Iteration 1:* X_4 is identified as an instrument of leaf node Y_4 ($X_4 \to Y_4$) as row 4 has the smallest row-wise ℓ_0-norm and $\widehat{V}_{44}$ is the only nonzero item in row 4.

  X_5 is identified as an instrument of leaf node Y_5 ($X_5 \to Y_5$) as row 5 has the smallest row-wise ℓ_0-norm and $\widehat{V}_{55}$ is the only nonzero item in row 5.

  Y_4, Y_5, X_4, and X_5 are removed.

- *Iteration 2:* X_3 is identified as an instrument of leaf node Y_3 ($X_3 \to Y_3$) in the subgraph for Y_1, Y_2 and Y_3 as row 3 has the smallest row-wise ℓ_0-norm of the submatrix for Y_1, Y_2 and Y_3, with $\widehat{V}_{33}$ the only nonzero item in row 3. Moreover, since $\widehat{V}_{34} \neq 0$ and Y_4 is removed in the previous iteration, $Y_3 \rightsquigarrow Y_4$.

Y_3 and X_3 are removed.

- *Iteration 3:* Similarly, X_2 is identified as an instrument of leaf node Y_2 ($X_2 \to Y_2$) in the subgraph for Y_1, Y_2. Moreover, since $\widehat{V}_{23} \neq 0$ and Y_3 is removed in the previous iteration, $Y_2 \rightsquigarrow Y_3$.

  Y_2 and X_2 are removed.

- *Iteration 4:* Similarly, X_1 is identified as an instrument of leaf node Y_1 ($X_1 \to Y_1$). Moreover, since $\widehat{V}_{12}$, $\widehat{V}_{14} \neq 0$ and Y_2, Y_4 are removed in the previous iterations, $Y_1 \rightsquigarrow Y_2$ and $Y_1 \rightsquigarrow Y_4$.

  Y_1 and X_1 are removed and the peeling process is terminated.

Finally, step 5 adds ancestral relations: $Y_1 \rightsquigarrow Y_3$ and $Y_2 \rightsquigarrow Y_4$. To conclude, Algorithm 2 identifies ancestral relationships: $Y_1 \rightsquigarrow Y_2$, $Y_1 \rightsquigarrow Y_3$, $Y_1 \rightsquigarrow Y_4$, $Y_2 \rightsquigarrow Y_3$, $Y_2 \rightsquigarrow Y_4$ and $Y_3 \rightsquigarrow Y_4$.

A.2 Majority Rule

Consider the following example of a generalized structural equation model:

$$\begin{aligned} &\psi_1(\mathbb{E}[Y_1|X_1, X_2, X_3, h_1]) = W_{11}X_1 + W_{21}X_2 + W_{31}X_3 + h_1, \\ &\psi_2(\mathbb{E}[Y_2|Y_1, X_2, X_3, X_4, h_2]) = U_{12}Y_1 + W_{22}X_2 + W_{32}X_3 + W_{42}X_4 + h_2, \end{aligned} \tag{14}$$

where X_1 is an valid IV of Y_1, X_4 is an valid IV of Y_2, and X_2, X_3 are invalid IVs. Note if ψ_1 is linear, then (14) is not identifiable as the majority rule is not satisfied (Kang et al. 2016; Windmeijer et al. 2019). If ψ_1 is non-linear, then the linear effect of the instruments in the first equation cannot be represented by the one in the second equation. Hence, identifiability is achieved through non-linearity and the majority rule is not required (details are given in the proof of Proposition 1).

A.3 Dense Confounding Setting

This section illustrates the dense confounding setting justifying Assumption 2, where the confounder for each variable h_j is added up by many independent confounding effects from unobserved variables. Therefore, asymptotics holds and the confounders are jointly normal by the central limit theorem.

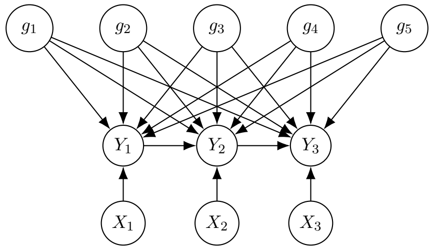

Figure 3: Dense confounding setting, where the confounder for each variable h_j is added up by many independent confounding effects from unobserved variables. For example, in this case, the confounders can be re-parameterized as: $h_1 = a_{11}g_1 + a_{21}g_2 + a_{31}g_3 + a_{41}g_4 + a_{51}g_5$, etc.

Appendix B. General Form of Deconfounding Algorithm

Algorithm 4 serves as a general version of Algorithm 3 in the main paper for estimating parent-child relationships in the presence of confounders. In Algorithm 3 of the main paper, we utilize residuals from a GLM to impute confounders. The underlying intuition of this deconfounding approach is to achieve accurate parameter estimates and construct a consistent estimate of unmeasured confounders

via residuals through the root equations. In this regard, different models can be employed to estimate confounding effects in the root equations, as described in Algorithm 4. For instance, the marginal likelihood that integrates the confounding effect h_k from the complete likelihood can be applied, in addition to the Markov Chain Monte Carlo approach (Knudson et al. 2021).

Algorithm 4: General peeling algorithm for estimating parent-child relationships via DRI

1. Input $(\overline{\text{an}}(j), \overline{\text{in}}(j))_{j=1}^p$ and $\hat{\pi}$ from Algorithm 2. Input data matrix $(Y_{ij}, X_{ij})_{n\times(p+q)} = (\boldsymbol{Y}_{i\bullet}, \boldsymbol{X}_{i\bullet})_{i=1}^n$ of primary variables $\boldsymbol{Y}_{n\times p}$ and instruments $\boldsymbol{X}_{n\times q}$.
Begin Iteration: for $d = 1\cdots,p$,
2. **(Estimation of confounding effects)** If $\hat{\pi}_d$ is a root variable indexed by Y_k, obtain an estimate of the confounding effect $\widehat{h}_{ik}$.
3. **(Deconfounding)** If $\hat{\pi}_d$ is a non-root variable indexed by Y_j, compute $(\widehat{\boldsymbol{W}}_{\overline{\text{in}}(j),j}, \widehat{\boldsymbol{U}}_{\overline{\text{an}}(j),j}, \widehat{\boldsymbol{\alpha}}_{\overline{\text{an}}(j),j})$ by fitting a TLP-constrained GLM regression of Y_j in (9). Compute the residuals $\widehat{h}_{ij}$ in (10).

Specifically, when the data has repeated measurements, we propose to use a generalized linear mixed model (GLMM) to estimate the unmeasured confounders in the root equations in Algorithm 5 as an alternative to Algorithm 3 in the main paper. Consider the structural equation model:

$$\psi_j(\mathbb{E}\left(\boldsymbol{Y}_{ij}|\boldsymbol{Y}_{i,\text{pa}(j)}, \boldsymbol{X}_{i\bullet}, h_{ij}\right)) = \boldsymbol{Y}_{i,\text{pa}(j)}\boldsymbol{U}_{\text{pa}(j),j} + \boldsymbol{X}_{i,\text{in}(j)}\boldsymbol{W}_{\text{in}(j),j} + h_{ij}\mathbf{1}_{n_i}, \quad j = 1,\cdots,p. \tag{15}$$

Here, $i = 1,\cdots,N$ represents a group index with n_i observations within a group. For each group, we observe an $n_i \times 1$ vector of responses, $\boldsymbol{Y}_{ij}$ and hence an $n_i \times p$ matrix, $\boldsymbol{Y}_{i\bullet}$. Let $\boldsymbol{X}_{i\bullet}$ be an $n_i \times q$ fixed-effects design matrix, $\boldsymbol{W}_{\text{in}(j),j}$ an $|\text{in}(j)| \times 1$, and $\boldsymbol{U}_{\text{pa}(j),j}$ a $|\text{pa}(j)| \times 1$ column vector of fixed regression coefficients. Further, h_{ij} denotes a group-specific vector of random intercepts.

Similarly, we adopt a two-stage deconfounding procedure to estimate parent-child relationships, with a GLMM in the root equation for improved confounder estimation. Specifically, we fit a GLMM on variable Y_k using instrumental variables $\boldsymbol{X}_{\text{in}(k)}$ and estimate confounding effects $\{\widehat{h}_{ik}\}_{i=1}^N$ via the estimated random effects. In the child equation, we impute unmeasured confounders using estimated values from the parent equation and fit a TLP-constrained GLM, similar to the previous approach.

Algorithm 5: Peeling algorithm for estimating parent-child relationships in the presence of confounders using GLMM and DRI

1. Input $(\overline{\text{an}}(j), \overline{\text{in}}(j))_{j=1}^p$ and $\hat{\pi}$ from Algorithm 2. Input $(\boldsymbol{Y}_{ij}, \boldsymbol{X}_{ij})_{n\times(p+q)} = (\boldsymbol{Y}_{i\bullet}, \boldsymbol{X}_{i\bullet})_{i=1}^N$ of primary variables $\boldsymbol{Y}_{n\times p}$ and instruments $\boldsymbol{X}_{n\times q}$. Here, $\boldsymbol{Y}_{i\bullet}$ is an $n_i \times p$ matrix and $\boldsymbol{X}_{i\bullet}$ is an $n_i \times q$ matrix. Input the grouping of subjects with $n = \sum_{i=1}^N n_i$.
Begin Iteration: for $d = 1\cdots,p$,
2. **(Estimation of confounding effects using GLMM for root equations)** If $\hat{\pi}_d$ is a root variable indexed by Y_k, estimate the confounding effects $\{h_{ik}\}_{i=1}^N$ by fitting a GLMM on $\boldsymbol{Y}_{\bullet k}$:
$$\mathbb{E}[\boldsymbol{Y}_{ik}|\boldsymbol{X}_{i,\text{in}(k)}, h_{ik}] = \varphi_k(\boldsymbol{X}_{i,\overline{\text{in}}(k)}\boldsymbol{W}_{\overline{\text{in}}(k),k} + h_{ik}\mathbf{1}_{n_i}),$$
where h_{ik} denotes the random effect for ith group. Obtain estimated confounding effects $\{\widehat{h}_{ik}\}$.
3. **(Deconfounding)** If $\hat{\pi}_d$ is a non-root variable indexed by Y_j, compute $(\widehat{\boldsymbol{W}}_{\overline{\text{in}}(j),j}, \widehat{\boldsymbol{U}}_{\overline{\text{an}}(j),j}, \widehat{\boldsymbol{\alpha}}_{\overline{\text{an}}(j),j})$ by fitting a TLP-constrained GLM regression of Y_j in (9). Compute the residuals $\{\widehat{h}_{i'j}\}_{i'=1}^n$ in (10).

Besides the residual inclusion approach proposed in the main paper for deconfounding, we also include a version of incorporating predictor substitution approach in GAMPI, referred to as DPS, in Algorithm 6. Here, $\mathcal{L}(\boldsymbol{W}_{\overline{\text{in}}(j),j}, \boldsymbol{U}_{\overline{\text{an}}(j),j} | \boldsymbol{X}_{\overline{\text{in}}(j)}, \widehat{\boldsymbol{Y}}_{\overline{\text{an}}(j)}) = n^{-1}\sum_{i=1}^{n} \ell(Y_{ij}, \boldsymbol{W}_{\overline{\text{in}}(j),j}^{\top}\boldsymbol{X}_{i,\overline{\text{in}}(j)} + \boldsymbol{U}_{\overline{\text{an}}(j),j}^{\top}\widehat{\boldsymbol{Y}}_{i,\overline{\text{an}}(j)})$, which indicates the endogenous variables are replaced by their predicted values.

Algorithm 6: Peeling algorithm for estimating parent-child relationships via DPS

1. Input $(\overline{\text{an}}(j), \overline{\text{in}}(j))_{j=1}^{p}$ and $\hat{\pi}$ from Algorithm 2. Input data matrix $(Y_{ij}, X_{ij})_{n\times(p+q)} = (\boldsymbol{Y}_{i\bullet}, \boldsymbol{X}_{i\bullet})_{i=1}^{n}$ of primary variables $\boldsymbol{Y}_{n\times p}$ and instruments $\boldsymbol{X}_{n\times q}$.
Begin Iteration: for $d = 1\cdots, p$,
2. **(Predictor substitution for root equation)** If $\hat{\pi}_d$ is a root variable indexed by Y_k, compute $\widehat{\boldsymbol{W}}_{\overline{\text{in}}(k),k}$ by fitting a GLM regression of Y_k on $\boldsymbol{X}$: $\mathbb{E}[Y_k|\boldsymbol{X}] = \varphi_k(\boldsymbol{X}_{\overline{\text{in}}(k)}\boldsymbol{W}_{\overline{\text{in}}(k),k})$.
Impute the predictor: $\widehat{Y}_k = \varphi_k(\boldsymbol{X}_{\overline{\text{in}}(k)}\widehat{\boldsymbol{W}}_{\overline{\text{in}}(k),k})$.
3. **(Predictor substitution for child equation)** If $\hat{\pi}_d$ is a non-root variable indexed by Y_j, compute $(\widehat{\boldsymbol{W}}_{\overline{\text{in}}(j),j}, \widehat{\boldsymbol{U}}_{\overline{\text{an}}(j),j})$ by fitting a TLP-constrained GLM regression of Y_j:

$$
\begin{aligned}
(\widehat{\boldsymbol{W}}_{\overline{\text{in}}(j),j}, \widehat{\boldsymbol{U}}_{\overline{\text{an}}(j),j}) &= \underset{\boldsymbol{W}_{\overline{\text{in}}(j),j}, \boldsymbol{U}_{\overline{\text{an}}(j),j}}{\operatorname{argmin}} \mathcal{L}(\boldsymbol{W}_{\overline{\text{in}}(j),j}, \boldsymbol{U}_{\overline{\text{an}}(j),j} | \boldsymbol{X}_{\overline{\text{in}}(j)}, \widehat{\boldsymbol{Y}}_{\overline{\text{an}}(j)}) \\
\text{subject to} \quad & \sum_{k\in\overline{\text{an}}(j)} I(U_{kj} \neq 0) \leq \overline{K_j}, \quad j = 1, \ldots, p.
\end{aligned}
$$

Impute the predictor: $\widehat{Y}_j = \varphi_j(\boldsymbol{Y}_{\widehat{\text{pa}}(j)}\widehat{\boldsymbol{U}}_{\widehat{\text{pa}}(j),j} + \boldsymbol{X}_{\widehat{\text{in}}(j)}\widehat{\boldsymbol{W}}_{\widehat{\text{in}}(j),j})$.

Appendix C. Additional Simulations

This section provides additional simulations in the paper to demonstrate the necessity of deconfounding in GAMPI.

Ideally, one might suggest estimating the causal relationships directly using the nodewise GLM regression subject to the ℓ_0-constraint in Algorithm 3 of the main paper, without employing the deconfounding approach or adjusting for confounders. That is,

$$
\begin{aligned}
(\widehat{\boldsymbol{W}}_{\overline{\text{in}}(j),j}, \widehat{\boldsymbol{U}}_{\overline{\text{an}}(j),j}) &= \underset{\boldsymbol{W}_{\overline{\text{in}}(j),j}, \boldsymbol{U}_{\overline{\text{an}}(j),j}}{\operatorname{argmin}} \mathcal{L}(\boldsymbol{W}_{\overline{\text{in}}(j),j}, \boldsymbol{U}_{\overline{\text{an}}(j),j} | \boldsymbol{X}_{\overline{\text{in}}(j)}, \boldsymbol{Y}_{\overline{\text{an}}(j)}) \\
\text{subject to} \quad & \sum_{k\in\overline{\text{an}}(j)} I(U_{kj} \neq 0) \leq \overline{K_j}, \quad j = 1, \ldots, p. \qquad (16)
\end{aligned}
$$

Similarly, to select parent-child relationships from the ancestral relationships identified in the first stage, we penalize the number of nonzero elements of $\boldsymbol{U}$. That is, if $\widehat{U}_{kj} \neq 0$, then Y_k is a parent of Y_j, or $Y_k \to Y_j$.

We show in Section C.1 and C.2 that GAMPI adjusting for confounders, performs as well as the above approach (16) when the data is simulated without confounders and outperforms it in the presence of confounders.

C.1 Absence of Confounders

This subsection considers the special case when the data is simulated without confounders for binary outcomes. Recall that the binary data is simulated from the Bernoulli distribution in Section 5.1 of

the main paper with $\boldsymbol{h} = 0$. Table 3 suggests that our deconfounding approach, GAMPI, performs well even when the data is simulated without confounders.

Graph	(p, q, n)	FPR		FDR		F-score		MCC		SHD	
		GAMPI-no deconf	GAMPI	GAMPI-no deconf	GAMPI	GAMPI-no deconf	GAMPI	GAMPI-no deconf	GAMPI	GAMPI-no deconf	GAMPI
Hub	(100,100,300)	0.00 (0.00)	0.00 (0.00)	0.03 (0.00)	0.04 (0.01)	0.98 (0.00)	0.98 (0.00)	0.98 (0.00)	0.98 (0.00)	3.70 (0.52)	4.80 (0.71)
	(100,100,400)	0.00 (0.00)	0.00 (0.00)	0.02 (0.01)	0.02 (0.00)	0.99 (0.00)	0.99 (0.00)	0.99 (0.00)	0.99 (0.00)	2.20 (0.55)	2.30 (0.52)
	(100,100,500)	0.00 (0.00)	0.00 (0.00)	0.02 (0.00)	0.02 (0.00)	0.99 (0.00)	0.99 (0.00)	0.99 (0.00)	0.99 (0.00)	2.10 (0.38)	1.80 (0.29)
Chain	(100,100,300)	0.00 (0.00)	0.00 (0.00)	0.08 (0.01)	0.07 (0.01)	0.82 (0.01)	0.82 (0.01)	0.83 (0.01)	0.83 (0.01)	23.90 (1.45)	23.80 (1.23)
	(100,100,400)	0.00 (0.00)	0.00 (0.00)	0.06 (0.00)	0.06 (0.00)	0.91 (0.01)	0.91 (0.01)	0.91 (0.01)	0.91 (0.01)	13.50 (0.95)	13.20 (0.95)
	(100,100,500)	0.00 (0.00)	0.00 (0.00)	0.05 (0.01)	0.04 (0.01)	0.94 (0.00)	0.95 (0.00)	0.94 (0.00)	0.95 (0.00)	8.30 (0.40)	7.70 (0.45)
Random	(100,100,300)	0.00 (0.00)	0.00 (0.00)	0.10 (0.01)	0.09 (0.01)	0.85 (0.01)	0.85 (0.01)	0.85 (0.01)	0.85 (0.01)	20.10 (1.75)	20.10 (1.67)
	(100,100,400)	0.00 (0.00)	0.00 (0.00)	0.07 (0.01)	0.07 (0.01)	0.93 (0.01)	0.93 (0.01)	0.93 (0.01)	0.93 (0.01)	10.80 (1.75)	10.60 (1.61)
	(100,100,500)	0.00 (0.00)	0.00 (0.00)	0.04 (0.01)	0.04 (0.00)	0.97 (0.00)	0.97 (0.01)	0.97 (0.00)	0.97 (0.01)	4.80 (0.59)	4.50 (0.64)

Table 3: Evaluating GAMPI's reconstruction accuracy for binary outcomes without confounders, utilizing the extended BIC (EBIC) for tuning parameter selection. Evaluation metrics include false positive rate (FPR), false discovery rate (FDR), F-score, Matthews correlation coefficient (MCC), and structural Hamming distance (SHD). "GAMPI-no deconf" method refers to employing the nodewise GLM regression approach without adjusting for confounders based on (16).

C.2 Presence of Confounders

This subsection compares the DRI approach with that without adjusting for confounders under the simulation setting in the presence of confounders. Furthermore, we compare our deconfounding approach, DRI in Algorithm 3 of the main paper, with that via predictor substitution (DPS) in Algorithm 6. In addition to the five metrics in the paper, we compute the estimation error $\|\widehat{\boldsymbol{U}} - \boldsymbol{U}^0\|_F^2$ to evaluate the accuracy of parameter estimation, where $\|\cdot\|_F$ denotes the Frobenius norm.

Graph	(p, q, n)	F-score			$\|\widehat{\boldsymbol{U}} - \boldsymbol{U}^*\|_F^2$		
		GAMPI-no deconf	GAMPI-DRI	GAMPI-DPS	GAMPI-no deconf	GAMPI-DRI	GAMPI-DPS
Hub	(100,100,500)	0.96 (0.01)	0.96 (0.01)	0.91 (0.01)	77.45 (5.28)	58.11 (6.66)	108.04 (12.11)
	(200,200,500)	0.95 (0.01)	0.95 (0.01)	0.89 (0.01)	185.12 (25.01)	140.73 (25.02)	255.65 (24.69)
	(300,300,500)	0.95 (0.01)	0.95 (0.01)	0.90 (0.01)	279.17 (35.82)	200.55 (45.36)	373.97 (47.27)
Chain	(100,100,500)	0.74 (0.01)	0.87 (0.01)	0.87 (0.01)	118.06 (6.37)	74.67 (4.74)	93.06 (5.2)
	(200,200,500)	0.71 (0.01)	0.84 (0.01)	0.84 (0.01)	281.96 (12.01)	189.05 (9.82)	217.29 (10.96)
	(300,300,500)	0.71 (0.01)	0.83 (0.01)	0.84 (0.01)	415.11 (20.26)	294.81 (12.57)	332.47 (13.82)
Random	(100,100,500)	0.71 (0.02)	0.74 (0.02)	0.74 (0.02)	282.58 (15.98)	278.67 (15.95)	321.49 (15.86)
	(200,200,500)	0.64 (0.01)	0.69 (0.01)	0.69 (0.01)	656.68 (30.57)	633.23 (31.49)	711.17 (31.42)
	(300,300,500)	0.59 (0.00)	0.64 (0.00)	0.62 (0.01)	1111.06 (37.05)	1053.11 (32.15)	1174.76 (37.16)

Table 4: Assessing GAMPI's reconstruction accuracy for binary outcomes with confounders, employing the extended BIC (EBIC) for tuning parameter selection. Evaluation metrics include F-score and parameter estimation error in the Frobenius norm. "GAMPI-DPS" employs the predictor substitution (DPS) approach for deconfounding as proposed in Algorithm 6. "GAMPI-DRI" uses the residual inclusion approach proposed in Algorithm 3 of the main paper. In other tables, "GAMPI" refers to the recommended "GAMPI-DRI" approach.

Table 4 suggests that our deconfounding approach outperforms the standard GLM approach (16) without adjusting for confounders in the presence of confounders. Moreover, deconfounding via DRI proposed in Algorithm 3 outperforms that using predictor substitution (DPS) in Algorithm 6 in terms of parameter estimation. Our simulation result indicates that DRI is more suited than DPS for binary or count outcomes, which is concordant with the observation of Terza et al. (2008).

C.3 Presence of Confounders with Replicates

In this subsection, we evaluate the performance of GAMPI using the generalized linear mixed models (GLMMs) for root equations proposed in Algorithm 5 under the simulation setting with repeated measurements. Table 5 suggests that our deconfounding approach using a GLMM outperforms the standard GLM approach, as it better estimates the confounders.

Graph	(p, q, n)	F-score			$\|\widehat{\boldsymbol{U}} - \boldsymbol{U}^*\|_F^2$		
		GAMPI-no deconf	GAMPI	GAMPI-GLMM	GAMPI-no deconf	GAMPI	GAMPI-GLMM
Hub	(100,100,500)	0.94 (0.02)	0.94 (0.02)	0.95 (0.02)	102.96 (24.02)	79.71 (23.14)	61.6 (21.58)
	(200,200,500)	0.92 (0.01)	0.92 (0.01)	0.93 (0.01)	259.2 (24.82)	209.5 (25.42)	160.8 (21.94)
	(300,300,500)	0.91 (0.02)	0.91 (0.02)	0.93 (0.02)	357.33 (74.04)	321.79 (70.8)	251.28 (68.07)
Chain	(100,100,500)	0.75 (0.01)	0.87 (0.01)	0.93 (0.01)	125.64 (6.81)	81.8 (5.5)	65.99 (6.38)
	(200,200,500)	0.71 (0.01)	0.83 (0.01)	0.91 (0.00)	285.35 (12.08)	201.56 (4.04)	155.85 (3.66)
	(300,300,500)	0.69 (0.01)	0.80 (0.01)	0.89 (0.01)	500.19 (20.73)	366.98 (16.24)	287.8 (11.69)

Table 5: Evaluating GAMPI's reconstruction accuracy for binary outcomes in repeated measurements, using the extended BIC (EBIC) for tuning. Evaluation metrics include F-score and parameter estimation error in the Frobenius norm. "GAMPI-GLMM" refers to GAMPI using GLMM for root equations proposed in Algorithm 5.

C.4 Comparison with DAGMA

This subsection compares GAMPI with a recently proposed structure learning method, called DAGMA. DAGMA is designed only for Gaussian or logistic outcomes. Thus, we compare GAMPI with DAGMA for the binary outcomes case. Table 6 suggests that GAMPI continues to outperform DAGMA in most scenarios. Specifically, DAGMA performs equally well in the easy case, namely, the hub graph. However, in challenging situations like the chain and random graphs, GAMPI outperforms DAGMA significantly.

Binary Graph	(p, q, n)	FPR		FDR		F-score		MCC		SHD	
		DAGMA	GAMPI	DAGMA	GAMPI	DAGMA	GAMPI	DAGMA	GAMPI	DAGMA	GAMPI
Hub	(100,100,500)	0.00 (0.00)	0.00 (0.00)	0.04 (0.00)	0.05 (0.01)	0.98 (0.00)	0.96 (0.01)	0.98 (0.00)	0.96 (0.01)	4.20 (0.57)	8.10 (1.46)
	(200,200,500)	0.00 (0.00)	0.00 (0.00)	0.05 (0.01)	0.04 (0.01)	0.97 (0.00)	0.95 (0.01)	0.97 (0.00)	0.95 (0.01)	11.60 (1.60)	20.40 (3.95)
	(300,300,500)	0.00 (0.00)	0.00 (0.00)	0.07 (0.01)	0.04 (0.01)	0.96 (0.00)	0.95 (0.01)	0.96 (0.00)	0.95 (0.01)	24.40 (1.84)	28.20 (7.61)
Chain	(100,100,500)	0.01 (0.00)	0.00 (0.00)	0.43 (0.01)	0.16 (0.02)	0.68 (0.01)	0.87 (0.01)	0.70 (0.01)	0.87 (0.01)	51.70 (2.63)	21.00 (2.72)
	(200,200,500)	0.00 (0.00)	0.00 (0.00)	0.53 (0.01)	0.21 (0.01)	0.60 (0.01)	0.84 (0.01)	0.62 (0.01)	0.84 (0.01)	145.60 (2.84)	52.30 (2.31)
	(300,300,500)	0.00 (0.00)	0.00 (0.00)	0.57 (0.01)	0.22 (0.01)	0.56 (0.01)	0.83 (0.01)	0.59 (0.01)	0.83 (0.01)	247.60 (4.02)	84.30 (5.17)
Random	(100,100,500)	0.01 (0.00)	0.00 (0.00)	0.80 (0.01)	0.14 (0.01)	0.29 (0.01)	0.74 (0.02)	0.30 (0.02)	0.74 (0.02)	141.60 (3.54)	33.90 (1.98)
	(200,200,500)	0.01 (0.00)	0.00 (0.00)	0.82 (0.01)	0.17 (0.01)	0.26 (0.01)	0.69 (0.01)	0.29 (0.01)	0.70 (0.01)	342.90 (5.44)	78.40 (3.25)
	(300,300,500)	0.01 (0.00)	0.00 (0.00)	0.84 (0.01)	0.26 (0.01)	0.24 (0.01)	0.64 (0.00)	0.27 (0.01)	0.65 (0.00)	573.20 (6.88)	144.00 (3.69)

Table 6: Comparing reconstruction accuracy of GAMPI and DAGMA for binary outcomes with confounders, where GAMPI employs the extended BIC (EBIC) for tuning and DAGMA uses the default setting with a tuning parameter value of 0.02.

C.5 Tuning Parameter Selection

This subsection examines the performance of two tuning parameter selection approaches for GAMPI. We use either 5-fold cross-validation or the extended Bayesian information criterion (EBIC) to choose (τ_j, K_j) by minimizing the predictive likelihood or the EBIC criterion. For cross-validation, we adopt the one-standard error rule which is commonly used for the high-dimensional data. We consider the base simulation in the presence of confounders. Table 7 suggests that the EBIC approach outperforms cross-validation in all settings.

Graph	(p, q, n)	F-score		SHD	
		CV	EBIC	CV	EBIC
Hub	(100,100,500)	0.70 (0.03)	0.96 (0.01)	44.30 (3.97)	8.10 (1.46)
	(200,200,500)	0.70 (0.04)	0.95 (0.01)	89.20 (10.25)	20.40 (3.95)
	(300,300,500)	0.74 (0.05)	0.95 (0.01)	120.30 (15.74)	28.20 (7.61)
Chain	(100,100,500)	0.56 (0.03)	0.87 (0.01)	46.20 (1.96)	21.00 (2.72)
	(200,200,500)	0.59 (0.01)	0.84 (0.01)	88.20 (2.10)	52.30 (2.31)
	(300,300,500)	0.56 (0.01)	0.83 (0.01)	137.50 (2.93)	84.30 (5.17)
Random	(100,100,500)	0.55 (0.02)	0.74 (0.02)	46.20 (2.03)	33.90 (1.98)
	(200,200,500)	0.48 (0.02)	0.69 (0.01)	102.50 (4.47)	78.40 (3.25)
	(300,300,500)	0.47 (0.01)	0.64 (0.00)	158.90 (6.32)	144.00 (3.69)

Table 7: Reconstruction accuracy of causal graph of GAMPI for binary outcomes in the presence of confounders, where GAMPI uses cross-validation (CV) or the extended BIC (EBIC) for tuning parameter selection. Evaluation metrics include F-score and SHD.

C.6 Causal Graph Selection Consistency

In this subsection, we verify our theoretical statements and demonstrate the consistency of the proposed method, as proved in Theorem 5. Following Ravikumar et al. (2011), we evaluate the performance of the method in terms of the probability of correct causal graph selection. Figure 4 plots the probability of correct causal graph recovery against the sample size n, with varying number of nodes p. The probability of correct causal graph selection is calculated as the proportion of the $B = 50$ trials in which the proposed GAMPI recovers the directed edge sets exactly. We consider the hub and chain graphs for Poisson primary variables with the same setup as the base simulation in Table 2 except varying the sample size n and number of nodes p. For each curve, the probability of success starts at zero, and converges to one as the sample size increases, suggesting the causal graph selection consistency of our proposed method. Figure 4 shows that the proposed method performs well when the sample size n is large or the number of nodes p (graph size) is small, aligning with the theoretical results stated in Theorem 5. Figure 4 also suggests that a larger graph size requires a larger sample size for exact graph recovery, so that the curve for $p = 300$ is shifted to the right compared with the curve for $p = 100$.

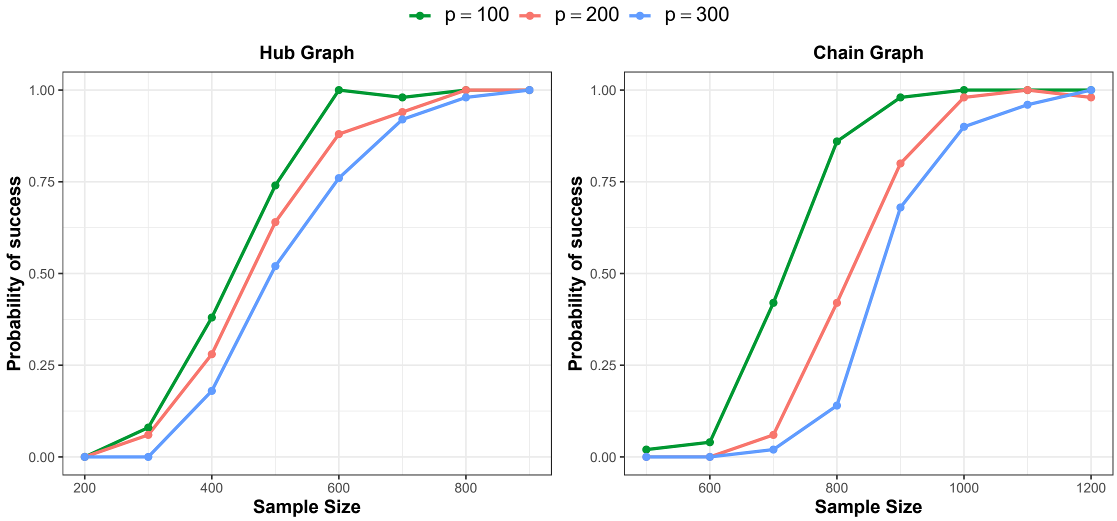

Figure 4: Simulation results of graph selection consistency for Poisson hub and chain graphs with varying number of nodes p; plots of probability of correct directed edge-set recovery versus the sample size n. Each point corresponds to the average over 50 trials.

C.7 Correlated Instrumental Variables

In this subsection, we evaluate the performance of the proposed method when the instrumental variables $\boldsymbol{X}$ are correlated. We consider the same setup for binary primary variables as the base simulation in Table 2 except that $\boldsymbol{X}$ is Gaussian with autoregressive covariance $\boldsymbol{X} \sim N(\mathbf{0}, \mathbf{\Sigma}_X)$ where $(\mathbf{\Sigma}_X)_{ij} = \rho^{|i-j|}$ and $\rho = 0.5$. Figure 5 shows that our method still performs well in the case where the instrumental variables are correlated. We compare our proposed method with DAGMA as it is shown to outperform NOTEARS in Appendix C.4.

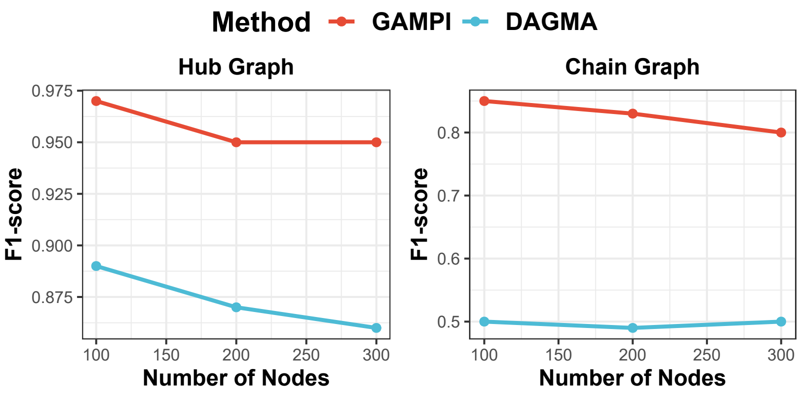

Figure 5: Simulation results when the instrumental variables $\boldsymbol{X}$ are correlated.

C.8 Comparison with Linear Deconfounding Structure Learning Algorithm

This subsection compares GAMPI with a recently proposed linear deconfounding algorithm, called GrIVET (Chen et al. 2023). Compared with Chen et al. (2023), we use the generalized linear models to account for different distributions of the outcome variables, which enhance model interpretation. Moreover, in contrast to the imputation-based approach by Chen et al. (2023), we propose a residual-inclusion-based deconfounding algorithm to address confounders, which has been shown to be more suitable for nonlinear outcomes. Going beyond, we present novel theoretical analysis including the fidelity model and consistency of residual inclusion for the GLMs. We compare the two methods for the Poisson outcomes case as in Table 2. Figure 6 suggests that our proposed method outperforms the existing linear deconfounding algorithm, demonstrating the advantage of the proposed method for handling non-Gaussian outcomes.

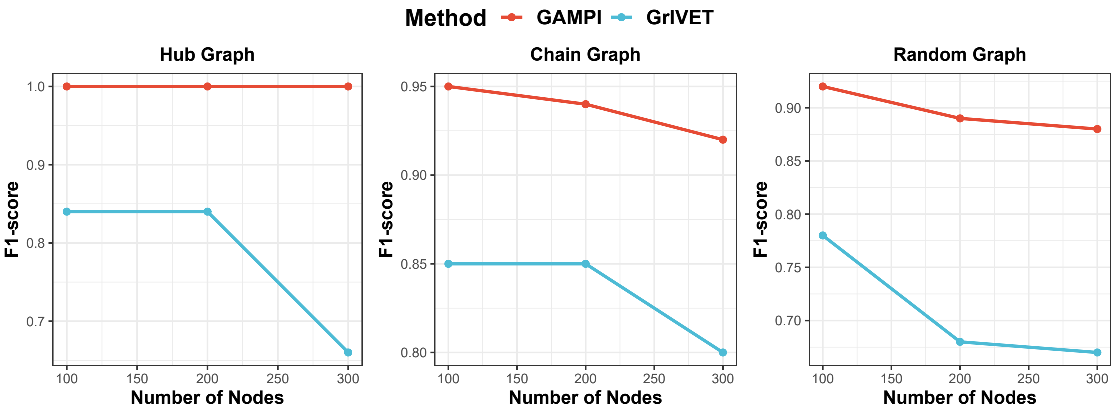

Figure 6: Simulation results of comparison with linear deconfounding algorithm, GrIVET.

Appendix D. Technical Proofs

D.1 Proof of Proposition 1

We prove the proposition in two steps. First, we show that the topological order and the corresponding DAG are identifiable. Next, we show that the model parameters are identifiable given the graph. Assume that two structural equation models as in equation (2), defined by $\boldsymbol{\theta} = (\boldsymbol{U}, \boldsymbol{W})$ and $\tilde{\boldsymbol{\theta}} = (\widetilde{\boldsymbol{U}}, \widetilde{\boldsymbol{W}})$, induce the same distribution of $(\boldsymbol{Y}, \boldsymbol{X})$. We will show that $\boldsymbol{\theta} = \tilde{\boldsymbol{\theta}}$.

Identifying G. Let $G(\boldsymbol{\theta})$ and $G(\tilde{\boldsymbol{\theta}})$ be the DAGs corresponding to $\boldsymbol{\theta}$ and $\tilde{\boldsymbol{\theta}}$. First, we show that the topological order of $Y_1, \cdots, Y_p$ is identifiable. For $G(\boldsymbol{\theta})$, assume, without loss of generality, that Y_1 is a leaf node in $G(\boldsymbol{\theta})$. By Assumption 1(B), there exists a valid instrument, say X_1, that intervenes on Y_1. By Assumptions 1(A) and (ii),

$$\operatorname{Cov}\left(Y_1, X_1 \mid \boldsymbol{Y}_S, \boldsymbol{X}_{\{2,\ldots,q\}}\right) \neq 0, \qquad \text{for any } S \subseteq \{2, \ldots, p\}, \tag{17}$$

$$\operatorname{Cov}\left(Y_j, X_1 \mid \boldsymbol{X}_{\{2,\ldots,q\}}\right) = 0, \qquad j = 2, \ldots, p. \tag{18}$$

Hence, (17) implies that $X_1 \to Y_1$ in $G(\tilde{\boldsymbol{\theta}})$. Now suppose Y_1 is not a leaf node in $G(\tilde{\boldsymbol{\theta}})$ and there exists Y_2 such that $Y_1 \to Y_2$. Then, $\operatorname{Cov}\left(Y_2, X_1 \mid \boldsymbol{X}_{\{2,\ldots,q\}}\right) = 0$ by (18) but $X_1 \to Y_1$ and $Y_1 \to Y_2$, which contradicts Assumption 1(A). Therefore, if Y_1 is a leaf node in $G(\boldsymbol{\theta})$, then Y_1 must also be a leaf node in $G(\tilde{\boldsymbol{\theta}})$. Therefore, we can identify the leaf nodes $\boldsymbol{Y}_{\mathcal{L}_1}$ in the graph. Further, following Li et al. (2023), the parents and instruments in $G(\boldsymbol{\theta})$ and $G(\tilde{\boldsymbol{\theta}})$ of Y_1 can be identified by:

$$\mathbb{E}(Y_1 \mid \boldsymbol{Y}_{-1}, \boldsymbol{X}, \boldsymbol{h}) = \mathbb{E}(Y_1 \mid \boldsymbol{Y}_{\mathrm{pa}_{G(\boldsymbol{\theta})}(1)}, \boldsymbol{X}, h_1) = \mathbb{E}(Y_1 \mid \boldsymbol{Y}_{\mathrm{pa}_{G(\boldsymbol{\theta})}(1)}, \boldsymbol{X}_{\mathrm{in}_{G(\boldsymbol{\theta})}(1)}, h_1), \tag{19}$$

$$\mathbb{E}(Y_1 \mid \boldsymbol{Y}_{-1}, \boldsymbol{X}, \boldsymbol{h}) = \mathbb{E}(Y_1 \mid \boldsymbol{Y}_{\mathrm{pa}_{G(\tilde{\boldsymbol{\theta}})}(1)}, \boldsymbol{X}, h_1) = \mathbb{E}(Y_1 \mid \boldsymbol{Y}_{\mathrm{pa}_{G(\tilde{\boldsymbol{\theta}})}(1)}, \boldsymbol{X}_{\mathrm{in}_{G(\tilde{\boldsymbol{\theta}})}(1)}, h_1), \tag{20}$$

where $\mathrm{pa}_{G(\boldsymbol{\theta})}(1)$ refers to the parent variables of Y_1 in $G(\boldsymbol{\theta})$ and $\mathrm{in}_{G(\boldsymbol{\theta})}(1)$ refers to the instrumental variables of Y_1 in $G(\boldsymbol{\theta})$; $\mathrm{pa}_{G(\tilde{\boldsymbol{\theta}})}(1)$ and $\mathrm{in}_{G(\tilde{\boldsymbol{\theta}})}(1)$ are similarly defined for $G(\tilde{\boldsymbol{\theta}})$. We have $\mathrm{pa}_{G(\boldsymbol{\theta})}(1) = \mathrm{pa}_{G(\tilde{\boldsymbol{\theta}})}(1)$ and $\mathrm{in}_{G(\boldsymbol{\theta})}(1) = \mathrm{in}_{G(\tilde{\boldsymbol{\theta}})}(1)$. To see this, if there exists Y_k such that $k \in \mathrm{pa}_{G(\boldsymbol{\theta})}(1)$ and $k \notin \mathrm{pa}_{G(\tilde{\boldsymbol{\theta}})}(1)$, we have $\operatorname{Cov}\left(Y_1, Y_k \mid \boldsymbol{Y}_{\{2,\ldots,p\}\setminus k}, \boldsymbol{X}, h_1\right) \neq 0$ by (19) and $\operatorname{Cov}\left(Y_1, Y_k \mid \boldsymbol{Y}_{\{2,\ldots,p\}\setminus k}, \boldsymbol{X}, h_1\right) = 0$ by (20), leading to a contradiction, and we conclude that $\mathrm{pa}_{G(\boldsymbol{\theta})}(1) = \mathrm{pa}_{G(\tilde{\boldsymbol{\theta}})}(1)$. Similarly, we have $\mathrm{in}_{G(\boldsymbol{\theta})}(1) = \mathrm{in}_{G(\tilde{\boldsymbol{\theta}})}(1)$. Therefore, the leaf node Y_1 has the same parents and instruments in $G(\boldsymbol{\theta})$ and $G(\tilde{\boldsymbol{\theta}})$.

Toward this end, we have identified the leaf nodes $\boldsymbol{Y}_{\mathcal{L}_1}$ along with their parents and instruments respectively. Next, after removing the leaf nodes $\boldsymbol{Y}_{\mathcal{L}_1}$, we apply the same argument and identify the leaf variables $\boldsymbol{Y}_{\mathcal{L}_2}$ in the sub-graph. We proceed until all the variables are removed, leading to $G(\boldsymbol{\theta}) = G(\tilde{\boldsymbol{\theta}})$ and $\mathrm{in}(j) = \widetilde{\mathrm{in}}(j)$, $\forall j$. In this way, the graph G and the topological order can be identified.

Identifying $\boldsymbol{\theta} = (\boldsymbol{U}, \boldsymbol{W})$ **given** G. Second, we show that $\boldsymbol{\theta} = \tilde{\boldsymbol{\theta}}$. Recall that for the jth equation, $\psi_j(\mathbb{E}\left(Y_j | \boldsymbol{Y}_{\mathrm{pa}(j)}, \boldsymbol{X}, h_j\right)) = \boldsymbol{u}_j^\top \boldsymbol{Y}_{\mathrm{pa}(j)} + \boldsymbol{w}_j^\top \boldsymbol{X}_{\mathrm{in}(j)} + h_j$, $j = 1, \cdots, p$. Let Y_k be a parent of Y_j. We can rewrite the above equation as $\psi_j(\mathbb{E}\left(Y_j | \boldsymbol{Y}_{\mathrm{pa}(j)}, \boldsymbol{X}, h_j\right)) = U_{kj} \boldsymbol{Y}_k + \boldsymbol{U}_{\mathrm{pa}(j)\setminus k, j} \boldsymbol{Y}_{\mathrm{pa}(j)\setminus k} + \boldsymbol{w}_j^\top \boldsymbol{X}_{\mathrm{in}(j)} + h_j$. Similarly, for the kth equation, $\mathbb{E}\left(\boldsymbol{Y}_k | \boldsymbol{Y}_{\mathrm{pa}(k)}, \boldsymbol{X}, h_k\right) = \psi_k^{-1}(\boldsymbol{u}_k^\top \boldsymbol{Y}_{\mathrm{pa}(k)} + \boldsymbol{w}_k^\top \boldsymbol{X}_{\mathrm{in}(k)} + h_k)$. Therefore,

$$\begin{aligned} &\mathbb{E}\left(\psi_j(\mathbb{E}\left(Y_j | \boldsymbol{Y}_{\mathrm{pa}(j)}, \boldsymbol{X}, h_j\right)) - \boldsymbol{U}_{\mathrm{pa}(j)\setminus k, j} \boldsymbol{Y}_{\mathrm{pa}(j)\setminus k} | \boldsymbol{Y}_{\mathrm{pa}(k)}, \boldsymbol{X}, h_k\right) \\ &= U_{kj} \psi_k^{-1}(\boldsymbol{u}_k^\top \boldsymbol{Y}_{\mathrm{pa}(k)} + \boldsymbol{w}_k^\top \boldsymbol{X}_{\mathrm{in}(k)} + h_k) + \boldsymbol{W}_{\mathrm{in}(j), j}^\top \boldsymbol{X}_{\mathrm{in}(j)} + \mathbb{E}\left(h_j | \boldsymbol{Y}_{\mathrm{pa}(k)}, \boldsymbol{X}, h_k\right). \end{aligned} \tag{21}$$

Note that the left-hand side is not equal to $\psi_j(\mathbb{E}\left(Y_j | \boldsymbol{Y}_{\mathrm{pa}(k)}, \boldsymbol{X}, h_j\right))$ but still characterizes a proper conditional distribution. We next prove the identifiability of $\boldsymbol{\theta} = (\boldsymbol{U}, \boldsymbol{W})$ by induction. Suppose $\boldsymbol{u}_k = \boldsymbol{U}_{\bullet k}$ and $\boldsymbol{w}_k = \boldsymbol{W}_{\bullet k}$ are identified. We will show that U_{kj} and $\boldsymbol{W}_{\mathrm{in}(j), j}$ are identifiable and

therefore $\boldsymbol{\theta} = (\boldsymbol{U}, \boldsymbol{W})$ is also identifiable by induction on the topological depth. If there exist $\widetilde{U}_{kj} \neq U_{kj}$ and $\widetilde{\boldsymbol{W}}_{\text{in}(j),j} \neq \boldsymbol{W}_{\text{in}(j),j}$ which render the same conditional distribution (21) in that $U_{kj}\psi_k^{-1}(\boldsymbol{u}_k^\top \boldsymbol{Y}_{\text{pa}(k)} + \boldsymbol{w}_k^\top \boldsymbol{X}_{\text{in}(k)} + h_k) + \boldsymbol{W}_{\text{in}(j),j}^\top \boldsymbol{X}_{\text{in}(j)} + \mathbb{E}_{\boldsymbol{\theta}}\left(h_j | \boldsymbol{Y}_{\text{pa}(k)}, \boldsymbol{X}, h_k\right) = \widetilde{U}_{kj}\psi_k^{-1}(\boldsymbol{u}_k^\top \boldsymbol{Y}_{\text{pa}(k)} + \boldsymbol{w}_k^\top \boldsymbol{X}_{\text{in}(k)} + h_k) + \widetilde{\boldsymbol{W}}_{\text{in}(j),j}^\top \boldsymbol{X}_{\text{in}(j)} + \mathbb{E}_{\tilde{\boldsymbol{\theta}}}\left(h_j | \boldsymbol{Y}_{\text{pa}(k)}, \boldsymbol{X}, h_k\right)$. Rearranging terms yields that

$$\begin{aligned} &U_{kj}\psi_k^{-1}(\boldsymbol{u}_k^\top \boldsymbol{Y}_{\text{pa}(k)} + \boldsymbol{w}_k^\top \boldsymbol{X}_{\text{in}(k)} + h_k) - \widetilde{U}_{kj}\psi_k^{-1}(\boldsymbol{u}_k^\top \boldsymbol{Y}_{\text{pa}(k)} + \boldsymbol{w}_k^\top \boldsymbol{X}_{\text{in}(k)} + h_k) \\ &= \widetilde{\boldsymbol{W}}_{\text{in}(j),j}^\top \boldsymbol{X}_{\text{in}(j)} - \boldsymbol{W}_{\text{in}(j),j}^\top \boldsymbol{X}_{\text{in}(j)} + \mathbb{E}_{\tilde{\boldsymbol{\theta}}}\left(h_j | \boldsymbol{Y}_{\text{pa}(k)}, \boldsymbol{X}, h_k\right) - \mathbb{E}_{\boldsymbol{\theta}}\left(h_j | \boldsymbol{Y}_{\text{pa}(k)}, \boldsymbol{X}, h_k\right). \end{aligned} \tag{22}$$

If $\psi_k^{-1}(\cdot)$ is a non-linear function, then the left-hand side cannot be linearly represented by the right-hand side linear function of $\boldsymbol{X}_{\text{in}(j)}$. To see this, note that there exists an instrumental variable X_l for Y_k, $l \in \text{in}(k)$. In addition, by Assumption 2, $\mathbb{E}_{\boldsymbol{\theta}}\left(h_j | \boldsymbol{Y}_{\text{pa}(k)}, \boldsymbol{X}, h_k\right) = \mathbb{E}_{\boldsymbol{\theta}}\left(h_j | \boldsymbol{h}_{\text{pa}(k)}, \boldsymbol{X}, h_k\right) = \sum_{m \in \{k \cup \text{pa}(k)\}} \alpha_m h_m$, leading to $\mathbb{E}_{\tilde{\boldsymbol{\theta}}}\left(h_j | \boldsymbol{Y}_{\text{pa}(k)}, \boldsymbol{X}, h_k\right) - \mathbb{E}_{\boldsymbol{\theta}}\left(h_j | \boldsymbol{Y}_{\text{pa}(k)}, \boldsymbol{X}, h_k\right) = \sum_m (\tilde{\alpha}_m - \alpha_m) h_m$. Taking the second derivative of (22) with respect to X_l and then h_k yields that

$$(U_{kj} - \widetilde{U}_{kj}) W_{lk}^2 \cdot (\psi_k^{-1})'''(\boldsymbol{u}_k^\top \boldsymbol{Y}_{\text{pa}(k)} + \boldsymbol{w}_k^\top \boldsymbol{X}_{\text{in}(k)} + h_k) = 0,$$

where we use the property that the second derivative of a linear function is zero. This implies $U_{kj} = \widetilde{U}_{kj}$ and therefore U_{kj} is identifiable. Further, plugging $U_{kj} = \widetilde{U}_{kj}$ into (22) yields $\widetilde{\boldsymbol{W}}_{\text{in}(j),j} = \boldsymbol{W}_{\text{in}(j),j}$. Note the statement still holds in the absence of the confounders $\boldsymbol{h}$ by taking the second derivative of (22) with respect to X_l.

If $\psi_k^{-1}(\cdot)$ is a linear function, then the same conclusion holds under the majority rule that the number of valid IVs for Y_k exceeds 50% of its total number of IVs. To see this, let $\text{in}_*(k)$ and $\widetilde{\text{in}}_*(k)$ denote the valid IVs of Y_k in $G(\boldsymbol{\theta})$ and $G(\tilde{\boldsymbol{\theta}})$ respectively. Given a linear ψ_k^{-1}, (22) can be written as

$$\begin{aligned} U_{kj}\boldsymbol{W}_{\text{in}(k),k}^\top \boldsymbol{X}_{\text{in}(k)} - \widetilde{U}_{kj}\boldsymbol{W}_{\text{in}(k),k}^\top \boldsymbol{X}_{\text{in}(k)} = (\widetilde{U}_{kj} - U_{kj})(\boldsymbol{u}_k^\top \boldsymbol{Y}_{\text{pa}(k)} + h_k) + \widetilde{\boldsymbol{W}}_{\text{in}(j),j}^\top \boldsymbol{X}_{\text{in}(j)} - \boldsymbol{W}_{\text{in}(j),j}^\top \boldsymbol{X}_{\text{in}(j)} \\ + \mathbb{E}_{\tilde{\boldsymbol{\theta}}}\left(h_j | \boldsymbol{Y}_{\text{pa}(k)}, \boldsymbol{X}, h_k\right) - \mathbb{E}_{\boldsymbol{\theta}}\left(h_j | \boldsymbol{Y}_{\text{pa}(k)}, \boldsymbol{X}, h_k\right). \end{aligned}$$

Let I denote the right-hand side term, which is not a function of $\boldsymbol{X}_{\text{in}_*(k)}$ or $\boldsymbol{X}_{\widetilde{\text{in}}_*(k)}$. Rearranging terms yields that

$$U_{kj}\boldsymbol{W}_{\text{in}_*(k),k}^\top \boldsymbol{X}_{\text{in}_*(k)} - \widetilde{U}_{kj}\boldsymbol{W}_{\widetilde{\text{in}}_*(k),k}^\top \boldsymbol{X}_{\widetilde{\text{in}}_*(k)} = \widetilde{U}_{kj}\boldsymbol{W}_{\text{in}(k)\setminus\widetilde{\text{in}}_*(k),k}^\top \boldsymbol{X}_{\text{in}(k)\setminus\widetilde{\text{in}}_*(k)} - U_{kj}\boldsymbol{W}_{\text{in}(k)\setminus\text{in}_*(k),k}^\top \boldsymbol{X}_{\text{in}(k)\setminus\text{in}_*(k)} + I.$$

By the majority rule, $|\text{in}_*(k)| > |\text{in}(k)|/2$ and $|\widetilde{\text{in}}_*(k)| > |\text{in}(k)|/2$. Hence, there must exist some valid IV, $l \in \text{in}_*(k) \cap \widetilde{\text{in}}_*(k)$, such that $(U_{kj} - \widetilde{U}_{kj}) W_{lk} X_l$ cannot be linearly represented by $\boldsymbol{X}_{\text{in}(k)\setminus(\text{in}_*(k)\cap\widetilde{\text{in}}_*(k))}$ or $\boldsymbol{X}_{\text{in}(k)^c}$. Again, we have $\widetilde{U}_{kj} = U_{kj}$, $\widetilde{\boldsymbol{W}}_{\text{in}(j),j} = \boldsymbol{W}_{\text{in}(j),j}$. This completes the proof.

Before proving Proposition 2 and Proposition 3, we first introduce Lemma 6 which investigates the marginal distribution defined by the true model $\mathbb{P}(Y_j | \boldsymbol{X})$ and instruments.

Lemma 6 *For a valid instrument X_l, if the marginal distribution under the true model $\mathbb{P}(Y_j | \boldsymbol{X})$ satisfies: $\frac{\partial}{\partial X_l}\mathbb{P}(Y_j | \boldsymbol{X}) \neq 0$, then X_l intervenes on Y_j or an ancestor of Y_j.*

Proof of Lemma 6. Note that the marginal distribution can be written as

$$f(Y_j | \boldsymbol{X}) = \iint f(Y_j | \boldsymbol{Y}_{\text{pa}(j)}, \boldsymbol{X}_{\text{in}(j)}, h_j) f(\boldsymbol{Y}_{\text{pa}(j)} | \boldsymbol{X}) f(h_j)\, d\boldsymbol{Y}_{\text{pa}(j)}\, dh_j. \tag{23}$$

If $\frac{\partial}{\partial X_l}\mathbb{P}(Y_j | \boldsymbol{X}) \neq 0$, or equivalently, $\frac{\partial}{\partial X_l} f(Y_j | \boldsymbol{X}) \neq 0$, then, by the product rule and Assumption 1(C), i) $\frac{\partial}{\partial X_l} f(Y_j | \boldsymbol{Y}_{\text{pa}(j)}, \boldsymbol{X}_{\text{in}(j)}, h_j) \neq 0$, or ii) $\frac{\partial}{\partial X_l} f(\boldsymbol{Y}_{\text{pa}(j)} | \boldsymbol{X}) \neq 0$. Note $f(Y_j | \boldsymbol{Y}_{\text{pa}(j)}, \boldsymbol{X}_{\text{in}(j)}, h_j) =$

$\exp(Y_j(\boldsymbol{U}_{\mathrm{pa}(j),j}^\top \boldsymbol{Y}_{\mathrm{pa}(j)} + \boldsymbol{W}_{\mathrm{in}(j),j}^\top \boldsymbol{X}_{\mathrm{in}(j)} + h_j) - A_j(\boldsymbol{U}_{\mathrm{pa}(j),j}^\top \boldsymbol{Y}_{\mathrm{pa}(j)} + \boldsymbol{W}_{\mathrm{in}(j),j}^\top \boldsymbol{X}_{\mathrm{in}(j)} + h_j))$ under (2). By the chain rule, $\frac{\partial f(Y_j|\boldsymbol{Y}_{\mathrm{pa}(j)}, \boldsymbol{X}_{\mathrm{in}(j)}, h_j)}{\partial X_l} = f(Y_j|\boldsymbol{Y}_{\mathrm{pa}(j)}, \boldsymbol{X}_{\mathrm{in}(j)}, h_j)(Y_j - \varphi_j(\boldsymbol{U}_{\mathrm{pa}(j),j}^\top \boldsymbol{Y}_{\mathrm{pa}(j)} + \boldsymbol{W}_{\mathrm{in}(j),j}^\top \boldsymbol{X}_{\mathrm{in}(j)} + h_j))W_{lj}$. Therefore, condition i) implies that $W_{lj} \neq 0$ and $l \in \mathrm{in}(j)$. Condition ii) implies that there exists an $m \in \mathrm{pa}(j)$ such that $\frac{\partial}{\partial X_l} f(Y_m|\boldsymbol{X}) \neq 0$. Similarly, this implies $l \in \mathrm{in}(m), m \in \mathrm{pa}(j)$, or there exists an $r \in \mathrm{pa}(m)$ such that $\frac{\partial}{\partial X_l} f(Y_r|\boldsymbol{X}) \neq 0$. By induction, we conclude that if $\frac{\partial}{\partial X_l}\mathbb{P}(Y_j|\boldsymbol{X}) \neq 0$, then (i) there exists an $l \in \mathrm{in}(j)$ such that $W_{lj} \neq 0$, or (ii) there exists an $k \in \mathrm{an}(j)$ and $l \in \mathrm{in}(k)$ such that $W_{lk} \neq 0$. Hence, X_l intervenes on Y_j or an ancestor of Y_j.

Remark: By the proof of Lemma 6 and (23), for a general IV (valid or non-valid), if $\frac{\partial}{\partial X_l}\mathbb{P}(Y_j|\boldsymbol{X}) \neq 0$, then i) $\frac{\partial}{\partial X_l} f(Y_j|\boldsymbol{Y}_{\mathrm{pa}(j)}, \boldsymbol{X}_{\mathrm{in}(j)}, h_j) \neq 0$, or ii) $\frac{\partial}{\partial X_l} f(\boldsymbol{Y}_{\mathrm{pa}(j)}|\boldsymbol{X}) \neq 0$, or iii) $\frac{\partial}{\partial X_l} f(h_j) \neq 0$. This suggests: i) X_l intervenes on Y_j or an ancestor of Y_j, or ii) X_l is correlated with h_j or $h_{\mathrm{an}(j)}$.

D.2 Proof of Proposition 2

Recall that $S_j = \{l : V_{lj} \neq 0\}$ for the fidelity model (3) and $\widetilde{S}_j = \{l : \frac{\partial \mathbb{P}(Y_j|\boldsymbol{X})}{\partial X_l} \neq 0\}$ for the true model $\mathbb{P}(Y_j|\boldsymbol{X})$. Next, we will show that $\{l : V_{lj} \neq 0\} = \widetilde{S}_j$, implying that the fidelity model $\mathbb{P}^*(Y_j|\boldsymbol{X})$ and the marginal model $\mathbb{P}(Y_j|\boldsymbol{X})$ have the same support.

For any $l \in \widetilde{S}_j$, $\frac{\partial \mathbb{P}(Y_j|\boldsymbol{X})}{\partial X_l} \neq 0$. By Lemma 6 and the remark, i) X_l intervenes on Y_j or an ancestor of Y_j, or ii) X_l is correlated with h_j or $h_{\mathrm{an}(j)}$. Case i) suggests that $l \in \{\mathrm{in}(j)\} \cup \mathrm{in}(\mathrm{an}(j))$. Hence, there exists a path in the graph from X_l to Y_j: $X_l \to Y_k \to \cdots \to Y_j$. By the local faithfulness in Assumption 1(A), $\mathrm{Cov}(X_l, Y_j) \neq 0$. This implies that $V_{lj} \neq 0$ in the fidelity model. Otherwise, suppose $V_{lj} = 0$. By (3), $\mathbb{E}(Y_j|X_l, \boldsymbol{X}_{-l}) = \mathbb{E}(Y_j|\boldsymbol{X}_{-l})$, implying that $\mathbb{P}(Y_j|X_l, \boldsymbol{X}_{-l}) = \mathbb{P}(Y_j|\boldsymbol{X}_{-l})$ and thus $\mathrm{Cov}(X_l, Y_j) = 0$ by the definition of conditional independence, which contradicts $\mathrm{Cov}(X_l, Y_j) \neq 0$. Hence, $V_{lj} \neq 0$ in the fidelity model. For case ii), $\mathrm{Cov}(X_l, h_j) \neq 0$ or $\mathrm{Cov}(X_l, h_{\mathrm{an}(j)}) \neq 0$ implies $\mathrm{Cov}(X_l, Y_j) \neq 0$. Following the same argument as in case i), we obtain $V_{lj} \neq 0$. Combining the two cases, $V_{lj} \neq 0$ in the fidelity model or $l \in S_j$, implying $\widetilde{S}_j \subset S_j$.

On the other hand, for any $l \in S_j$, $V_{lj} \neq 0$. Then, $\mathbb{E}[Y_j|X_l, \boldsymbol{X}_{-l}] \neq \mathbb{E}[Y_j|\boldsymbol{X}_{-l}]$. Now, suppose $\frac{\partial \mathbb{P}(Y_j|\boldsymbol{X})}{\partial X_l} = 0$. Then, as in (23), there does not exist a path in the graph from X_l to Y_j. Moreover, we have $\frac{\partial}{\partial X_l} f(h_j) = 0$ and $\frac{\partial}{\partial X_l} f(h_{\mathrm{an}(j)}) = 0$, implying $\mathrm{Cov}(X_l, h_j) = 0$ and $\mathrm{Cov}(X_l, h_{\mathrm{an}(j)}) = 0$. Thus, $\mathrm{Cov}(X_l, Y_j) = 0$, which contradicts $\mathbb{E}[Y_j|X_l, \boldsymbol{X}_{-l}] \neq \mathbb{E}[Y_j|\boldsymbol{X}_{-l}]$. Hence, $l \in \widetilde{S}_j$ and thus $S_j \subset \widetilde{S}_j$. This establishes that $S_j = \widetilde{S}_j$.

D.3 Proof of Proposition 3

If $V_{lj} \neq 0$, then $\frac{\partial}{\partial X_l}\mathbb{P}(Y_j|\boldsymbol{X}) \neq 0$ by Proposition 2. For a valid instrument X_l, by Lemma 6, X_l intervenes on Y_j or an ancestor of Y_j.

Moreover, following Li et al. (2023), for a leaf node Y_j, there exists a valid instrument $X_l \to Y_j$ by Assumption 1(B). If there exists $j' \neq j$ such that $V_{lj'} \neq 0$, then Y_j must be an ancestor of $Y_{j'}$, which contradicts the fact that Y_j is a leaf node. On the other hand, suppose $V_{lj} \neq 0$ and $V_{lj'} = 0$, $\forall j' \neq j$. If Y_j is not a leaf node, then there exists a $Y_{j'}$ such that Y_j is a parent of $Y_{j'}$. This implies $\frac{\partial}{\partial X_l} f(Y_{j'}|\boldsymbol{X}) \neq 0$ and thus $V_{lj'} \neq 0$, which contradicts $\|V_{l\bullet}\|_0 = 1$.

D.4 Proof of Theorem 4

Let $S_j^0 = \{l : V_{lj}^0 \neq 0\}$ and $S_j^{[t]} = \{l : |\widetilde{V}_{lj}^{[t]}| \geq \tau_j\}$ be the indices of the true and estimated non-zero elements of the jth columns $\widehat{\boldsymbol{V}}_{\bullet j}^0$ and $\widetilde{\boldsymbol{V}}_{\bullet j}^{[t]}$ at the t-th iteration of Algorithm 1, respectively. Let the

corresponding false negative and positive sets be $\mathrm{FN}_j^{[t]} = S_j^0 \backslash S_j^{[t]}$ and $\mathrm{FP}_j^{[t]} = S_j^{[t]} \backslash S_j^0$ at iteration t. Let an event $\mathcal{E}_j = \big\{\|\boldsymbol{X}^\top \widehat{\boldsymbol{\xi}}_j/n\|_\infty \le 0.5\gamma_j\tau_j\big\} \cap \big\{\|\widehat{\boldsymbol{V}}_{\bullet j}^0 - \boldsymbol{V}_{\bullet j}^0\|_\infty \le 0.5\tau_j\big\}$, where $\widehat{\boldsymbol{\xi}}_j = \boldsymbol{Y}_j - \varphi_j(\boldsymbol{X}\widehat{\boldsymbol{V}}_{\bullet j}^0)$ is the residual of the oracle MLE $\widehat{\boldsymbol{V}}_{\bullet j}^0$ for the GLM, with the support $\{l : \widehat{V}_{lj}^0 \neq 0\} = S_j^0$. Consider the data matrix $(\boldsymbol{X}_{n\times q}, \boldsymbol{Y}_{n\times p})$ and $\boldsymbol{Y}_j$ refers to the j-th column of $\boldsymbol{Y}$, that is, an $n\times 1$ vector.

Our proof consists of three steps. In **Step 1**, we show by induction that if $\big|S_j^0 \cup S_j^{[t-1]}\big| \le 2K_j^0$ on $\mathcal{E}_j$, then $|S_j^0 \cup S_j^{[t]}| \le 2K_j^0$, $t = 1, \ldots$, so that Assumption 4 applies. Recall that $K_j^0 = \|\boldsymbol{V}_{\bullet j}^0\|_0 = |S_j^0|$. In **Step 2**, we estimate the number of iterations to termination T. Particularly, we prove that $|\mathrm{FP}_j^{[t]}| + |\mathrm{FN}_j^{[t]}| < 1$ or $|\mathrm{FP}_j^{[t]}| = |\mathrm{FN}_j^{[t]}| = 0$ and thus $S_j^{[t]} = S_j^0$, for $t \ge T$. In **Step 3**, we bound $\mathbb{P}(\mathcal{E}_j)$ and show that $1 - \mathbb{P}\big(\cup_{j=1}^p \mathcal{E}_j^c\big)$ has a high probability tending to one as $n \to \infty$.

Step 1: Suppose $|S_j^0 \cup S_j^{[t-1]}| \le 2K_j^0$ on $\mathcal{E}_j$. By the Taylor's expansion of the gradient $\nabla\mathcal{L}(\widetilde{\boldsymbol{V}}_{\bullet j}^{[t]})$ at $\widehat{\boldsymbol{V}}_{\bullet j}^0$,

$$\nabla\mathcal{L}(\widetilde{\boldsymbol{V}}_{\bullet j}^{[t]}) = \nabla\mathcal{L}(\widehat{\boldsymbol{V}}_{\bullet j}^0) + \nabla^2\mathcal{L}(\overline{\boldsymbol{V}}_{\bullet j})\big(\widetilde{\boldsymbol{V}}_{\bullet j}^{[t]} - \widehat{\boldsymbol{V}}_{\bullet j}^0\big), \tag{24}$$

where $\overline{\boldsymbol{V}}_{\bullet j}$ is a vector of intermediate values on the line between $\widehat{\boldsymbol{V}}_{\bullet j}^0$ and $\widetilde{\boldsymbol{V}}_{\bullet j}^{[t]}$, $\nabla\mathcal{L}(\widehat{\boldsymbol{V}}_{\bullet j}^0) = n^{-1}\sum_{i=1}^n \boldsymbol{X}_{i\bullet}(-Y_{ij} + \varphi_j(\boldsymbol{X}_{i\bullet}^\top \widehat{\boldsymbol{V}}_{\bullet j}^0)) = -n^{-1}\boldsymbol{X}^\top\widehat{\boldsymbol{\xi}}_j$ with $\widehat{\boldsymbol{\xi}}_j = \boldsymbol{Y}_j - \varphi_j(\boldsymbol{X}\widehat{\boldsymbol{V}}_{\bullet j}^0)$. By the optimality condition of (6) at iteration t,

$$0 \le \big(\widehat{\boldsymbol{V}}_{\bullet j}^0 - \widetilde{\boldsymbol{V}}_{\bullet j}^{[t]}\big)^\top \big(\nabla\mathcal{L}(\widetilde{\boldsymbol{V}}_{\bullet j}^{[t]}) + \gamma_j\tau_j\nabla\big\|\big(\widetilde{\boldsymbol{V}}_{\bullet j}^{[t]}\big)_{\left(S_j^{[t-1]}\right)^c}\big\|_1\big), \tag{25}$$

where $\|\cdot\|_1$ denotes the ℓ_1-norm. On the other hand, by the optimality condition of the oracle estimator $\widehat{\boldsymbol{V}}_{\bullet j}^0$: $\boldsymbol{X}_{S_j^0}^\top(\boldsymbol{Y}_j - \varphi_j(\boldsymbol{X}\widehat{\boldsymbol{V}}_{\bullet j}^0)) = \boldsymbol{X}^\top\widehat{\boldsymbol{\xi}}_j = \boldsymbol{0}$ on S_j^0, implying that $(\boldsymbol{R}_j)_{S_j^{[t-1]}\cap S_j^0} = \boldsymbol{0}$, where $\boldsymbol{R}_j = \boldsymbol{X}^\top\widehat{\boldsymbol{\xi}}_j/n - \gamma_j\tau_j\nabla\big\|\big(\widetilde{\boldsymbol{V}}_{\bullet j}^{[t]}\big)_{\left(S_j^{[t-1]}\right)^c}\big\|_1$. Let $S_j^0\Delta S_j^{[t-1]} = (S_j^0\backslash S_j^{[t-1]}) \cup (S_j^{[t-1]}\backslash S_j^0)$, where Δ denotes the symmetric difference.

Hence, combination of (24) and (25) yields that

$$\begin{aligned}
&\big(\widetilde{\boldsymbol{V}}_{\bullet j}^{[t]} - \widehat{\boldsymbol{V}}_{\bullet j}^0\big)^\top \nabla^2\mathcal{L}(\overline{\boldsymbol{V}}_{\bullet j})\big(\widetilde{\boldsymbol{V}}_{\bullet j}^{[t]} - \widehat{\boldsymbol{V}}_{\bullet j}^0\big) \le \big(\widetilde{\boldsymbol{V}}_{\bullet j}^{[t]} - \widehat{\boldsymbol{V}}_{\bullet j}^0\big)^\top\big(\boldsymbol{X}^\top\widehat{\boldsymbol{\xi}}_j/n - \gamma_j\tau_j\nabla\big\|\big(\widetilde{\boldsymbol{V}}_{\bullet j}^{[t]}\big)_{\left(S_j^{[t-1]}\right)^c}\big\|_1\big)\\
&\le \big(\widetilde{\boldsymbol{V}}_{\bullet j}^{[t]} - \widehat{\boldsymbol{V}}_{\bullet j}^0\big)^\top_{S_j^0\Delta S_j^{[t-1]}}(\boldsymbol{R}_j)_{S_j^0\Delta S_j^{[t-1]}} + \big(\widetilde{\boldsymbol{V}}_{\bullet j}^{[t]} - \widehat{\boldsymbol{V}}_{\bullet j}^0\big)^\top_{\left(S_j^0\cup S_j^{[t-1]}\right)^c}(\boldsymbol{R}_j)_{\left(S_j^0\cup S_j^{[t-1]}\right)^c}\\
&\le \big\|\big(\widetilde{\boldsymbol{V}}_{\bullet j}^{[t]} - \widehat{\boldsymbol{V}}_{\bullet j}^0\big)_{S_j^0\Delta S_j^{[t-1]}}\big\|_1\big(\|\boldsymbol{X}^\top\widehat{\boldsymbol{\xi}}_j/n\|_\infty + \gamma_j\tau_j\big)\\
&+ \big\|\big(\widetilde{\boldsymbol{V}}_{\bullet j}^{[t]} - \widehat{\boldsymbol{V}}_{\bullet j}^0\big)_{\left(S_j^0\cup S_j^{[t-1]}\right)^c}\big\|_1\big(\|\boldsymbol{X}^\top\widehat{\boldsymbol{\xi}}_j/n\|_\infty - \gamma_j\tau_j\big),
\end{aligned} \tag{26}$$

where the last inequality holds since $\big(\widetilde{\boldsymbol{V}}_{\bullet j}^{[t]} - \widehat{\boldsymbol{V}}_{\bullet j}^0\big)^\top_{\left(S_j^0\cup S_j^{[t-1]}\right)^c}\big(\nabla\big\|\big(\widetilde{\boldsymbol{V}}_{\bullet j}^{[t]}\big)_{\left(S_j^0\cup S_j^{[t-1]}\right)^c}\big\|_1\big) = \big\|\big(\widetilde{\boldsymbol{V}}_{\bullet j}^{[t]} - \widehat{\boldsymbol{V}}_{\bullet j}^0\big)_{\left(S_j^0\cup S_j^{[t-1]}\right)^c}\big\|_1$. Note that $\big(\widetilde{\boldsymbol{V}}_{\bullet j}^{[t]} - \widehat{\boldsymbol{V}}_{\bullet j}^0\big)^\top\nabla^2\mathcal{L}(\overline{\boldsymbol{V}}_{\bullet j})\big(\widetilde{\boldsymbol{V}}_{\bullet j}^{[t]} - \widehat{\boldsymbol{V}}_{\bullet j}^0\big) \ge 0$ since $\nabla^2\mathcal{L}(\overline{\boldsymbol{V}}_{\bullet j})$ is positive-definite. By (26),

$$\big\|\big(\widetilde{\boldsymbol{V}}_{\bullet j}^{[t]} - \widehat{\boldsymbol{V}}_{\bullet j}^0\big)_{\left(S_j^0\cup S_j^{[t-1]}\right)^c}\big\|_1\big(\gamma_j\tau_j - \big\|\boldsymbol{X}^\top\widehat{\boldsymbol{\xi}}_j/n\big\|_\infty\big) \le \big\|\big(\widetilde{\boldsymbol{V}}_{\bullet j}^{[t]} - \widehat{\boldsymbol{V}}_{\bullet j}^0\big)_{S_j^0\Delta S_j^{[t-1]}}\big\|_1\big(\big\|\boldsymbol{X}^\top\widehat{\boldsymbol{\xi}}_j/n\big\|_\infty + \gamma_j\tau_j\big).$$

Note, on event $\mathcal{E}_j$, $\big\|\boldsymbol{X}^\top\widehat{\boldsymbol{\xi}}_j/n\big\|_\infty \le \gamma_j\tau_j/2$, and thus

$$\Big\|\big(\widetilde{\boldsymbol{V}}_{\bullet j}^{[t]} - \widehat{\boldsymbol{V}}_{\bullet j}^0\big)_{\left(S_j^0\cup S_j^{(t-1)}\right)^c}\Big\|_1 \le 3\Big\|\big(\widetilde{\boldsymbol{V}}_{\bullet j}^{[t]} - \widehat{\boldsymbol{V}}_{\bullet j}^0\big)_{S_j^0\Delta S_j^{[t-1]}}\Big\|_1 \le 3\Big\|\big(\widetilde{\boldsymbol{V}}_{\bullet j}^{[t]} - \widehat{\boldsymbol{V}}_{\bullet j}^0\big)_{S_j^0\cup S_j^{[t-1]}}\Big\|_1.$$

Note that $\left|S_j^0 \cup S_j^{[t-1]}\right| \leq 2K_j^0$. By Assumption 4 and (26),

$$
\begin{aligned}
& m\|\widetilde{\boldsymbol{V}}_{\bullet j}^{[t]} - \widehat{\boldsymbol{V}}_{\bullet j}^0\|_2^2 \leq (\widetilde{\boldsymbol{V}}_{\bullet j}^{[t]} - \widehat{\boldsymbol{V}}_{\bullet j}^0)^\top \nabla^2 \mathcal{L}(\overline{\boldsymbol{V}}_{\bullet j})(\widetilde{\boldsymbol{V}}_{\bullet j}^{[t]} - \widehat{\boldsymbol{V}}_{\bullet j}^0) \\
& \leq (\|\boldsymbol{X}^\top \widehat{\boldsymbol{\xi}}_j/n\|_\infty + \gamma_j\tau_j)\|(\widetilde{\boldsymbol{V}}_{\bullet j}^{[t]} - \widehat{\boldsymbol{V}}_{\bullet j}^0)_{S_j^0 \Delta S_j^{[t-1]}}\|_1 + (\|\boldsymbol{X}^\top \widehat{\boldsymbol{\xi}}_j/n\|_\infty - \gamma_j\tau_j) \\
& \|(\widetilde{\boldsymbol{V}}_{\bullet j}^{[t]} - \widehat{\boldsymbol{V}}_{\bullet j}^0)_{(S_j^0 \cup S_j^{[t-1]})^c}\|_1 \\
& \leq (\|\boldsymbol{X}^\top \widehat{\boldsymbol{\xi}}_j/n\|_\infty + \gamma_j\tau_j)\|(\widetilde{\boldsymbol{V}}_{\bullet j}^{[t]} - \widehat{\boldsymbol{V}}_{\bullet j}^0)_{S_j^0 \Delta S_j^{[t-1]}}\|_1, \\
& \leq 1.5\gamma_j\tau_j\sqrt{|S_j^0 \Delta S_j^{[t-1]}|} \cdot \|\widetilde{\boldsymbol{V}}_{\bullet j}^{[t]} - \widehat{\boldsymbol{V}}_{\bullet j}^0\|_2,
\end{aligned} \tag{27}
$$

where the last inequality follows from the Cauchy-Schwarz inequality and $\|\boldsymbol{X}^\top \widehat{\boldsymbol{\xi}}_j/n\|_\infty \leq 0.5\gamma_j\tau_j$ on $\mathcal{E}_j$. Hence,

$$
\|\widetilde{\boldsymbol{V}}_{\bullet j}^{[t]} - \widehat{\boldsymbol{V}}_{\bullet j}^0\|_2/\tau_j \leq (1.5\gamma_j/m)\sqrt{2K_j^0} \leq \sqrt{K_j^0}, \tag{28}
$$

since $|S_j^0 \Delta S_j^{[t-1]}| \leq |S_j^0 \cup S_j^{[t-1]}| \leq 2K_j^0$ and $\gamma_j \leq m/6$ by Condition (1) of Theorem 4. Moreover, $\|\widetilde{\boldsymbol{V}}_{\bullet j}^{[t]} - \widehat{\boldsymbol{V}}_{\bullet j}^0\|_2^2 \geq |\mathrm{FP}_j^{[t]}| \cdot \tau_j^2$ since $|\widetilde{\boldsymbol{V}}_{lj}^{[t]} - \widehat{\boldsymbol{V}}_{lj}^0| = |\widetilde{\boldsymbol{V}}_{lj}^{[t]}| > \tau_j$ for any $l \in \mathrm{FP}_j^{[t]} = S_j^{[t]} \backslash S_j^0$. By (28), $|\mathrm{FP}_j^{[t]}| \leq \|\widetilde{\boldsymbol{V}}_{\bullet j}^{[t]} - \widehat{\boldsymbol{V}}_{\bullet j}^0\|_2^2/\tau_j^2 \leq K_j^0$. Therefore, $|S_j^0 \cup S_j^{[t]}| = |S_j^0| + |\mathrm{FP}_j^{[t]}| \leq 2K_j^0$.

Step 2: Suppose $|\mathrm{FP}_j^{[t]}| + |\mathrm{FN}_j^{[t]}| \geq 1$. Similarly,

$$
\|\widetilde{\boldsymbol{V}}_{\bullet j}^{[t]} - \widehat{\boldsymbol{V}}_{\bullet j}^0\|_2^2 \geq (|\mathrm{FP}_j^{[t]}| + |\mathrm{FN}_j^{[t]}|)(0.5\tau_j)^2,
$$

since $|\widetilde{V}_{lj}^{[t]} - \widehat{V}_{lj}^0| \geq |\widetilde{V}_{lj}^{[t]} - V_{lj}^0| - |\widehat{V}_{lj}^0 - V_{lj}^0| \geq \tau_j - 0.5\tau_j$ for any $l \in \mathrm{FP}_j^{[t]} \cup \mathrm{FN}_j^{[t]}$, by Assumption 6. Therefore, $\sqrt{|\mathrm{FP}_j^{[t]}| + |\mathrm{FN}_j^{[t]}|} \leq \|\widetilde{\boldsymbol{V}}_{\bullet j}^{[t]} - \widehat{\boldsymbol{V}}_{\bullet j}^0\|_2/0.5\tau_j$. Moreover, by (27) and the Cauchy-Schwarz inequality, $m\|\widetilde{\boldsymbol{V}}_{\bullet j}^{[t]} - \widehat{\boldsymbol{V}}_{\bullet j}^0\|_2^2 \leq 1.5\gamma_j\tau_j\|(\widetilde{\boldsymbol{V}}_{\bullet j}^{[t]} - \widehat{\boldsymbol{V}}_{\bullet j}^0)_{S_j^0 \Delta S_j^{[t-1]}}\|_1 \leq 1.5\gamma_j\tau_j\sqrt{|S_j^0 \Delta S_j^{[t-1]}|} \cdot \|\widetilde{\boldsymbol{V}}_{\bullet j}^{[t]} - \widehat{\boldsymbol{V}}_{\bullet j}^0\|_2$. Hence, $\|\widetilde{\boldsymbol{V}}_{\bullet j}^{[t]} - \widehat{\boldsymbol{V}}_{\bullet j}^0\|_2/\tau_j \leq (1.5\gamma_j/m)\sqrt{|\mathrm{FP}_j^{[t-1]}| + |\mathrm{FN}_j^{[t-1]}|}$. By Conditions (1) and (2) of Theorem 4:

$$
\sqrt{|\mathrm{FP}_j^{[t]}| + |\mathrm{FN}_j^{[t]}|} \leq \frac{\|\widetilde{\boldsymbol{V}}_{\bullet j}^{[t]} - \widehat{\boldsymbol{V}}_{\bullet j}^0\|_2}{0.5\tau_j} \leq \frac{3\gamma_j}{m}\sqrt{|\mathrm{FP}_j^{[t-1]}| + |\mathrm{FN}_j^{[t-1]}|} \leq 0.5\sqrt{|\mathrm{FP}_j^{[t-1]}| + |\mathrm{FN}_j^{[t-1]}|}.
$$

Iterating this process implies that $\sqrt{|\mathrm{FP}_j^{[t]}| + |\mathrm{FN}_j^{[t]}|} \leq (\frac{1}{2})^t\sqrt{|S_j^0| + |S_j^{[0]}|}$, $t = 0, 1, \ldots$. If $t \geq T = 1 + \lceil \log(2K_j^0)/\log 4 \rceil$, then $|\mathrm{FP}_j^{[t]}| + |\mathrm{FN}_j^{[t]}| < 1$ or $\mathrm{FP}_j^{[t]} = \mathrm{FN}_j^{[t]} = \emptyset$ on event $\mathcal{E}_j$. Consequently, $\{l : \widetilde{V}_{lj}^{[T]} \neq 0\} = \{l : V_{lj}^0 \neq 0\} = S_j^0$.

Step 3: To bound
$P\big(\bigcup_{j=1}^p \mathcal{E}_j^c\big)$, recall that $\mathcal{E}_j = \big\{\|\boldsymbol{X}^\top \widehat{\boldsymbol{\xi}}_j/n\|_\infty \leq 0.5\gamma_j\tau_j\big\} \cap \big\{\|\widehat{\boldsymbol{V}}_{\bullet j}^0 - \boldsymbol{V}_{\bullet j}^0\|_\infty \leq 0.5\tau_j\big\}$. Next, we bound the two events in $\mathcal{E}_j^c$ separately. For the first event, by the triangular inequality,

$$
\begin{aligned}
& \mathbb{P}(\|\boldsymbol{X}^\top \widehat{\boldsymbol{\xi}}_j/n\|_\infty > 0.5\gamma_j\tau_j) = \mathbb{P}(\|\boldsymbol{X}^\top(\boldsymbol{Y}_j - \varphi_j(\boldsymbol{X}\widehat{\boldsymbol{V}}_{\bullet j}^0))/n\|_\infty > 0.5\gamma_j\tau_j) \\
& \leq \mathbb{P}(\|\boldsymbol{X}^\top(\boldsymbol{Y}_j - \varphi_j(\boldsymbol{X}\boldsymbol{V}_{\bullet j}^0))/n\|_\infty > 0.25\gamma_j\tau_j) \\
& + \mathbb{P}(\|\boldsymbol{X}^\top(\varphi_j(\boldsymbol{X}\boldsymbol{V}_{\bullet j}^0) - \varphi_j(\boldsymbol{X}\widehat{\boldsymbol{V}}_{\bullet j}^0))/n\|_\infty > 0.25\gamma_j\tau_j).
\end{aligned} \tag{29}
$$

By Assumption 5, $|X_{ik}| \le c_1$. By Assumption 3, $Y_{ij} - \varphi_j(\boldsymbol{V}^{0}_{\bullet j}{}^{\top}\boldsymbol{X}_{i\bullet})$ is sub-exponential with the bound M. Hence, by Bernstein's inequality (Theorem 2.8.2 of Vershynin (2018)), for any given $k = 1, \cdots, q$,

$$\mathbb{P}\left(\left|\sum_{i=1}^{n} X_{ik}(Y_{ij} - \mathbb{E}[Y_{ij}|\boldsymbol{X}])/n\right| \ge 0.25\gamma_j\tau_j\right) \le 2\exp\left(-\min\left(\frac{\gamma_j^2\tau_j^2 n}{32M^2c_1^2}, \frac{\gamma_j\tau_j n}{8Mc_1}\right)\right).$$

Note that $\left\|\boldsymbol{X}^{\top}(\boldsymbol{Y}_j - \varphi_j(\boldsymbol{X}\boldsymbol{V}^0_{\bullet j}))/n\right\|_{\infty} = \max_{k=1}^{q} |\sum_{i=1}^{n} X_{ik}(Y_{ij} - \mathbb{E}[Y_{ij}|\boldsymbol{X}])/n|$. The union bound yields, for the first quantity in (29), that

$$\begin{aligned}&\mathbb{P}\big(\left\|\boldsymbol{X}^{\top}(\boldsymbol{Y}_j - \varphi_j(\boldsymbol{X}\boldsymbol{V}^0_{\bullet j}))/n\right\|_{\infty} > 0.25\gamma_j\tau_j\big) \le 2q\exp\big(-\min\big(\frac{\gamma_j^2\tau_j^2 n}{32M^2c_1^2}, \frac{\gamma_j\tau_j n}{8Mc_1}\big)\big),\\ &\le 2\exp(-2\log n - \log q) = 2n^{-2}q^{-1},\end{aligned} \tag{30}$$

by the choice of γ_j and τ_j, that is, $\gamma_j\tau_j \ge \sqrt{64M^2c_1^2(\log q + \log n)/n}$.

For the second quantity in (29), we bound $\|\boldsymbol{X}^{\top}(\varphi_j(\boldsymbol{X}\boldsymbol{V}^0_{\bullet j}) - \varphi_j(\boldsymbol{X}\widehat{\boldsymbol{V}}^0_{\bullet j}))/n\|_{\infty}$. Towards this end, note that $V^0_{kj} = \widehat{V}^0_{kj} = 0$ on $k \notin S^0_j$. Therefore, for $\boldsymbol{V}^0_{\bullet j}$ and $\widehat{\boldsymbol{V}}^0_{\bullet j}$ constrained on the set S^0_j, $\boldsymbol{V}^0_{\bullet j} = \boldsymbol{V}^0_{S^0_j, j}$ and $\widehat{\boldsymbol{V}}^0_{\bullet j} = \widehat{\boldsymbol{V}}^0_{S^0_j, j}$. Then, by Assumption 3,

$$\|\boldsymbol{X}^{\top}(\varphi_j(\boldsymbol{X}\boldsymbol{V}^0_{\bullet j}) - \varphi_j(\boldsymbol{X}\widehat{\boldsymbol{V}}^0_{\bullet j}))\|_{\infty} \le L_1\|\boldsymbol{X}^{\top}\boldsymbol{X}_{S^0_j}(\boldsymbol{V}^0_{S^0_j, j} - \widehat{\boldsymbol{V}}^0_{S^0_j, j})\|_{\infty}, \tag{31}$$

for some Lipschitz constant $L_1 > 0$. Moreover, by Lemma 7, for the oracle estimator constrained on S^0_j, namely, $\hat{\boldsymbol{V}}^0_{S^0_j, j}$, $\widehat{\boldsymbol{V}}^0_{S^0_j, j} - \boldsymbol{V}^0_{S^0_j, j} = (\boldsymbol{X}^{\top}_{S^0_j}\boldsymbol{M}\boldsymbol{X}_{S^0_j})^{-1}\boldsymbol{X}^{\top}_{S^0_j}(\boldsymbol{Y}_j - \boldsymbol{\zeta}^0 - \boldsymbol{r})$, where $\boldsymbol{M}$, $\boldsymbol{\zeta}^0$ and $\boldsymbol{r}$ will be defined in Lemma 7. Let $\boldsymbol{K} = \boldsymbol{X}^{\top}\boldsymbol{X}_{S^0_j}(\boldsymbol{X}^{\top}_{S^0_j}\boldsymbol{M}\boldsymbol{X}_{S^0_j})^{-1}\boldsymbol{X}^{\top}_{S^0_j}$. Plugging the above expression into (31) yields that

$$\begin{aligned}&\mathbb{P}\big(\|\boldsymbol{X}^{\top}(\varphi_j(\boldsymbol{X}\boldsymbol{V}^0_{\bullet j}) - \varphi_j(\boldsymbol{X}\widehat{\boldsymbol{V}}^0_{\bullet j}))/n\|_{\infty} > 0.25\gamma_j\tau_j\big) \le \mathbb{P}\big(\|\boldsymbol{K}(\boldsymbol{Y}_j - \boldsymbol{\zeta}^0 - \boldsymbol{r})/n\|_{\infty} > \frac{\gamma_j\tau_j}{4L_1}\big)\\ &\le \mathbb{P}\big(\|\boldsymbol{K}(\boldsymbol{Y}_j - \boldsymbol{\zeta}^0)/n\|_{\infty} > \frac{\gamma_j\tau_j}{4L_1} - \|\boldsymbol{K}\boldsymbol{r}/n\|_{\infty}\big).\end{aligned} \tag{32}$$

By Assumption 5, there exists a constant $c_3 > 0$ such that $\|\boldsymbol{X}^{\top}\boldsymbol{X}_{S^0_j}(\boldsymbol{X}^{\top}_{S^0_j}\boldsymbol{M}\boldsymbol{X}_{S^0_j})^{-1}\boldsymbol{X}^{\top}_{S^0_j}\|_{\infty} \le c_3$ or $\max_{l=1}^{q} |K_{li}| \le c_3$. Then, by Lemmas 7 and 8 with the choice of τ_j and γ_j,

$$\begin{aligned}\|\boldsymbol{K}\boldsymbol{r}/n\|_{\infty} &= \max_{l=1}^{q} |\sum_{i=1}^{n} K_{li}r_i|/n \le \max_{l}\sum_{i=1}^{n}|K_{li}||r_i|/n \le c_3\sum_{i=1}^{n}|r_i|/n\\ &\le c_3L_2(\hat{\boldsymbol{V}}^0_{S^0_j, j} - \boldsymbol{V}^0_{S^0_j, j})^{\top}(\boldsymbol{X}^{\top}_{S^0_j}\boldsymbol{X}_{S^0_j}/n)(\hat{\boldsymbol{V}}^0_{S^0_j, j} - \boldsymbol{V}^0_{S^0_j, j})\\ &\le c_3c_{\max}L_2\|\hat{\boldsymbol{V}}^0_{S^0_j, j} - \boldsymbol{V}^0_{S^0_j, j}\|_2^2 \le c_3c_{\max}L_2\frac{16M^2c_1^2}{m^2}\cdot\frac{K^0_j\log(nK^0_j)}{n} \le \frac{1}{2}\cdot\frac{\gamma_j\tau_j}{4L_1},\end{aligned}$$

with probability at least $1 - 2\exp(-\log(K^0_j) - 2\log n) = 1 - 2(K^0_j)^{-1}n^{-2}$.

Next, in (32), we bound $\boldsymbol{K}(\boldsymbol{Y}_j - \boldsymbol{\zeta}^0)/n$. Note that $\max_{k=1}^{q} |K_{ki}| \le c_3$. By Assumption 3, $Y_{ij} - \zeta^0_{ij} = (Y_{ij} - \mathbb{E}[Y_{ij}|\boldsymbol{X}])$ is sub-exponential with the bound M. By Theorem 2.8.2 of Vershynin

(2018) and a union bound as in (30),

$$
\begin{aligned}
&\mathbb{P}\Big(\|\boldsymbol{X}^\top(\varphi_j(\boldsymbol{X}\boldsymbol{V}^0_{\bullet j})-\varphi_j(\boldsymbol{X}\widehat{\boldsymbol{V}}^0_{\bullet j}))/n\|_\infty > 0.25\gamma_j\tau_j\Big) \le \mathbb{P}\Big(\|\boldsymbol{K}(\boldsymbol{Y}_j-\boldsymbol{\zeta}^0)/n\|_\infty > 0.5\frac{\gamma_j\tau_j}{4L_1}\Big)\\
&\le 2q\exp\Big(-\min\big(\frac{\gamma_j^2\tau_j^2 n}{8M^2c_3^2\cdot 16L_1^2}, \frac{\gamma_j\tau_j n}{16Mc_3\cdot L_1}\big)\Big) + 2(K_j^0)^{-1}n^{-2}\\
&\le 2q\exp\Big(-\frac{\gamma_j^2\tau_j^2 n}{8M^2c_3^2\cdot 16L_1^2}\Big) + 2(K_j^0)^{-1}n^{-2}. \qquad (33)
\end{aligned}
$$

Combining (29), (30), and (33) yields that

$$
\begin{aligned}
&\mathbb{P}\left(\left\|\boldsymbol{X}^\top\widehat{\boldsymbol{\xi}}_j/n\right\|_\infty > 0.5\gamma_j\tau_j\right) = \mathbb{P}\left(\left\|\boldsymbol{X}^\top(\boldsymbol{Y}_j-\varphi_j(\boldsymbol{X}\widehat{\boldsymbol{V}}^0_{\bullet j}))/n\right\|_\infty > 0.5\gamma_j\tau_j\right)\\
&\le 2q\exp\left(-\frac{\gamma_j^2\tau_j^2 n}{32M^2c_1^2}\right) + 2q\exp\left(-\frac{\gamma_j^2\tau_j^2 n}{8M^2c_3^2\cdot 16L_1^2}\right) + 2(K_j^0)^{-1}n^{-2}\\
&\le 2n^{-2}q^{-1} + 2n^{-2}q^{-1} + 2(K_j^0)^{-1}n^{-2} \le 6n^{-2}q^{-1}.
\end{aligned}
$$

Next, we bound the second event $\{\|\widehat{\boldsymbol{V}}^0_{\bullet j}-\boldsymbol{V}^0_{\bullet j}\|_\infty \le 0.5\tau_j\}$ in $\mathcal{E}_j^c$. Since $\widehat{\boldsymbol{V}}^0_{\bullet j} = \boldsymbol{V}^0_{\bullet j} = 0$ on $(S_j^0)^c$, it suffices to consider the entries of $\widehat{\boldsymbol{V}}^0_{\bullet j}$ constrained on S_j^0, or the oracle estimator $\widehat{\boldsymbol{V}}^0_{S_j^0,j}$. By Lemma 7, $\widehat{\boldsymbol{V}}^0_{S_j^0,j} - \boldsymbol{V}^0_{S_j^0,j} = \boldsymbol{H}(\boldsymbol{Y}_j-\boldsymbol{\zeta}^0-\boldsymbol{r})$, where $\boldsymbol{H} = \big(\boldsymbol{X}_{S_j^0}^\top\boldsymbol{M}\boldsymbol{X}_{S_j^0}\big)^{-1}\boldsymbol{X}_{S_j^0}^\top = (H_{ki})$.

To bound $\|\boldsymbol{H}\boldsymbol{r}\|_\infty$, by Assumption 5, $\|(\boldsymbol{X}_{S_j^0}^\top\boldsymbol{M}\boldsymbol{X}_{S_j^0}/n)^{-1}\boldsymbol{X}_{S_j^0}^\top\|_\infty \le c_2$ or $\|\boldsymbol{H}\|_\infty \le c_2 n^{-1}$, for some constant $c_2 > 0$. By Lemmas 7 and 8 with the choice of τ_j,

$$
\begin{aligned}
\|\boldsymbol{H}\boldsymbol{r}\|_\infty &= \max_k |\boldsymbol{H}_{k\bullet}\boldsymbol{r}| = \max_k |\sum_{i=1}^n H_{ki}r_i| \le \max_k \sum_{i=1}^n |H_{ki}||r_i| \le c_2\sum_{i=1}^n |r_i|/n\\
&\le c_2L_2(\widehat{\boldsymbol{V}}^0_{S_j^0,j}-\boldsymbol{V}^0_{S_j^0,j})^\top(\boldsymbol{X}_{S_j^0}^\top\boldsymbol{X}_{S_j^0}/n)(\widehat{\boldsymbol{V}}^0_{S_j^0,j}-\boldsymbol{V}^0_{S_j^0,j})\\
&\le c_2c_{\max}L_2\|\widehat{\boldsymbol{V}}^0_{S_j^0,j}-\boldsymbol{V}^0_{S_j^0,j}\|_2^2 \le c_2c_{\max}L_2\frac{16M^2c_1^2}{m^2}\frac{K_j^0\log(nK_j^0)}{n} \le 0.25\tau_j. \qquad (34)
\end{aligned}
$$

To bound $\|\boldsymbol{H}(\boldsymbol{Y}_j-\boldsymbol{\zeta}^0)\|_\infty$, note that $\max_{k=1}^q |H_{ki}| \le c_2/n$. By Assumption 3, $Y_{ij}-\zeta^0_{ij} = (Y_{ij}-\mathbb{E}[Y_{ij}|\boldsymbol{X}])$ is sub-exponential with the bound M. By the triangular inequality, Theorem 2.8.2 of Vershynin (2018) and the same argument as in (30), we obtain that

$$
\begin{aligned}
&\mathbb{P}\big(\|\widehat{\boldsymbol{V}}^0_{\bullet j}-\boldsymbol{V}^0_{\bullet j}\|_\infty > 0.5\tau_j\big) \le \mathbb{P}\big(\boldsymbol{H}(\boldsymbol{Y}_j-\boldsymbol{\zeta}^0)\|_\infty > 0.5\tau_j - \|\boldsymbol{H}\boldsymbol{r}\|_\infty\big)\\
&\le \mathbb{P}\big(\|\boldsymbol{H}(\boldsymbol{Y}_j-\boldsymbol{\zeta}^0)\|_\infty > 0.25\tau_j\big) \le \mathbb{P}\big(\|\boldsymbol{H}\boldsymbol{\xi}_j\|_\infty > 0.25\tau_j\big)\\
&\le 2K_j^0\exp\big(-\min\big(\frac{\tau_j^2 n}{32M^2c_2^2}, \frac{\tau_j n}{8Mc_2}\big)\big) + 2(K_j^0)^{-1}n^{-2}.
\end{aligned}
$$

To conclude, on $\mathcal{E}_j$, $\widehat{S}_j \equiv S_j^{[T]} = S_j^0$, which means $\widehat{\boldsymbol{V}}_{\bullet j} = \widetilde{\boldsymbol{V}}^{[T]}_{\bullet j} = \widehat{\boldsymbol{V}}^0_{\bullet j}$. Hence, for $j = 1,\dots,p$,

$$
\begin{aligned}
&\mathbb{P}(\widehat{\boldsymbol{V}}_{\bullet j} \ne \widehat{\boldsymbol{V}}^0_{\bullet j}) \le \mathbb{P}(\mathcal{E}_j^c) \le 2q\exp\big(-\frac{\gamma_j^2\tau_j^2 n}{32M^2c_1^2}\big) + 2q\exp\big(-\frac{\gamma_j^2\tau_j^2 n}{8M^2c_3^2\cdot 16L_1^2}\big)\\
&+ 2K_j^0\exp\big(-\frac{\tau_j^2 n}{32M^2c_2^2}\big) + 2(K_j^0)^{-1}n^{-2} \le 8n^{-2}q^{-1}. \qquad (35)
\end{aligned}
$$

It remains to show that $\widehat{\boldsymbol{V}}^0_{\bullet j}$ is a global minimizer of (5) with high probability. Towards this end, we will show that Assumptions 4 and 6 imply the degree of separation condition (3) of Shen et al. (2013). To see this, let $g(y_{ij}|\theta, \boldsymbol{x}_i) = e^{-\ell(y_{ij},\theta^\top \boldsymbol{x}_i)}$ be a probability density for y_{ij} where we denote $\theta = \boldsymbol{V}_{\bullet j}$ for notation simplicity. In addition, denote $\theta^0 = (\boldsymbol{V}_{S^0_j,j}, \mathbf{0})$ and $\theta_{A_j} = (\mathbf{0}, \boldsymbol{V}_{A_j,j})$. By the mean value theorem, there exists $\overline{\theta}_0$ between θ_{A_j} and θ^0 such that

$$
\begin{aligned}
&\left(g^{1/2}(y_{ij}|\theta_{A_j}, \boldsymbol{x}_i) - g^{1/2}(y_{ij}|\theta^0, \boldsymbol{x}_i)\right)^2 = \left(\left(\nabla g^{1/2}(y_{ij}|\overline{\theta}_0, \boldsymbol{x}_i)\right)^\top (\theta_{A_j} - \theta^0)\right)^2 \\
&= \left((\nabla e^{-\ell(y_{ij},\overline{\theta}_0^\top \boldsymbol{x}_i)/2})^\top (\theta_{A_j} - \theta^0)\right)^2 = \frac{1}{4} e^{-\ell(y_{ij},\overline{\theta}_0^\top \boldsymbol{x}_i)} \left(\nabla \ell(y_{ij}, \overline{\theta}_0^\top \boldsymbol{x}_i)^\top (\theta_{A_j} - \theta^0)\right)^2.
\end{aligned}
$$

Then, the Hellinger distance can be written as:

$$
\begin{aligned}
&h^2\left(\theta_{A_j}, \theta^0\right) = \frac{1}{4}\left(\int \left(g^{1/2}(y_{ij}|\theta_{A_j}, \boldsymbol{x}_i) - g^{1/2}(y_{ij}|\theta^0, \boldsymbol{x}_i)\right)^2 d\mu(y_{ij})\right) \\
&= \frac{1}{16}\left(\int e^{-\ell(y_{ij},\overline{\theta}_0^\top \boldsymbol{x}_i)} (\theta_{A_j} - \theta^0)^\top \nabla \ell(y_{ij}, \overline{\theta}_0^\top \boldsymbol{x}_i) \nabla \ell(y_{ij}, \overline{\theta}_0^\top \boldsymbol{x}_i)^\top (\theta_{A_j} - \theta^0) d\mu(y_{ij})\right) \\
&= \frac{1}{16}(\theta_{A_j} - \theta^0)^\top \left(\int e^{-\ell(y_{ij},\overline{\theta}_0^\top \boldsymbol{x}_i)} \cdot \nabla \ell(y_{ij}, \overline{\theta}_0^\top \boldsymbol{x}_i) \nabla \ell(y_{ij}, \overline{\theta}_0^\top \boldsymbol{x}_i)^\top d\mu(y_{ij})\right)(\theta_{A_j} - \theta^0) \\
&= \frac{1}{16}(\theta_{A_j} - \theta^0)^\top \mathbb{E}_{\overline{\theta}_0}\left[\nabla^2 \ell(y'_{ij}, \overline{\theta}_0^\top \boldsymbol{x}_i)\right](\theta_{A_j} - \theta^0),
\end{aligned}
$$

where $\mathbb{E}_{\overline{\theta}_0}$ is the expectation with respect to $Y'_{ij} \sim g(y'_{ij}|\overline{\theta}_0^\top, \boldsymbol{x}_i)$ while the last equality follows by the fact that $\mathbb{E}_\theta\left[\nabla \log g(y_{ij}|\theta, \boldsymbol{x}_i) \nabla \log g(y_{ij}|\theta, \boldsymbol{x}_i)^\top\right] = -\mathbb{E}_\theta\left[\nabla^2 \log g(y_{ij}|\theta, \boldsymbol{x}_i)\right]$ for any θ.

Let $\widetilde{\theta} = \theta_{A_j} - \theta^0$. Then, $\|\widetilde{\theta}\|_2^2 \geq |S^0_j \setminus A_j| \|\boldsymbol{V}_{S^0_j,j}\|_2^2$. By the definition of $C_{\min}$ of Shen et al. (2013) and Assumption 4,

$$
\begin{aligned}
C_{\min} &= \min_{A_j \neq S^0_j, |A_j| \leq K^0_j} \frac{h^2\left(\theta_{A_j}, \theta^0\right)}{\max(|S^0_j \setminus A_j|, 1)} \\
&\geq \min_{A_j \neq S^0_j, |A_j| \leq K^0_j} |S^0_j \setminus A_j|^{-1} (\theta_{A_j} - \theta^0)^\top \mathbb{E}\left[\nabla^2 \ell(y_{ij}, \overline{\theta}_0^\top \boldsymbol{x}_i)\right](\theta_{A_j} - \theta^0) \\
&\geq m \|\boldsymbol{V}_{S^0_j,j}\|_2^2 \geq m(100Mc_2)^2 \frac{\log q + \log n}{n} \geq m(100Mc_2)^2 \frac{\log q}{n},
\end{aligned}
$$

where the last inequality uses Assumptions 4 and 6 and the fact $\overline{\theta}_0 = \theta_{A_j} + t(\theta_{A_j} - \theta^0)$, $t \in [0,1]$ so that $\|\overline{\theta}_0\|_0 \leq 2K^0_j$. This implies the degree of separation condition (3) of Shen et al. (2013). By Theorem 2 there, $\mathbb{P}\left(\widehat{\boldsymbol{V}}^0_{\bullet j} \text{ is not a global minimizer of (5)}\right) \leq 3\exp(-2(\log(q) + \log(n))), 1 \leq j \leq p$, implying that

$$
\mathbb{P}\left(\widehat{\boldsymbol{V}}^0_{\bullet j} \text{ is not a global minimizer of (5)}, 1 \leq j \leq p\right) \leq 3p\left(\exp(-2(\log(q) + \log(n)))\right).
$$

Hence, $\widehat{\boldsymbol{V}}_{\bullet j}$ is a global minimizer of (5) with probability tending to 1 as $n \to \infty$; note that $q \geq p$ by Assumption 1(B). Finally, we have shown that $\widehat{S}_j \equiv \{l : \widehat{V}_{lj} \neq 0\} = S^0_j \equiv \{l : V^0_{lj} \neq 0\}$, implying $\{(l,j) : \widehat{V}_{lj} \neq 0\} = \{(l,j) : V^0_{lj} \neq 0\}$. By Proposition 3, the estimated $\widehat{\mathcal{S}}$ via $\widehat{\boldsymbol{V}}$ reconstructs the true super-graph $\mathcal{S}^0$ correctly. This completes the proof. We next present proofs of the lemmas.

Lemma 7 (Expression of the oracle MLE $\widehat{\boldsymbol{V}}^0_{S_j^0,j}$) *Let $\widehat{\boldsymbol{V}}^0_{S_j^0,j}$ be the oracle MLE, defined as the minimizer of $\mathcal{L}(\boldsymbol{V}_{S_j^0,j}|\boldsymbol{Y}_j,\boldsymbol{X}_{S_j^0}) = n^{-1}\sum_{i=1}^n \Big(-Y_{ij}(\boldsymbol{x}_{i,S_j^0}^\top \boldsymbol{V}_{S_j^0,j}) + A_j(\boldsymbol{x}_{i,S_j^0}^\top \boldsymbol{V}_{S_j^0,j})\Big)$ over $\boldsymbol{V}_{S_j^0,j}$. Then,*

$$\widehat{\boldsymbol{V}}^0_{S_j^0,j} - \boldsymbol{V}^0_{S_j^0,j} = (\boldsymbol{X}_{S_j^0}^\top \boldsymbol{M}\boldsymbol{X}_{S_j^0})^{-1}\boldsymbol{X}_{S_j^0}^\top(\boldsymbol{Y}_j - \boldsymbol{\zeta}^0 - \boldsymbol{r}), \tag{36}$$

where $\boldsymbol{\zeta}^0 = (\zeta_1^0,\ldots,\zeta_n^0)$ with $\zeta_i^0 = \varphi_j(\boldsymbol{x}_{i,S_j^0}^\top \boldsymbol{V}^0_{S_j^0,j})$, $\boldsymbol{M}$ is a diagonal matrix with the ith diagonal $\boldsymbol{M}_{ii} = A_j''(\boldsymbol{X}_{i\bullet}^\top \boldsymbol{V}^0_{\bullet j})$ and $\boldsymbol{r} = (r_1,\cdots,r_p)$ is the integral form of the reminder for Taylor's expansion satisfying

$$\sum_{i=1}^n |r_i| \le L_2(\widehat{\boldsymbol{V}}^0_{S_j^0,j} - \boldsymbol{V}^0_{S_j^0,j})^\top(\sum_{i=1}^n \boldsymbol{x}_{i,S_j^0}\boldsymbol{x}_{i,S_j^0}^\top)(\widehat{\boldsymbol{V}}^0_{S_j^0,j} - \boldsymbol{V}^0_{S_j^0,j}), \tag{37}$$

for L_2 defined in Assumption 3.

Proof of Lemma 7. By the optimality condition for the constrained oracle MLE, for $k \in S_j^0$,

$$\begin{aligned}&\sum_{i=1}^n X_{ik}(y_{ij} - \varphi_j(\boldsymbol{x}_{i,S_j^0}^\top \widehat{\boldsymbol{V}}^0_{S_j^0,j})) = 0,\\ &\sum_{i=1}^n X_{ik}(y_{ij} - \varphi_j(\boldsymbol{x}_{i,S_j^0}^\top \boldsymbol{V}^0_{S_j^0,j}) + \varphi_j(\boldsymbol{x}_{i,S_j^0}^\top \boldsymbol{V}^0_{S_j^0,j}) - \varphi_j(\boldsymbol{x}_{i,S_j^0}^\top \widehat{\boldsymbol{V}}^0_{S_j^0,j})) = 0. \end{aligned}\tag{38}$$

A Taylor series expansion of $\varphi_j(\boldsymbol{x}_{i,S_j^0}^\top \boldsymbol{V}_{S_j^0,j})$ at $\boldsymbol{V}^0_{S_j^0,j}$ yields that

$$\varphi_j(\boldsymbol{x}_{i,S_j^0}^\top \widehat{\boldsymbol{V}}^0_{S_j^0,j}) = \varphi_j(\boldsymbol{x}_{i,S_j^0}^\top \boldsymbol{V}^0_{S_j^0,j}) + w_j(\boldsymbol{x}_{i,S_j^0}^\top \boldsymbol{V}^0_{S_j^0,j})\boldsymbol{x}_{i,S_j^0}^\top(\widehat{\boldsymbol{V}}^0_{S_j^0,j} - \boldsymbol{V}^0_{S_j^0,j}) + r_i, \tag{39}$$

where

$$r_i = \int_0^1 \varphi_j''(\boldsymbol{x}_{i,S_j^0}^\top(\widehat{\boldsymbol{V}}^0_{S_j^0,j} + t(\widehat{\boldsymbol{V}}^0_{S_j^0,j} - \boldsymbol{V}^0_{S_j^0,j})))(1-t)\,dt\,\Big((\widehat{\boldsymbol{V}}^0_{S_j^0,j} - \boldsymbol{V}^0_{S_j^0,j})^\top \boldsymbol{x}_{i,S_j^0}\boldsymbol{x}_{i,S_j^0}^\top(\widehat{\boldsymbol{V}}^0_{S_j^0,j} - \boldsymbol{V}^0_{S_j^0,j})\Big),$$

is the integral form of the remainder for Taylor's expansion as Li and Lederer (2019) and $w_j(\boldsymbol{x}^\top\boldsymbol{u}) = \varphi_j'(\boldsymbol{x}^\top\boldsymbol{u}) = A_j''(\boldsymbol{x}^\top\boldsymbol{u})$. Let $\boldsymbol{M}$ be a diagonal matrix whose ith diagonal $\boldsymbol{M}_{ii} = A_j''(\boldsymbol{V}^{0\,\top}_{\bullet j}\boldsymbol{X}_{i\bullet})$. Write (38) in a matrix form using (39):

$$\begin{aligned}\boldsymbol{X}_{S_j^0}^\top \boldsymbol{M}\boldsymbol{X}_{S_j^0}(\hat{\boldsymbol{V}}^0_{S_j^0,j} - \boldsymbol{V}^0_{S_j^0,j}) &= \boldsymbol{X}_{S_j^0}^\top(\boldsymbol{Y}_j - \boldsymbol{\zeta}^0 - \boldsymbol{r})\\ \hat{\boldsymbol{V}}^0_{S_j^0,j} - \boldsymbol{V}^0_{S_j^0,j} &= (\boldsymbol{X}_{S_j^0}^\top \boldsymbol{M}\boldsymbol{X}_{S_j^0})^{-1}\boldsymbol{X}_{S_j^0}^\top(\boldsymbol{Y}_j - \boldsymbol{\zeta}^0 - \boldsymbol{r}),\end{aligned}\tag{40}$$

where $\zeta_i^0 = \varphi_j(\boldsymbol{x}_{i,S_j^0}^\top \boldsymbol{V}^0_{S_j^0,j})$. Further, note that $\varphi_j(\boldsymbol{x}^\top\boldsymbol{u}) = A_j'(\boldsymbol{x}^\top\boldsymbol{u})$. Then, in the expression for r_i, $\varphi_j''\big(\boldsymbol{x}_{i,S_j^0}^\top(\widehat{\boldsymbol{V}}^0_{S_j^0,j} + t(\widehat{\boldsymbol{V}}^0_{S_j^0,j} - \boldsymbol{V}^0_{S_j^0,j}))\big) = g_i(t)$, where $g_i(t) = \varphi_j''(\eta_i(t)) = A_j'''(\eta_i(t))$ with $\eta_i(t) = \boldsymbol{x}_{i,S_j^0}^\top(\hat{\boldsymbol{V}}^0_{S_j^0,j} + t(\hat{\boldsymbol{V}}^0_{S_j^0,j} - \boldsymbol{V}^0_{S_j^0,j}))$, $t \in (0,1)$. By Assumption 3, $|g_i(t)| \le L_2$. Hence, (37) holds.

Lemma 8 (Rate of convergence under the ℓ_2-norm) *Under Assumption 4 (restricted strong convexity),*

$$\|\widehat{\boldsymbol{V}}^0_{S_j^0,j} - \boldsymbol{V}^0_{S_j^0,j}\|_2 \le \frac{2}{m}\sqrt{K_j^0}\big\|\boldsymbol{X}_{S_j^0}^\top(\boldsymbol{Y}_j - \varphi_j(\boldsymbol{X}_{S_j^0}\boldsymbol{V}^0_{S_j^0,j}))/n\big\|_\infty \le \frac{4Mc_1}{m}\sqrt{\frac{K_j^0\log(nK_j^0)}{n}},$$

with probability at least $1 - 2\exp(-\log(K_j^0) - 2\log n) = 1 - 2(K_j^0)^{-1}n^{-2}$.

Proof of Lemma 8. We follow the proof of Lee et al. (2015) and consider the entries of $\boldsymbol{V}^0_{\bullet j}$ on S^0_j. The negative log-likelihood is

$$\mathcal{L}(\boldsymbol{V}_{S^0_j,j}|\boldsymbol{Y}_j,\boldsymbol{X}_{S^0_j}) = n^{-1}\sum_{i=1}^{n}\left(-Y_{ij}(\boldsymbol{x}^\top_{i,S^0_j}\boldsymbol{V}_{S^0_j,j}) + A_j(\boldsymbol{x}^\top_{i,S^0_j}\boldsymbol{V}_{S^0_j,j})\right),$$

where $\boldsymbol{x}_{i,S^0_j}$ is a subvector of $\boldsymbol{x}_i$ with elements constrained on the indices S^0_j. By the definition of the oracle MLE, $\mathcal{L}(\widehat{\boldsymbol{V}}^0_{S^0_j,j}) \le \mathcal{L}(\boldsymbol{V}^0_{S^0_j,j})$. Taylor's expansion of $\mathcal{L}(\cdot)$ at $\boldsymbol{V}^0_{S^0_j,j}$ yields that

$$0 \ge \nabla\mathcal{L}(\boldsymbol{V}^0_{S^0_j,j})(\widehat{\boldsymbol{V}}^0_{S^0_j,j} - \boldsymbol{V}^0_{S^0_j,j}) + \frac{1}{2}(\widehat{\boldsymbol{V}}^0_{S^0_j,j} - \boldsymbol{V}^0_{S^0_j,j})^\top\nabla^2\mathcal{L}(\overline{\boldsymbol{V}}^0_{S^0_j,j})(\widehat{\boldsymbol{V}}^0_{S^0_j,j} - \boldsymbol{V}^0_{S^0_j,j}).$$

Let $\Delta = \widehat{\boldsymbol{V}}^0_{\bullet j} - \boldsymbol{V}^0_{\bullet j}$. Clearly, $0 = \|\Delta_{(S^0_j)^c}\|_1 \le 3\|\Delta_{S^0_j}\|_1$. Therefore, by the restricted strong convexity condition $\frac{1}{2}(\widehat{\boldsymbol{V}}^0_{\bullet j}-\boldsymbol{V}^0_{\bullet j})^\top\nabla^2\mathcal{L}(\overline{\boldsymbol{V}}^0_{\bullet j})(\widehat{\boldsymbol{V}}^0_{\bullet j}-\boldsymbol{V}^0_{\bullet j}) \ge \frac{m}{2}\|\widehat{\boldsymbol{V}}^0_{\bullet j}-\boldsymbol{V}^0_{\bullet j}\|_2^2$, which implies $\frac{1}{2}(\widehat{\boldsymbol{V}}^0_{S^0_j,j}-\boldsymbol{V}^0_{S^0_j,j})^\top\nabla^2\mathcal{L}(\overline{\boldsymbol{V}}^0_{S^0_j,j})(\widehat{\boldsymbol{V}}^0_{S^0_j,j} - \boldsymbol{V}^0_{S^0_j,j}) \ge \frac{m}{2}\|\widehat{\boldsymbol{V}}^0_{S^0_j,j} - \boldsymbol{V}^0_{S^0_j,j}\|_2^2$ as $\widehat{\boldsymbol{V}}^0_{(S^0_j)^c,j} = \boldsymbol{V}^0_{(S^0_j)^c,j} = \boldsymbol{0}$.

By the restricted strong convexity condition,

$$\nabla\mathcal{L}(\boldsymbol{V}^0_{S^0_j,j})(\widehat{\boldsymbol{V}}^0_{S^0_j,j} - \boldsymbol{V}^0_{S^0_j,j}) + \frac{m}{2}\|\widehat{\boldsymbol{V}}^0_{S^0_j,j} - \boldsymbol{V}^0_{S^0_j,j}\|_2^2 \le 0.$$

By the Hölder's inequality,

$$\frac{m}{2}\|\widehat{\boldsymbol{V}}^0_{S^0_j,j} - \boldsymbol{V}^0_{S^0_j,j}\|_2^2 \le -\nabla\mathcal{L}(\boldsymbol{V}^0_{S^0_j,j})(\widehat{\boldsymbol{V}}^0_{S^0_j,j} - \boldsymbol{V}^0_{S^0_j,j}) \le \|\nabla\mathcal{L}(\boldsymbol{V}^0_{S^0_j,j})\|_\infty\|\widehat{\boldsymbol{V}}^0_{S^0_j,j} - \boldsymbol{V}^0_{S^0_j,j}\|_1.$$

By the Cauchy–Schwarz inequality,

$$\frac{m}{2}\|\widehat{\boldsymbol{V}}^0_{S^0_j,j} - \boldsymbol{V}^0_{S^0_j,j}\|_2^2 \le \sqrt{K^0_j}\|\nabla\mathcal{L}(\boldsymbol{V}^0_{S^0_j,j})\|_\infty\|\widehat{\boldsymbol{V}}^0_{S^0_j,j} - \boldsymbol{V}^0_{S^0_j,j}\|_2,$$
$$\|\widehat{\boldsymbol{V}}^0_{S^0_j,j} - \boldsymbol{V}^0_{S^0_j,j}\|_2 \le \frac{2}{m}\sqrt{K^0_j}\|\nabla\mathcal{L}(\boldsymbol{V}^0_{S^0_j,j})\|_\infty,$$

where $\nabla\mathcal{L}(\boldsymbol{V}^0_{S^0_j,j}) = n^{-1}\boldsymbol{X}^\top_{S^0_j}(\boldsymbol{Y}_j - \varphi_j(\boldsymbol{X}_{S^0_j}\boldsymbol{V}^0_{S^0_j,j}))$. Therefore,

$$\mathbb{P}\big(\big\|\boldsymbol{X}^\top_{S^0_j}(\boldsymbol{Y}_j - \varphi_j(\boldsymbol{X}_{S^0_j}\boldsymbol{V}^0_{S^0_j,j}))/n\big\|_\infty > \epsilon\big) \le 2K^0_j\exp\big(-\min\big(\frac{n\epsilon^2}{2M^2c_1^2},\frac{n\epsilon}{2Mc_1}\big)\big).$$

Hence, $\|\widehat{\boldsymbol{V}}^0_{S^0_j,j} - \boldsymbol{V}^0_{S^0_j,j}\|_2 \le \frac{2}{m}\sqrt{K^0_j}\epsilon = \frac{4Mc_1}{m}\sqrt{\frac{K^0_j\log(nK^0_j)}{n}}$, with probability $1 - 2K^0_j\exp\big(-\min\big(\frac{n\epsilon^2}{2M^2c_1^2},\frac{n\epsilon}{2Mc_1}\big)\big) \ge 1 - 2\exp(-\log(K^0_j) - 2\log n) = 1 - 2(K^0_j)^{-1}n^{-2}$, $\|\widehat{\boldsymbol{V}}^0_{S^0_j,j} - \boldsymbol{V}^0_{S^0_j,j}\|_2 \le \frac{2}{m}\sqrt{K^0_j}\epsilon = \frac{4Mc_1}{m}\sqrt{\frac{K^0_j\log(nK^0_j)}{n}}$, where $\epsilon = 2Mc_1\sqrt{\frac{\log(nK^0_j)}{n}}$.

D.5 Proof of Theorem 5

For the jth equation, the TLP estimator minimizes:

$$\begin{aligned}
&(\widehat{\boldsymbol{W}}_{\overline{\text{in}}(j),j},\widehat{\boldsymbol{U}}_{\overline{\text{an}}(j),j},\widehat{\boldsymbol{\alpha}}_{\overline{\text{an}}(j),j})\\
&= \operatorname*{argmin}_{\boldsymbol{W}_{\overline{\text{in}}(j),j},\boldsymbol{U}_{\overline{\text{an}}(j),j},\boldsymbol{\alpha}_{\overline{\text{an}}(j),j}} n^{-1}\sum_{i=1}^{n} -Y_{ij}\left(\boldsymbol{W}^\top_{\overline{\text{in}}(j),j}\boldsymbol{X}_{i,\overline{\text{in}}(j)} + \boldsymbol{U}^\top_{\overline{\text{an}}(j),j}\boldsymbol{Y}_{i,\overline{\text{an}}(j)} + \boldsymbol{\alpha}^\top_{\overline{\text{an}}(j),j}\widehat{\boldsymbol{h}}_{i,\overline{\text{an}}(j)}\right)\\
&\qquad + A_j\left(\boldsymbol{W}^\top_{\overline{\text{in}}(j),j}\boldsymbol{X}_{i,\overline{\text{in}}(j)} + \boldsymbol{U}^\top_{\overline{\text{an}}(j),j}\boldsymbol{Y}_{i,\overline{\text{an}}(j)} + \boldsymbol{\alpha}^\top_{\overline{\text{an}}(j),j}\widehat{\boldsymbol{h}}_{i,\overline{\text{an}}(j)}\right)\\
&\text{subject to} \quad \sum_{k\in\overline{\text{an}}(j)} I(U_{kj}\neq 0) \le K_j, \quad \sum_{k\in\overline{\text{an}}(j)} I(\alpha_{kj}\neq 0) \le K'_j, \quad j = 1,\ldots,p.
\end{aligned}$$

In the absence of confounders ($\boldsymbol{h}_{i,\mathrm{an}(j)} = 0$), if we use standard constrained GLM regression without deconfounding, ($\widehat{\boldsymbol{h}}_{i,\overline{\mathrm{an}}(j)} = 0$), it is straightforward to show that $\widehat{\boldsymbol{U}}_{\overline{\mathrm{an}}(j),j} \to \boldsymbol{U}^0_{\mathrm{an}^0(j),j}$ and $\widehat{\boldsymbol{W}}_{\overline{\mathrm{in}}(j),j} \to \boldsymbol{W}^0_{\mathrm{in}^0(j),j}$ by standard high-dimensional statistics results.

We now show the causal graph selection consistency of the TLP estimator in the presence of the confounders. We follow the same proof procedure of Theorem 4. Denote the oracle M-estimator $\widehat{\boldsymbol{\theta}}^{ml} = (\widehat{\boldsymbol{W}}^{ml}_{\overline{\mathrm{in}}(j),j}, \widehat{\boldsymbol{U}}^{ml}_{\overline{\mathrm{an}}(j),j}, \widehat{\boldsymbol{\alpha}}^{ml}_{\overline{\mathrm{an}}(j),j})) = \operatorname{argmin} \mathcal{L}(\boldsymbol{\theta}|\boldsymbol{Y}_{\overline{\mathrm{an}}(j)}, \boldsymbol{X}_{\overline{\mathrm{in}}(j)}, \widehat{\boldsymbol{h}}_{\overline{\mathrm{an}}(j)})$ such that $\{k : \widehat{U}^{ml}_{kj} \neq 0\} = \{k : U^0_{kj} \neq 0\} = \mathrm{pa}^0(j)$, $\{l : \widehat{W}^{ml}_{lj} \neq 0\} = \{l : W^0_{lj} \neq 0\} = \mathrm{in}^0(j)$ and $\{k : \widehat{\alpha}^{ml}_{kj} \neq 0\} = \{k : \alpha^0_{kj} \neq 0\}$. Further, denote A^0_j as the set of non-zero indices of the concatenated vector $\boldsymbol{\theta}^0 = (\boldsymbol{W}^0_{\mathrm{in}^0(j),j}, \boldsymbol{U}^0_{\mathrm{an}^0(j),j}, \boldsymbol{\alpha}^0_{\mathrm{an}^0(j),j})$. Therefore, $\boldsymbol{\theta}^0_{A^0_j} = (\boldsymbol{W}^0_{\mathrm{in}^0(j),j}, \boldsymbol{U}^0_{\mathrm{pa}^0(j),j}, \boldsymbol{\alpha}^0_{\mathrm{an}^0(j),j})$ and $\widehat{\boldsymbol{\theta}}^{ml}_{A^0_j} = (\widehat{\boldsymbol{W}}^{ml}_{\mathrm{in}^0(j),j}, \widehat{\boldsymbol{U}}^{ml}_{\mathrm{pa}^0(j),j}, \widehat{\boldsymbol{\alpha}}^{ml}_{\mathrm{an}^0(j),j})$; $\mathrm{supp}(\boldsymbol{\theta}^0) = \mathrm{supp}(\widehat{\boldsymbol{\theta}}^{ml}) = A^0_j$. By the proof of Theorem 4, it suffices to bound the event $\left\{\left\|\widehat{\boldsymbol{\theta}}^{ml} - \boldsymbol{\theta}^0\right\|_\infty \leq 0.5\tau_j\right\}$, or equivalently, $\left\{\left\|\widehat{\boldsymbol{\theta}}^{ml}_{A^0_j} - \boldsymbol{\theta}^0_{A^0_j}\right\|_\infty \leq 0.5\tau_j\right\}$, as $\widehat{U}^{ml}_{kj} = U^0_{kj} = 0$ on $k \in (A^0_j)^c$. Alternatively, by Proposition 1 of Shen et al. (2012),

$$P\left(\widehat{\boldsymbol{\theta}} \neq \widehat{\boldsymbol{\theta}}^{ml}\right) \leq \exp\left(-c_2 n C_{\min}(\boldsymbol{\theta}^0) + 2\log(p+1) + 3\right),$$

where $\widehat{\boldsymbol{\theta}} = (\widehat{\boldsymbol{W}}_{\overline{\mathrm{in}}(j),j}, \widehat{\boldsymbol{U}}_{\overline{\mathrm{an}}(j),j}, \widehat{\boldsymbol{\alpha}}_{\overline{\mathrm{an}}(j),j})$ is the final TLP estimator at iteration T, i.e., $\widehat{\boldsymbol{\theta}}^{[T]}$. Therefore, $\left\|\widehat{\boldsymbol{\theta}}^{ml} - \boldsymbol{\theta}^0\right\|_\infty \leq 0.5\tau_j$ implies that $\left\|\widehat{\boldsymbol{\theta}} - \boldsymbol{\theta}^0\right\|_\infty \leq 0.5\tau_j$.

For the root equations, note that the confounder $\boldsymbol{h}_k$ is independent of the instrumental variable $\boldsymbol{X}_{\mathrm{in}^0(k)}$. Hence, the confounders do not interfere with the estimation of the coefficient $\boldsymbol{W}_{\mathrm{in}(k),k}$. By the standard GLM result, $\|\boldsymbol{W}^0_{\mathrm{in}^0(k),k} - \widehat{\boldsymbol{W}}^{ml}_{\mathrm{in}^0(k),k}\|_\infty \propto \sqrt{\frac{\log(p\widetilde{s})}{n}}$. We prove the error bound in detail in Lemma 11.

For the child equations, let $\boldsymbol{\theta}_{A^0_j} = (\boldsymbol{W}_{\mathrm{in}^0(j),j}, \boldsymbol{U}_{\mathrm{pa}^0(j),j}, \boldsymbol{\alpha}_{\mathrm{an}^0(j),j})$ and $\widetilde{\boldsymbol{Z}} = [\boldsymbol{X}_{\mathrm{in}^0(j)}, \boldsymbol{Y}_{\mathrm{pa}^0(j)}, \widehat{\boldsymbol{h}}_{\mathrm{an}^0(j)}]$. Let $s = \max_{1\leq j\leq p} \|\boldsymbol{U}^0_{\bullet j}\|_0$ and $\widetilde{s} = \max_{1\leq j\leq p} \|\boldsymbol{W}^0_{\bullet j}\|_0$. The log-likelihood that $\widehat{\boldsymbol{\theta}}^{ml}_{A^0_j} = (\widehat{\boldsymbol{W}}^{ml}_{\mathrm{in}^0(j),j}, \widehat{\boldsymbol{U}}^{ml}_{\mathrm{pa}^0(j),j}, \widehat{\boldsymbol{\alpha}}^{ml}_{\mathrm{an}^0(j),j})$ minimizes is:

$$\begin{aligned}
\mathcal{L}(\boldsymbol{\theta}_{A^0_j}|\widetilde{\boldsymbol{Z}}) &= \mathcal{L}(\boldsymbol{W}_{\mathrm{in}^0(j),j}, \boldsymbol{U}_{\mathrm{pa}^0(j),j}, \boldsymbol{\alpha}_{\mathrm{an}^0(j),j}|\boldsymbol{X}_{\mathrm{in}^0(j)}, \boldsymbol{Y}_{\mathrm{pa}^0(j)}, \widehat{\boldsymbol{h}}_{\mathrm{an}^0(j)}) \\
&= \frac{1}{n}\sum_{i=1}^n -Y_{ij}\left(\boldsymbol{W}^\top_{\mathrm{in}^0(j),j}\boldsymbol{X}_{i,\mathrm{in}^0(j)} + \boldsymbol{U}^\top_{\mathrm{pa}^0(j),j}\boldsymbol{Y}_{i,\mathrm{pa}^0(j)} + \boldsymbol{\alpha}^\top_{\mathrm{an}^0(j),j}\widehat{\boldsymbol{h}}_{i,\mathrm{an}^0(j)}\right) \\
&\quad + A_j\left(\boldsymbol{W}^\top_{\mathrm{in}^0(j),j}\boldsymbol{X}_{i,\mathrm{in}^0(j)} + \boldsymbol{U}^\top_{\mathrm{pa}^0(j),j}\boldsymbol{Y}_{i,\mathrm{pa}^0(j)} + \boldsymbol{\alpha}^\top_{\mathrm{an}^0(j),j}\widehat{\boldsymbol{h}}_{i,\mathrm{an}^0(j)}\right).
\end{aligned}$$

Since $\widehat{\boldsymbol{\theta}}^{ml}_{A^0_j} = (\widehat{\boldsymbol{W}}^{ml}_{\mathrm{in}^0(j),j}, \widehat{\boldsymbol{U}}^{ml}_{\mathrm{pa}^0(j),j}, \widehat{\boldsymbol{\alpha}}^{ml}_{\mathrm{an}^0(j),j})$ minimizes $\mathcal{L}(\boldsymbol{\theta}_{A^0_j}|\boldsymbol{Y}_{\mathrm{pa}^0(j)}, \boldsymbol{X}_{\mathrm{in}^0(j)}, \widehat{\boldsymbol{h}}_{\mathrm{an}^0(j)})$, by the KKT condition for the oracle MLE constrained on the true set:

$$\begin{aligned}
&\sum_{i=1}^n \widetilde{Z}_{ik}(y_{ij} - \varphi_j(\widetilde{\boldsymbol{z}}_i^\top \widehat{\boldsymbol{\theta}}^{ml}_{A^0_j})) = 0, \\
&\sum_{i=1}^n \widetilde{Z}_{ik}(y_{ij} - \varphi_j(\widetilde{\boldsymbol{z}}_i^\top \boldsymbol{\theta}^0_{A^0_j}) + \varphi_j(\widetilde{\boldsymbol{z}}_i^\top \boldsymbol{\theta}^0_{A^0_j}) - \varphi_j(\widetilde{\boldsymbol{z}}_i^\top \widehat{\boldsymbol{\theta}}^{ml}_{A^0_j})) = 0. \qquad (41)
\end{aligned}$$

As in Lemma 7, applying Taylor series expansion, (41) can be written in matrix form:

$$\widetilde{\boldsymbol{Z}}^\top \boldsymbol{M}\widetilde{\boldsymbol{Z}}(\widehat{\boldsymbol{\theta}}^{ml}_{A^0_j} - \boldsymbol{\theta}^0_{A^0_j}) = \widetilde{\boldsymbol{Z}}^\top(\boldsymbol{Y}_j - \varphi_j(\widetilde{\boldsymbol{z}}_i^\top \boldsymbol{\theta}^0_{A^0_j}) - \boldsymbol{r}).$$

Therefore, $\widehat{\boldsymbol{\theta}}^{ml}_{A_j^0} - \boldsymbol{\theta}^0_{A_j^0} = (\widetilde{\boldsymbol{Z}}^\top \boldsymbol{M}\widetilde{\boldsymbol{Z}})^{-1}\widetilde{\boldsymbol{Z}}^\top(\boldsymbol{Y}_j - \varphi_j(\widetilde{\boldsymbol{z}}_i^\top \boldsymbol{\theta}^0_{A_j^0}) - \boldsymbol{r})$. Then we calculate the ℓ_∞-norm of the estimation error:

$$\begin{aligned}
\|\widehat{\boldsymbol{\theta}}^{ml}_{A_j^0} - \boldsymbol{\theta}^0_{A_j^0}\|_\infty &= \|(\widetilde{\boldsymbol{Z}}^\top \boldsymbol{M}\widetilde{\boldsymbol{Z}})^{-1}\widetilde{\boldsymbol{Z}}^\top(\boldsymbol{Y}_j - \varphi_j(\widetilde{\boldsymbol{z}}_i^\top \boldsymbol{\theta}^0_{A_j^0}) - \boldsymbol{r})\|_\infty \\
&= \|(\widetilde{\boldsymbol{Z}}^\top \boldsymbol{M}\widetilde{\boldsymbol{Z}})^{-1}\widetilde{\boldsymbol{Z}}^\top(\boldsymbol{Y}_j - \varphi_j(\boldsymbol{z}_i^\top \boldsymbol{\theta}^0_{A_j^0}) + \varphi_j(\boldsymbol{z}_i^\top \boldsymbol{\theta}^0_{A_j^0}) - \varphi_j(\widetilde{\boldsymbol{z}}_i^\top \boldsymbol{\theta}^0_{A_j^0}) - \boldsymbol{r})\|_\infty \\
&\le \|\boldsymbol{H}(\boldsymbol{Y}_j - \varphi_j(\boldsymbol{z}_i^\top \boldsymbol{\theta}^0_{A_j^0}))\|_\infty + \|\boldsymbol{H}(\varphi_j(\boldsymbol{z}_i^\top \boldsymbol{\theta}^0_{A_j^0}) - \varphi_j(\widetilde{\boldsymbol{z}}_i^\top \boldsymbol{\theta}^0_{A_j^0}))\|_\infty + \|\boldsymbol{H}\boldsymbol{r}\|_\infty,
\end{aligned}$$

where $\boldsymbol{H} = (\widetilde{\boldsymbol{Z}}^\top \boldsymbol{M}\widetilde{\boldsymbol{Z}})^{-1}\widetilde{\boldsymbol{Z}}^\top$. Denote $\boldsymbol{Z} = [\boldsymbol{X}_{\mathrm{in}^0(j)}, \boldsymbol{Y}_{\mathrm{pa}^0(j)}, \boldsymbol{h}_{\mathrm{an}^0(j)}]$ as the true predictor variable. Again, by the bounded domain for interventions condition, there exists b_2 such that $\|n(\widetilde{\boldsymbol{Z}}^\top \boldsymbol{M}\widetilde{\boldsymbol{Z}})^{-1}\widetilde{\boldsymbol{Z}}^\top\|_\infty \le b_2$. Note $\widetilde{\boldsymbol{Z}} = [\boldsymbol{X}_{\mathrm{in}^0(j)}, \boldsymbol{Y}_{\mathrm{pa}^0(j)}, \widehat{\boldsymbol{h}}_{\mathrm{an}^0(j)}] \in \mathbb{R}^{2s+\widetilde{s}}$; $\boldsymbol{h}_{\mathrm{an}^0(j)}$ refers to a submatrix consisting of $\boldsymbol{h}_k, k \in \mathrm{an}^0(j)$ and $\boldsymbol{h}_{\mathrm{an}^0(j)}\boldsymbol{\alpha}^0_{\mathrm{an}^0(j),j} = \sum_{k\in \mathrm{an}^0(j)} \alpha^0_{kj}\boldsymbol{h}_k$.

Since $\mathbb{E}[\boldsymbol{Y}_j|\boldsymbol{Z}] = \varphi_j(\boldsymbol{X}_{\mathrm{in}^0(j)}\boldsymbol{W}^0_{\mathrm{in}^0(j),j} + \boldsymbol{Y}_{\mathrm{pa}^0(j)}\boldsymbol{U}^0_{\mathrm{pa}^0(j),j} + \sum_{k\in \mathrm{an}^0(j)} \alpha^0_{kj}\boldsymbol{h}_k) = \varphi_j(\boldsymbol{z}_i^\top \boldsymbol{\theta}^0_{A_j^0})$, the first term can be bounded by the Bernstein's inequality. That is,

$$\mathbb{P}(\|\boldsymbol{H}(\boldsymbol{Y}_j - \varphi_j(\boldsymbol{z}_i^\top \boldsymbol{\theta}^0_{A_j^0}))\|_\infty > \epsilon) \le 2(2s+\widetilde{s})\exp\left(-\min\left(\frac{n\epsilon^2}{2M^2b_2^2}, \frac{n\epsilon}{2Mb_2}\right)\right).$$

Setting $\epsilon = 2Mb_2\sqrt{\frac{\log(p(2s+\widetilde{s}))}{n}}$ leads to

$$\|\boldsymbol{H}(\boldsymbol{Y}_j - \varphi_j(\boldsymbol{z}_i^\top \boldsymbol{\theta}^0_{A_j^0}))\|_\infty \le 2Mb_2\sqrt{\frac{\log(p(2s+\widetilde{s}))}{n}},$$

with probability at least $1 - 2\exp(-2\log p - \log(2s+\widetilde{s})) = 1 - 2p^{-2}(2s+\widetilde{s})^{-1}$.

Note that for the second term,

$$\begin{aligned}
\varphi_j(\boldsymbol{z}_i^\top \boldsymbol{\theta}^0_{A_j^0}) - \varphi_j(\widetilde{\boldsymbol{z}}_i^\top \boldsymbol{\theta}^0_{A_j^0}) &= (\boldsymbol{z}_i^\top \boldsymbol{\theta}^0_{A_j^0} - \widetilde{\boldsymbol{z}}_i^\top \boldsymbol{\theta}^0_{A_j^0}) \odot \varphi_j'(\boldsymbol{\xi}) = \sum_k \alpha^0_{kj}(\boldsymbol{h}_k - \widehat{\boldsymbol{h}}_k) \odot \varphi_j'(\boldsymbol{\xi}) \\
&= -\sum_k \alpha^0_{kj}\Big(\Delta_k + (\boldsymbol{Y}_k - \mathbb{E}[\boldsymbol{Y}_k|\boldsymbol{X}_{\mathrm{in}^0(k)}, \boldsymbol{h}_k])\Big) \odot \varphi_j'(\boldsymbol{\xi}), \quad \text{by (43)},
\end{aligned}$$

where we use the fact that $\boldsymbol{z}_i^\top \boldsymbol{\theta}^0_{A_j^0} = \boldsymbol{X}_{\mathrm{in}^0(j)}\boldsymbol{W}^0_{\mathrm{in}^0(j),j} + \boldsymbol{Y}_{\mathrm{pa}^0(j)}\boldsymbol{U}^0_{\mathrm{pa}^0(j),j} + \sum_k \alpha^0_{kj}\boldsymbol{h}_k$ and $\widetilde{\boldsymbol{z}}_i^\top \boldsymbol{\theta}^0_{A_j^0} = \boldsymbol{X}_{\mathrm{in}^0(j)}\boldsymbol{W}^0_{\mathrm{in}^0(j),j} + \boldsymbol{Y}_{\mathrm{pa}^0(j)}\boldsymbol{U}^0_{\mathrm{pa}^0(j),j} + \sum_k \alpha^0_{kj}\widehat{\boldsymbol{h}}_k$. Therefore,

$$\|\boldsymbol{H}\big(\varphi_j(\boldsymbol{z}_i^\top \boldsymbol{\theta}^0_{A_j^0}) - \varphi_j(\widetilde{\boldsymbol{z}}_i^\top \boldsymbol{\theta}^0_{A_j^0})\big)\|_\infty \le \|\boldsymbol{H}\sum_k \alpha^0_{kj}\Delta_k \odot \varphi_j'(\boldsymbol{\xi})\|_\infty + \|\boldsymbol{H}\sum_k \alpha^0_{kj}(\boldsymbol{Y}_k - \mathbb{E}[\boldsymbol{Y}_k|\boldsymbol{X}_{\mathrm{in}^0(k)}, \boldsymbol{h}_k]) \odot \varphi_j'(\boldsymbol{\xi})\|_\infty.$$

Note that $|\varphi_j'(z)| \le L_1$ and $|H_{ij}| \le \frac{b_2}{n}$. The first quantity above is bounded by

$$\begin{aligned}
\|\sum_k \alpha^0_{kj}\Delta_k \odot \varphi_j'(\boldsymbol{\xi})\|_\infty &\le L_1\sum_k |\alpha^0_{kj}| \cdot \|\Delta_k\|_\infty, \\
\|\boldsymbol{H}\sum_k \alpha^0_{kj}\Delta_k \odot \varphi_j'(\boldsymbol{\xi})\|_\infty &\le n \cdot \frac{b_2}{n} \cdot L_1\sum_k |\alpha^0_{kj}| \cdot \|\Delta_k\|_\infty = b_2L_1\sum_k |\alpha^0_{kj}| \cdot \|\Delta_k\|_\infty.
\end{aligned}$$

For the second quantity, note that $\|\boldsymbol{H}\sum_k \alpha^0_{kj}(\boldsymbol{Y}_k - \mathbb{E}[\boldsymbol{Y}_k|\boldsymbol{X}_{\mathrm{in}^0(k)}, \boldsymbol{h}_k]) \odot \varphi_j'(\boldsymbol{\xi})\|_\infty = \|\sum_k \alpha^0_{kj}\boldsymbol{H}(\boldsymbol{Y}_k - \mathbb{E}[\boldsymbol{Y}_k|\boldsymbol{X}_{\mathrm{in}^0(k)}, \boldsymbol{h}_k]) \odot \varphi_j'(\boldsymbol{\xi})\|_\infty \le L_1\|\sum_k \alpha^0_{kj}\boldsymbol{H}(\boldsymbol{Y}_k - \mathbb{E}[\boldsymbol{Y}_k|\boldsymbol{X}_{\mathrm{in}^0(k)}, \boldsymbol{h}_k])\|_\infty$. By Assumption 3,

$\boldsymbol{Y}_k - \mathbb{E}[\boldsymbol{Y}_k|\boldsymbol{X}_{\text{in}^0(k)}, \boldsymbol{h}_k]$ is sub-exponential and therefore $\sum_k \alpha^0_{kj}\boldsymbol{H}(\boldsymbol{Y}_k - \mathbb{E}[\boldsymbol{Y}_k|\boldsymbol{X}_{\text{in}^0(k)}, \boldsymbol{h}_k])$ is also sub-exponential. We conclude that $\|\sum_k \alpha^0_{kj}\boldsymbol{H}(\boldsymbol{Y}_k - \mathbb{E}[\boldsymbol{Y}_k|\boldsymbol{X}_{\text{in}^0(k)}, \boldsymbol{h}_k])\|_\infty = o(\sqrt{\frac{\log(p(2s+\widetilde{s}))}{n}})$ with probability tending to 1 by the Bernstein's inequality, and therefore converges to zero with increased sample size. Therefore, $\|\boldsymbol{H}(\varphi_j(\boldsymbol{z}_i^\top \boldsymbol{\theta}^0_{A^0_j}) - \varphi_j(\widetilde{\boldsymbol{z}}_i^\top \boldsymbol{\theta}^0_{A^0_j}))\|_\infty \le b_2 L_1 \sum_k |\alpha^0_{kj}| \cdot \|\Delta_k\|_\infty$.

Last, for the remainder of Taylor series expansion $\boldsymbol{r}$, similar to Theorem 4 and Lemma 7, $|\boldsymbol{H}_{k\bullet}\boldsymbol{r}| = |\sum_{i=1}^n H_{ki} r_i| \le b_2 \sum_{i=1}^n |r_i|/n \le b_2 D(\boldsymbol{\theta}^0_{A^0_j} - \widehat{\boldsymbol{\theta}}^{ml}_{A^0_j})^\top (\boldsymbol{Z}^\top \boldsymbol{Z}/n)(\boldsymbol{\theta}^0_{A^0_j} - \widehat{\boldsymbol{\theta}}^{ml}_{A^0_j}) \le b_2 c_0 D \|\boldsymbol{\theta}^0_{A^0_j} - \widehat{\boldsymbol{\theta}}^{ml}_{A^0_j}\|_2^2$. Further, by Lemma 9, $\|\widehat{\boldsymbol{\theta}}^{ml}_{A^0_j} - \boldsymbol{\theta}^0_{A^0_j}\|_2 \le \frac{2}{m}\sqrt{2s+\widetilde{s}} \cdot \|\nabla \mathcal{L}(\boldsymbol{\theta}^0_{A^0_j}|\boldsymbol{Y}_{\text{pa}^0(j)}, \boldsymbol{X}_{\text{in}^0(j)}, \widehat{\boldsymbol{h}}_{\text{an}^0(j)})\|_\infty \le \frac{2}{m}\sqrt{2s+\widetilde{s}}\left[\eta_1\sqrt{\frac{\log(p(2s+\widetilde{s}))}{n}} + b_1 L_1 \sum_k |\alpha^0_{kj}| \cdot \|\Delta_k\|_\infty\right]$ with $\eta_1 = 2Mb_1$. Therefore,

$$
\begin{aligned}
|\boldsymbol{H}_{k\bullet}\boldsymbol{r}| &\le b_2 c_0 D \|\boldsymbol{\theta}^0_{A^0_j} - \widehat{\boldsymbol{\theta}}^{ml}_{A^0_j}\|_2^2 \\
&\le b_2 c_0 D \left(\frac{2}{m}\sqrt{2s+\widetilde{s}} \cdot \left(\eta_1 \sqrt{\frac{\log(p(2s+\widetilde{s}))}{n}} + b_1 L_1 \sum_k |\alpha^0_{kj}| \cdot \|\Delta_k\|_\infty\right)\right)^2 \\
&\le b_2 c_0 D \left(\left(\frac{2}{m}\sqrt{2s+\widetilde{s}}\right)^2 \cdot 2\left(\eta_1^2 \frac{\log(p(2s+\widetilde{s}))}{n} + \left(b_1 L_1 \sum_k |\alpha^0_{kj}| \cdot \|\Delta_k\|_\infty\right)^2\right)\right) \\
&\le 2 b_2 c_0 D \left(\frac{2}{m}\right)^2 \left(\eta_1^2 (2s+\widetilde{s}) \frac{\log(p(2s+\widetilde{s}))}{n} + (2s+\widetilde{s}) \cdot (b_1 L_1)^2 \cdot s \cdot \sum_k |\alpha^0_{kj}|^2 \cdot \|\Delta_k\|_\infty^2\right) \\
&\le b_2 c_0 D \left(\frac{8}{m^2}\eta_1^2 \cdot \sqrt{\frac{\log(p(2s+\widetilde{s}))}{n}} + \frac{8}{m^2}(b_1 L_1)^2 \sum_k |\alpha^0_{kj}|^2 \cdot \|\Delta_k\|_\infty\right),
\end{aligned}
$$

where the last inequality holds true as $n > (2s+\widetilde{s})^2 \log(p(2s+\widetilde{s}))$ and $(2s+\widetilde{s}) s \|\Delta_k\|_\infty \le (2s+\widetilde{s}) s \sqrt{\frac{\log(p\widetilde{s})}{n}} \le 1$. Also, we use the property $(\sum_{i=1}^s a_i)^2 \le s \sum_{i=1}^s a_i^2$. Combining the three terms leads to

$$
\begin{aligned}
\|\widehat{\boldsymbol{\theta}}^{ml}_{A^0_j} - \boldsymbol{\theta}^0_{A^0_j}\|_\infty &\le a_1 \sqrt{\frac{\log(p(2s+\widetilde{s}))}{n}} + \sum_k (b_2 L_1 |\alpha^0_{kj}| + \frac{8 b_2 c_0 D b_1^2 L_1^2 |\alpha^0_{kj}|^2}{m^2}) \|\Delta_k\|_\infty \\
&\le a_1 \sqrt{\frac{\log(p(2s+\widetilde{s}))}{n}} + \max_k \left(b_2 L_1 |\alpha^0_{kj}| + \frac{8 b_2 c_0 D b_1^2 L_1^2 |\alpha^0_{kj}|^2}{m^2}\right) \cdot \sum_k \|\Delta_k\|_\infty,
\end{aligned}
$$

with probability greater than $1 - 4\exp(-2\log p - \log(2s+\widetilde{s})) = 1 - 4p^{-2}(2s+\widetilde{s})^{-1}$. Here, $a_1 = 2Mb_2 + b_2 c_0 D(\frac{32}{m^2} M^2 b_1^2)$. Denote $\widehat{A}_j$ as the set of non-zero indices of the concatenated vector $\widehat{\boldsymbol{\theta}}$. Similar to the proof of Theorem 4, if τ_j is chosen such that $\tau_j \ge 2\left\|\widehat{\boldsymbol{\theta}}^{ml} - \boldsymbol{\theta}^0\right\|_\infty$, then $\widehat{\boldsymbol{\theta}} = \widehat{\boldsymbol{\theta}}^{ml}$ and $\widehat{A}_j = A^0_j$, that is, $\widehat{\text{pa}}(j) = \text{pa}^0(j)$, $\widehat{\text{in}}(j) = \text{in}^0(j)$ and $\widehat{\text{an}}(j) = \text{an}^0(j)$. Additionally, note that $\max(\|\widehat{\boldsymbol{U}}_{\bullet j} - \boldsymbol{U}^0_{\bullet j}\|_\infty, \|\widehat{\boldsymbol{W}}_{\bullet j} - \boldsymbol{W}^0_{\bullet j}\|_\infty) \le \|\widehat{\boldsymbol{\theta}}_{A^0_j} - \boldsymbol{\theta}^0_{A^0_j}\|_\infty$.

We have calculated the parameter estimation errors for the jth equation. Now we calculate the accumulated error for the confounder $\boldsymbol{h}_j$. Note that, by construction, $\widehat{\boldsymbol{h}}_j = \sum_k \widehat{\alpha}_{kj}\widehat{\boldsymbol{h}}_k + \widehat{\boldsymbol{\epsilon}}_j$ where $\widehat{\boldsymbol{\epsilon}}_j = \boldsymbol{Y}_j - \varphi_j(\boldsymbol{X}_{\widehat{\text{in}}(j)}\widehat{\boldsymbol{W}}_{\widehat{\text{in}}(j),j} + \boldsymbol{Y}_{\widehat{\text{pa}}(j)}\widehat{\boldsymbol{U}}_{\widehat{\text{pa}}(j),j} + \sum_{k\in\widehat{\text{an}}(j)} \widehat{\alpha}_{kj}\widehat{h}_k)$ is the residual estimated from the jth equation. In practice, we replace $\widehat{\boldsymbol{h}}_j$ with $\widehat{\boldsymbol{\epsilon}}_j$ in the algorithm as $\widehat{\boldsymbol{h}}_j$ is a linear combination of $\widehat{\boldsymbol{h}}_k$ and $\widehat{\boldsymbol{\epsilon}}_j$; including $\widehat{\boldsymbol{h}}_k$ and $\widehat{\boldsymbol{h}}_j$ in the GLM regression model is equivalent to including $\widehat{\boldsymbol{h}}_k$ and $\widehat{\boldsymbol{\epsilon}}_j$.

Note $\widehat{\boldsymbol{\theta}} = \widehat{\boldsymbol{\theta}}^{ml}$ implies $\widehat{\boldsymbol{U}}_{\widehat{\text{pa}}(j),j} = \widehat{\boldsymbol{U}}^{ml}_{\text{pa}^0(j),j}$, $\widehat{\boldsymbol{W}}_{\widehat{\text{in}}(j),j} = \widehat{\boldsymbol{W}}^{ml}_{\text{in}^0(j),j}$, and $\widehat{\alpha}_{kj} = \widehat{\alpha}^{ml}_{kj}$. We therefore have

$$\widehat{\boldsymbol{\epsilon}}_j = \boldsymbol{Y}_j - \varphi_j(\boldsymbol{X}_{\text{in}^0(j)}\widehat{\boldsymbol{W}}^{ml}_{\text{in}^0(j),j} + \boldsymbol{Y}_{\text{pa}^0(j)}\widehat{\boldsymbol{U}}^{ml}_{\text{pa}^0(j),j} + \sum_k \widehat{\alpha}^{ml}_{kj}\widehat{\boldsymbol{h}}_k).$$

On the other hand, by Assumption 2, $\boldsymbol{h}_j = \sum_k \alpha^0_{kj}\boldsymbol{h}_k + \boldsymbol{\epsilon}_j$, where $\boldsymbol{\epsilon}_j$ is orthogonal to the space spanned by $\{h_k : k \in \text{an}(j)\}$. Therefore, (2) can be written as:

$$\mathbb{E}\left[\boldsymbol{Y}_j | \boldsymbol{Y}_{\text{pa}(j)}, \boldsymbol{X}, \boldsymbol{h}\right] = \varphi_j(\boldsymbol{X}_{\text{in}^0(j)}\boldsymbol{W}^0_{\text{in}^0(j),j} + \boldsymbol{Y}_{\text{pa}^0(j)}\boldsymbol{U}^0_{\text{pa}^0(j),j} + \sum_k \alpha^0_{kj}\boldsymbol{h}_k + \boldsymbol{\epsilon}_j).$$

Similar to the root node case, we use $\boldsymbol{Y}_j - \varphi_j(\boldsymbol{X}_{\text{in}^0(j)}\boldsymbol{W}^0_{\text{in}^0(j),j} + \boldsymbol{Y}_{\text{pa}^0(j)}\boldsymbol{U}^0_{\text{pa}^0(j),j} + \sum_k \alpha^0_{kj}\boldsymbol{h}_k)$ to approximate $\boldsymbol{\epsilon}_j$ so that $\widehat{\boldsymbol{\epsilon}}_j$ and $\boldsymbol{\epsilon}_j$ can be compared at the same scale. Note by our previous calculation, the approximation error $\mathbb{E}\left[Y_{ij} | \boldsymbol{Y}_{\text{pa}(j)}, \boldsymbol{X}, \boldsymbol{h}\right] - Y_{ij}$ is sub-exponential with mean zero and the aggregate impact of this error term across samples is of the order of $o(\sqrt{\frac{\log(p(2s+\widetilde{s}))}{n}})$ by the Bernstein's inequality in subsequent equations. Toward this end,

$$\begin{aligned}
\Delta_j = \widehat{\boldsymbol{h}}_j - \boldsymbol{h}_j &= (\sum_k \widehat{\alpha}^{ml}_{kj}\widehat{\boldsymbol{h}}_k + \widehat{\boldsymbol{\epsilon}}_j) - (\sum_k \alpha^0_{kj}\boldsymbol{h}_k + \boldsymbol{\epsilon}_j) = \sum_k (\widehat{\alpha}^{ml}_{kj}\widehat{\boldsymbol{h}}_k - \alpha^0_{kj}\boldsymbol{h}_k) + (\widehat{\boldsymbol{\epsilon}}_j - \boldsymbol{\epsilon}_j) \\
&= I_1 - \varphi_j(\boldsymbol{X}_{\text{in}^0(j)}\widehat{\boldsymbol{W}}^{ml}_{\text{in}^0(j),j} + \boldsymbol{Y}_{\text{pa}^0(j)}\widehat{\boldsymbol{U}}^{ml}_{\text{pa}^0(j),j} + \sum_k \widehat{\alpha}^{ml}_{kj}\widehat{\boldsymbol{h}}_k) + \varphi_j(\boldsymbol{X}_{\text{in}^0(j)}\boldsymbol{W}^0_{\text{in}^0(j),j} + \boldsymbol{Y}_{\text{pa}^0(j)}\boldsymbol{U}^0_{\text{pa}^0(j),j} + \sum_k \alpha^0_{kj}\boldsymbol{h}_k) \\
&= I_1 + \varphi'_j(\boldsymbol{\xi}) \odot \big(\boldsymbol{X}_{\text{in}^0(j)}(\boldsymbol{W}^0_{\text{in}^0(j),j} - \widehat{\boldsymbol{W}}^{ml}_{\text{in}^0(j),j}) + \boldsymbol{Y}_{\text{pa}^0(j)}(\boldsymbol{U}^0_{\text{pa}^0(j),j} - \widehat{\boldsymbol{U}}^{ml}_{\text{pa}^0(j),j}) + \sum_k \alpha^0_{kj}\boldsymbol{h}_k - \sum_k \widehat{\alpha}^{ml}_{kj}\widehat{\boldsymbol{h}}_k\big),
\end{aligned}$$

where $I_1 = \sum_k(\widehat{\alpha}^{ml}_{kj}\widehat{\boldsymbol{h}}_k - \alpha^0_{kj}\boldsymbol{h}_k)$. Further, we have $I_1 = \sum_k(\widehat{\alpha}^{ml}_{kj}\widehat{\boldsymbol{h}}_k - \alpha^0_{kj}\boldsymbol{h}_k) = \sum_k(\widehat{\alpha}^{ml}_{kj}\widehat{\boldsymbol{h}}_k - \alpha^0_{kj}\widehat{\boldsymbol{h}}_k + \alpha^0_{kj}\widehat{\boldsymbol{h}}_k - \alpha^0_{kj}\boldsymbol{h}_k)$. On the other hand, note that $\widetilde{\boldsymbol{Z}}(\boldsymbol{\theta}^0_{A^0_j} - \widehat{\boldsymbol{\theta}}^{ml}_{A^0_j}) = \boldsymbol{X}_{\text{in}^0(j)}(\boldsymbol{W}^0_{\text{in}^0(j),j} - \widehat{\boldsymbol{W}}^{ml}_{\text{in}^0(j),j}) + \boldsymbol{Y}_{\text{pa}^0(j)}(\boldsymbol{U}^0_{\text{pa}^0(j),j} - \widehat{\boldsymbol{U}}^{ml}_{\text{pa}^0(j),j}) + \sum_k(\alpha^0_{kj} - \widehat{\alpha}^{ml}_{kj})\widehat{\boldsymbol{h}}_k$. Therefore, Δ_j can be written as

$$\begin{aligned}
\Delta_j &= \sum_k(\widehat{\alpha}^{ml}_{kj} - \alpha^0_{kj})\widehat{\boldsymbol{h}}_k + \sum_k \alpha^0_{kj}(\widehat{\boldsymbol{h}}_k - \boldsymbol{h}_k) + \varphi'_j(\boldsymbol{\xi}) \odot \widetilde{\boldsymbol{Z}}(\boldsymbol{\theta}^0_{A^0_j} - \widehat{\boldsymbol{\theta}}^{ml}_{A^0_j}) + \varphi'_j(\boldsymbol{\xi}) \odot \sum_k \alpha^0_{kj}(\boldsymbol{h}_k - \widehat{\boldsymbol{h}}_k) \\
&= \sum_k(\widehat{\alpha}^{ml}_{kj} - \alpha^0_{kj})\widehat{\boldsymbol{h}}_k + \varphi'_j(\boldsymbol{\xi}) \odot \widetilde{\boldsymbol{Z}}(\boldsymbol{\theta}^0_{A^0_j} - \widehat{\boldsymbol{\theta}}^{ml}_{A^0_j}) + (1 - \varphi'_j(\boldsymbol{\xi})) \odot \sum_k \alpha^0_{kj}(\widehat{\boldsymbol{h}}_k - \boldsymbol{h}_k).
\end{aligned}$$

Note $\|\sum_k(\widehat{\alpha}^{ml}_{kj} - \alpha^0_{kj})\widehat{\boldsymbol{h}}_k\|_\infty \le \sum_k \|(\widehat{\alpha}^{ml}_{kj} - \alpha^0_{kj})\widehat{\boldsymbol{h}}_k\|_\infty \le \max_k |\widehat{\alpha}^{ml}_{kj} - \alpha^0_{kj}| \cdot \sum_k \|\widehat{\boldsymbol{h}}_k\|_\infty \le \|\boldsymbol{\theta}^0_{A^0_j} - \widehat{\boldsymbol{\theta}}^{ml}_{A^0_j}\|_\infty \cdot \sum_k \|\widehat{\boldsymbol{h}}_k\|_\infty \le b_1 s \cdot \|\boldsymbol{\theta}^0_{A^0_j} - \widehat{\boldsymbol{\theta}}^{ml}_{A^0_j}\|_\infty$, and $\|\sum_k \alpha^0_{kj}(\widehat{\boldsymbol{h}}_k - \boldsymbol{h}_k)\|_\infty \le \sum_k |\alpha^0_{kj}| \cdot \|\Delta_k\|_\infty$. Moreover,

$$\begin{aligned}
\|\widetilde{\boldsymbol{Z}}(\boldsymbol{\theta}^0_{A^0_j} - \widehat{\boldsymbol{\theta}}^{ml}_{A^0_j})\|_\infty &= \|\widetilde{\boldsymbol{Z}}(\widetilde{\boldsymbol{Z}}^\top \boldsymbol{M}\widetilde{\boldsymbol{Z}})^{-1}\widetilde{\boldsymbol{Z}}^\top(\boldsymbol{Y}_j - \varphi_j(\widetilde{\boldsymbol{z}}_i^\top \boldsymbol{\theta}^0_{A^0_j}) - \boldsymbol{r})\|_\infty \\
&= \|\boldsymbol{H}_2(\boldsymbol{Y}_j - \varphi_j(\boldsymbol{z}_i^\top \boldsymbol{\theta}^0_{A^0_j}) + \varphi_j(\boldsymbol{z}_i^\top \boldsymbol{\theta}^0_{A^0_j}) - \varphi_j(\widetilde{\boldsymbol{z}}_i^\top \boldsymbol{\theta}^0_{A^0_j}) - \boldsymbol{r})\|_\infty \\
&\le \|\boldsymbol{H}_2(\boldsymbol{Y}_j - \varphi_j(\boldsymbol{z}_i^\top \boldsymbol{\theta}^0_{A^0_j}))\|_\infty + \|\boldsymbol{H}_2(\varphi_j(\boldsymbol{z}_i^\top \boldsymbol{\theta}^0_{A^0_j}) - \varphi_j(\widetilde{\boldsymbol{z}}_i^\top \boldsymbol{\theta}^0_{A^0_j}))\|_\infty + \|\boldsymbol{H}_2\boldsymbol{r}\|_\infty,
\end{aligned}$$

where $\boldsymbol{H}_2 = \widetilde{\boldsymbol{Z}}(\widetilde{\boldsymbol{Z}}^\top \boldsymbol{M}\widetilde{\boldsymbol{Z}})^{-1}\widetilde{\boldsymbol{Z}}^\top$. Again, by Assumption 5, there exists b_3 such that $\|n\widetilde{\boldsymbol{Z}}(\widetilde{\boldsymbol{Z}}^\top \boldsymbol{M}\widetilde{\boldsymbol{Z}})^{-1}\widetilde{\boldsymbol{Z}}^\top\|_\infty \leq b_3$. Similarly, following the calculation of $\|\widehat{\boldsymbol{\theta}}^{ml}_{A_j^0} - \boldsymbol{\theta}^0_{A_j^0}\|_\infty$, we obtain

$$
\begin{aligned}
||\Delta_j||_\infty &\leq b_1 s \cdot \|\boldsymbol{\theta}^0_{A_j^0} - \widehat{\boldsymbol{\theta}}^{ml}_{A_j^0}\|_\infty + L_1\Bigg[2Mb_3\sqrt{\frac{\log(p(2s+\widetilde{s}))}{n}} + b_3 L_1 \sum_k |\alpha^0_{kj}| \cdot \|\Delta_k\|_\infty \\
&\quad + b_3 c_0 D\left(\frac{32}{m^2}M^2 b_1^2 \cdot \sqrt{\frac{\log(p(2s+\widetilde{s}))}{n}} + \frac{8}{m^2} b_1^2 L_1^2 \sum_k |\alpha^0_{kj}|^2 \cdot \|\Delta_k\|_\infty\right)\Bigg] + (1+L_1)\sum_k |\alpha^0_{kj}| \cdot \|\Delta_k\|_\infty \\
&\leq a_4 \sum_k \|\Delta_k\|_\infty + a_3\sqrt{\frac{\log(p(2s+\widetilde{s}))}{n}} \leq a_4 s \cdot \max_k \|\Delta_k\|_\infty + a_3\sqrt{\frac{\log(p(2s+\widetilde{s}))}{n}},
\end{aligned}
$$

where $a_4 = \max_k \left(b_3 L_1^2 |\alpha^0_{kj}| + \frac{8 b_3 c_0 D b_1^2 L_1^3 |\alpha^0_{kj}|^2}{m^2} + b_1 s\left(b_2 L_1 |\alpha^0_{kj}| + \frac{8 b_2 c_0 D b_1^2 L_1^2 |\alpha^0_{kj}|^2}{m^2}\right) + (1+L_1)|\alpha^0_{kj}|\right)$ and $a_3 = \left(2Mb_3 L_1 + b_3 c_0 D \frac{32}{m^2} M^2 b_1^2 L_1 + b_1 s(2Mb_2 + b_2 c_0 D \frac{32}{m^2} M^2 b_1^2)\right)$. The above inequality can be written as: $\|\Delta_j\|_\infty + c \leq a_4 s\,(\max_k \|\Delta_k\|_\infty + c)$, where $c = \frac{a_3}{a_4 s - 1}\sqrt{\frac{\log(p(2s+\widetilde{s}))}{n}}$. Therefore,

$$
\|\Delta_j\|_\infty + c \leq (a_4 s)^{d_j}(\|\Delta_0\|_\infty + c),
$$

where d_j denotes the topology depth of the primary variable Y_j defined as the maximal length of a directed path in the graph from a root variable with depth zero; therefore, $0 \leq d_j \leq d_{\max} \leq p-1$ with $d_{\max}$ the maximal length of a directed path. Rearranging terms yields

$$
\begin{aligned}
\|\Delta_j\|_\infty &\leq (a_4 s)^{d_j}\|\Delta_0\|_\infty + ((a_4 s)^{d_j} - 1)c \\
&= (a_4 s)^{d_j}\|\Delta_0\|_\infty + ((a_4 s)^{d_j} - 1)\frac{a_3}{a_4 s - 1}\sqrt{\frac{\log(p(2s+\widetilde{s}))}{n}}.
\end{aligned}
$$

In this way, we derive the general form of the accumulated error for $\|\Delta_j\|_\infty$ for the multi-layer case. To conclude, if τ_j satisfies: $\tau_j \geq 2\left\|\widehat{\boldsymbol{\theta}}^{ml} - \boldsymbol{\theta}^0\right\|_\infty = C\sqrt{\frac{\log(p(2s+\widetilde{s}))}{n}}$, then the deconfounding algorithm reconstructs the causal graph consistently, i.e., $\{(k,j) : \widehat{U}_{kj} \neq 0\} = \{(k,j) : U^0_{kj} \neq 0\}$, with probability $1 - 8p \cdot p^{-2}(2s+\widetilde{s})^{-1}$ by the union bound, tending to one as $p \to \infty$ and thus $n \to \infty$. This completes the proof. We next present proofs of the lemmas.

Lemma 9 bounds the quantity $\|\widehat{\boldsymbol{\theta}}^{ml}_{A_j^0} - \boldsymbol{\theta}^0_{A_j^0}\|_2$ in child equations.

Lemma 9 (Rate of convergence under the ℓ_2-norm for child equations)

$$
\begin{aligned}
\|\widehat{\boldsymbol{\theta}}^{ml}_{A_j^0} - \boldsymbol{\theta}^0_{A_j^0}\|_2 &\leq \frac{2}{m}\sqrt{2s+\widetilde{s}} \cdot \|\nabla\mathcal{L}(\boldsymbol{\theta}^0_{A_j^0}|\boldsymbol{Y}_{pa^0(j)}, \boldsymbol{X}_{in^0(j)}, \widehat{\boldsymbol{h}}_{an^0(j)})\|_\infty \\
&\leq \frac{2}{m}\sqrt{2s+\widetilde{s}}\left[2Mb_1\sqrt{\frac{\log(p(2s+\widetilde{s}))}{n}} + b_1 L_1 \sum_k |\alpha^0_{kj}| \cdot \|\Delta_k\|_\infty\right],
\end{aligned}
$$

with probability at least $1 - 2\exp(-2\log p - \log(2s+\widetilde{s})) = 1 - 2p^{-2}(2s+\widetilde{s})^{-1}$.

Proof of Lemma 9. Since $\widehat{\boldsymbol{\theta}}^{ml}_{A_j^0} = (\widehat{\boldsymbol{W}}^{ml}_{\text{in}^0(j),j}, \widehat{\boldsymbol{U}}^{ml}_{\text{pa}^0(j),j}, \widehat{\boldsymbol{\alpha}}^{ml}_{\text{an}^0(j),j})$ minimizes $\mathcal{L}(\boldsymbol{\theta}_{A_j^0}|\boldsymbol{Y}_{\text{pa}^0(j)}, \boldsymbol{X}_{\text{in}^0(j)}, \widehat{\boldsymbol{h}}_{\text{an}^0(j)})$, $\mathcal{L}(\widehat{\boldsymbol{\theta}}^{ml}_{A_j^0}|\boldsymbol{Y}_{\text{pa}^0(j)}, \boldsymbol{X}_{\text{in}^0(j)}, \widehat{\boldsymbol{h}}_{\text{an}^0(j)}) \leq \mathcal{L}(\boldsymbol{\theta}^0_{A_j^0}|\boldsymbol{Y}_{\text{pa}^0(j)}, \boldsymbol{X}_{\text{in}^0(j)}, \widehat{\boldsymbol{h}}_{\text{an}^0(j)})$.

As in Lemma 8, $\|\widehat{\boldsymbol{\theta}}^{ml}_{A^0_j} - \boldsymbol{\theta}^0_{A^0_j}\|_2 \leq \frac{2}{m}\sqrt{2s+\widetilde{s}} \cdot \|\nabla \mathcal{L}(\boldsymbol{\theta}^0_{A^0_j}|\boldsymbol{Y}_{\mathrm{pa}^0(j)}, \boldsymbol{X}_{\mathrm{in}^0(j)}, \widehat{\boldsymbol{h}}_{\mathrm{an}^0(j)})\|_\infty$. Meanwhile,

$$
\begin{aligned}
&\|\nabla \mathcal{L}(\boldsymbol{\theta}^0_{A^0_j}|\boldsymbol{Y}_{\mathrm{pa}^0(j)}, \boldsymbol{X}_{\mathrm{in}^0(j)}, \widehat{\boldsymbol{h}}_{\mathrm{an}^0(j)})\|_\infty \\
&= n^{-1}\|\widetilde{\boldsymbol{Z}}^\top(\boldsymbol{Y}_j - \varphi_j(\boldsymbol{X}_{\mathrm{in}^0(j)}\boldsymbol{W}^0_{\mathrm{in}^0(j),j} + \boldsymbol{Y}_{\mathrm{pa}^0(j)}\boldsymbol{U}^0_{\mathrm{pa}^0(j),j} + \sum_k \alpha^0_{kj}\widehat{\boldsymbol{h}}_k))\|_\infty \\
&= n^{-1}\big\|T_1 + T_2\big\|_\infty \leq n^{-1}\big\|T_1\|_\infty + n^{-1}\big\|T_2\|_\infty \\
&\leq n^{-1}\|T_1\|_\infty + n^{-1}\big\|\widetilde{\boldsymbol{Z}}^\top \cdot \big(\sum_k \alpha^0_{kj}(\boldsymbol{h}_k - \widehat{\boldsymbol{h}}_k) \odot \varphi'_j(\boldsymbol{\xi})\big)\big\|_\infty \\
&\leq n^{-1}\|T_1\|_\infty + n^{-1}\big\|\widetilde{\boldsymbol{Z}}^\top \cdot \big(\sum_k \alpha^0_{kj}\Big(\Delta_k + (\boldsymbol{Y}_k - \mathbb{E}[\boldsymbol{Y}_k|\boldsymbol{X}_{\mathrm{in}^0(k)}, \boldsymbol{h}_k])\Big) \odot \varphi'_j(\boldsymbol{\xi})\big)\big\|_\infty \qquad \text{by (43)} \\
&\leq n^{-1}\|T_1\|_\infty + b_1 L_1 \sum_k |\alpha^0_{kj}| \cdot \|\Delta_k\|_\infty + L_1 n^{-1}\big\|\widetilde{\boldsymbol{Z}}^\top\big(\sum_k \alpha^0_{kj}(\boldsymbol{Y}_k - \mathbb{E}[\boldsymbol{Y}_k|\boldsymbol{X}_{\mathrm{in}^0(k)}, \boldsymbol{h}_k])\big)\big\|_\infty,
\end{aligned}
$$

where $T_1 = \widetilde{\boldsymbol{Z}}^\top(\boldsymbol{Y}_j - \varphi_j(\boldsymbol{X}_{\mathrm{in}^0(j)}\boldsymbol{W}^0_{\mathrm{in}^0(j),j} + \boldsymbol{Y}_{\mathrm{pa}^0(j)}\boldsymbol{U}^0_{\mathrm{pa}^0(j),j} + \sum_k \alpha^0_{kj}\boldsymbol{h}_k))$ and $T_2 = \widetilde{\boldsymbol{Z}}^\top(\varphi_j(\boldsymbol{X}_{\mathrm{in}^0(j)}\boldsymbol{W}^0_{\mathrm{in}^0(j),j} + \boldsymbol{Y}_{\mathrm{pa}^0(j)}\boldsymbol{U}^0_{\mathrm{pa}^0(j),j} + \sum_k \alpha^0_{kj}\boldsymbol{h}_k) - \varphi_j(\boldsymbol{X}_{\mathrm{in}^0(j)}\boldsymbol{W}^0_{\mathrm{in}^0(j),j} + \boldsymbol{Y}_{\mathrm{pa}^0(j)}\boldsymbol{U}^0_{\mathrm{pa}^0(j),j} + \sum_k \alpha^0_{kj}\widehat{\boldsymbol{h}}_k))$. The last inequality holds as $|\varphi'_j(z)| = |A''_j(z)| \leq L_1$.

For the last term above, $n^{-1}\|\widetilde{\boldsymbol{Z}}^\top\big(\sum_k \alpha^0_{kj}(\boldsymbol{Y}_k - \mathbb{E}[\boldsymbol{Y}_k|\boldsymbol{X}_{\mathrm{in}^0(k)}, \boldsymbol{h}_k])\big)\|_\infty = n^{-1}\|\sum_k \alpha^0_{kj}\widetilde{\boldsymbol{Z}}^\top(\boldsymbol{Y}_k - \mathbb{E}[\boldsymbol{Y}_k|\boldsymbol{X}_{\mathrm{in}^0(k)}, \boldsymbol{h}_k])\|_\infty$. Similarly, by Assumption 3 and the Bernstein's inequality, we conclude that $n^{-1}\|\sum_k \alpha^0_{kj}\widetilde{\boldsymbol{Z}}^\top(\boldsymbol{Y}_k - \mathbb{E}[\boldsymbol{Y}_k|\boldsymbol{X}_{\mathrm{in}^0(k)}, \boldsymbol{h}_k])\|_\infty = o(\sqrt{\frac{\log(p(2s+\widetilde{s}))}{n}})$ with probability tending to 1.

Note that $\|\widetilde{\boldsymbol{Z}}\|_\infty \leq b_1$ and $\widetilde{\boldsymbol{Z}} = [\boldsymbol{X}_{\mathrm{in}^0(j)}, \boldsymbol{Y}_{\mathrm{pa}^0(j)}, \widehat{\boldsymbol{h}}_{\mathrm{an}^0(j)}] \in \mathbb{R}^{2s+\widetilde{s}}$. The first term is also bounded by the Bernstein's inequality since $\mathbb{E}[\boldsymbol{Y}_j|\boldsymbol{Z}] = \varphi_j(\boldsymbol{X}_{\mathrm{in}^0(j)}\boldsymbol{W}^0_{\mathrm{in}^0(j),j} + \boldsymbol{Y}_{\mathrm{pa}^0(j)}\boldsymbol{U}^0_{\mathrm{pa}^0(j),j} + \sum_k \alpha^0_{kj}\boldsymbol{h}_k)$.

$$
\mathbb{P}(n^{-1}\|T_1\|_\infty > \epsilon) \leq 2(2s+\widetilde{s})\exp\left(-\min\left(\frac{n\epsilon^2}{2M^2 b_1^2}, \frac{n\epsilon}{2Mb_1}\right)\right).
$$

Setting $\epsilon = \eta_1\sqrt{\frac{\log(p(2s+\widetilde{s}))}{n}}$ yields

$$
\mathbb{P}\left(n^{-1}\|T_1\|_\infty \leq \eta_1\sqrt{\frac{\log(p(2s+\widetilde{s}))}{n}}\right) \geq 1 - 2\exp\left(-\frac{1}{2}(\frac{\eta_1^2}{M^2 b_1^2} - 2)\log(2s+\widetilde{s}) - \frac{\eta_1^2}{2M^2 b_1^2}\log p\right).
$$

In particular, setting $\eta_1 = 2Mb_1$ yields

$$
\|\widehat{\boldsymbol{\theta}}^{ml}_{A^0_j} - \boldsymbol{\theta}^0_{A^0_j}\|_2 \leq \frac{2}{m}\sqrt{2s+\widetilde{s}}\left[2Mb_1\sqrt{\frac{\log(p(2s+\widetilde{s}))}{n}} + b_1 L_1 \sum_k |\alpha^0_{kj}| \cdot \|\Delta_k\|_\infty\right],
$$

with probability at least $1 - 2\exp(-2\log p - \log(2s+\widetilde{s})) = 1 - 2p^{-2}(2s+\widetilde{s})^{-1}$.

Lemma 10 bounds the quantity $\|\widehat{\boldsymbol{W}}^{ml}_{\mathrm{in}^0(k),k} - \boldsymbol{W}^0_{\mathrm{in}^0(k),k}\|_2$ in root equations.

Lemma 10 (Rate of convergence under the ℓ_2-norm for root equations)

$$
\|\widehat{\boldsymbol{W}}^{ml}_{in^0(k),k} - \boldsymbol{W}^0_{in^0(k),k}\|_2 \leq \frac{2}{m}\sqrt{\widetilde{s}}\left[2Mb_1\sqrt{\frac{\log(p\widetilde{s})}{n}}\right],
$$

with probability at least $1 - 2\exp(-2\log p - \log\widetilde{s}) = 1 - 2p^{-2}\widetilde{s}^{-1}$.

Proof of Lemma 10. Consider the log-likelihood for a root variable Y_k:

$$\mathcal{L}(\boldsymbol{W}_{\text{in}^0(k),k}|\boldsymbol{Y}_k, \boldsymbol{X}_{\text{in}^0(k)}) = n^{-1}\sum_{i=1}^{n} -Y_{ik}\left(\boldsymbol{W}_{\text{in}^0(k),k}^{\top}\boldsymbol{X}_{i,\text{in}^0(k)}\right) + A_k\left(\boldsymbol{W}_{\text{in}^0(k),k}^{\top}\boldsymbol{X}_{i,\text{in}^0(k)}\right),$$

where the oracle estimator $\widehat{\boldsymbol{W}}_{\text{in}^0(k),k}^{ml}$ is its minimizer with respect to $\boldsymbol{W}_{\text{in}^0(k),k}$.

By the definition of $\widehat{\boldsymbol{W}}_{\text{in}^0(k),k}^{ml}$, it follows from Lemma 8 that $\|\widehat{\boldsymbol{W}}_{\text{in}^0(k),k}^{ml} - \boldsymbol{W}_{\text{in}^0(k),k}^{0}\|_2 \le \frac{2}{m}\sqrt{\widetilde{s}} \cdot \|\nabla\mathcal{L}(\boldsymbol{W}_{\text{in}^0(k),k}^{0}|\boldsymbol{Y}_k, \boldsymbol{X}_{\text{in}^0(k)})\|_\infty$, where $\nabla\mathcal{L}(\boldsymbol{W}_{\text{in}^0(k),k}^{0}|\boldsymbol{Y}_k, \boldsymbol{X}_{\text{in}^0(k)})$ is the gradient of $\mathcal{L}(\boldsymbol{W}_{\text{in}^0(k),k}^{0}|\boldsymbol{Y}_k, \boldsymbol{X}_{\text{in}^0(k)})$. By the triangular inequality,

$$\begin{aligned}
&\|\nabla\mathcal{L}(\boldsymbol{W}_{\text{in}^0(k),k}^{0}|\boldsymbol{Y}_k, \boldsymbol{X}_{\text{in}^0(k)})\|_\infty = n^{-1}\|\boldsymbol{X}_{\text{in}^0(k)}^{\top}(\boldsymbol{Y}_k - \varphi_k(\boldsymbol{X}_{\text{in}^0(k)}\boldsymbol{W}_{\text{in}^0(k),k}^{0}))\|_\infty \\
&\le n^{-1}\|\boldsymbol{X}_{\text{in}^0(k)}^{\top}(\boldsymbol{Y}_k - \varphi_k(\boldsymbol{X}_{\text{in}^0(k)}\boldsymbol{W}_{\text{in}^0(k),k}^{0} + \boldsymbol{h}_k))\|_\infty \\
&\quad + n^{-1}\|\boldsymbol{X}_{\text{in}^0(k)}^{\top}(\varphi_k(\boldsymbol{X}_{\text{in}^0(k)}\boldsymbol{W}_{\text{in}^0(k),k}^{0} + \boldsymbol{h}_k) - \varphi_k(\boldsymbol{X}_{\text{in}^0(k)}\boldsymbol{W}_{\text{in}^0(k),k}^{0}))\|_\infty \\
&\equiv G_1 + G_2.
\end{aligned} \tag{42}$$

Note that $\|\boldsymbol{X}_{\text{in}^0(k)}\|_\infty \le b_1$ and $\mathbb{E}[\boldsymbol{Y}_k|\boldsymbol{X}_{\text{in}^0(k)}, \boldsymbol{h}_k] = \varphi_k(\boldsymbol{X}_{\text{in}^0(k)}\boldsymbol{W}_{\text{in}^0(k),k}^{0} + \boldsymbol{h}_k)$. By the Bernstein's inequality, the first term in (42) is bounded by

$$\mathbb{P}(G_1 > \epsilon) \le 2\widetilde{s}\exp\left(-\min\left(\frac{n\epsilon^2}{2M^2b_1^2}, \frac{n\epsilon}{2Mb_1}\right)\right).$$

Setting $\epsilon = 2Mb_1\sqrt{\frac{\log(p\widetilde{s})}{n}}$ leads to $G_1 \le 2Mb_1\sqrt{\frac{\log(p\widetilde{s})}{n}}$ with probability at least $1 - 2\exp(-2\log p - \log\widetilde{s}) = 1 - 2p^{-2}\widetilde{s}^{-1}$.

For G_2 in (42), by the Taylor series expansion,

$$\begin{aligned}
G_2 &= n^{-1}\|\boldsymbol{X}_{\text{in}^0(k)}^{\top}\cdot\left(\varphi_k'(\boldsymbol{X}_{\text{in}^0(k)}\boldsymbol{W}_{\text{in}^0(k),k}^{0}) \odot \boldsymbol{h}_k\right)\|_\infty \\
&= n^{-1}\|(\boldsymbol{X}_{\text{in}^0(k)}^{\top}\text{diag}(\varphi_k'(\boldsymbol{X}_{\text{in}^0(k)}\boldsymbol{W}_{\text{in}^0(k),k}^{0}))) \cdot \boldsymbol{h}_k\|_\infty.
\end{aligned}$$

Note that $\boldsymbol{X}_{\text{in}^0(k)}$ and $\boldsymbol{h}_k$ are independent. Hence, $\mathbb{E}[\left(\boldsymbol{X}_{\text{in}^0(k)}^{\top}\text{diag}(\varphi_k'(\boldsymbol{X}_{\text{in}^0(k)}\boldsymbol{W}_{\text{in}^0(k),k}^{0}))\right) \cdot \boldsymbol{h}_k] = 0$. By the bounded domain for interventions condition, there exists b_4 such that $\|\boldsymbol{X}_{\text{in}^0(k)}^{\top}\text{diag}(\varphi_k'(\boldsymbol{X}_{\text{in}^0(k)}\boldsymbol{W}_{\text{in}^0(k),k}^{0}))\|_\infty \le b_4$. For $j \in \text{in}^0(k)$, by the Hoeffding's inequality,

$$\mathbb{P}(n^{-1}\|\boldsymbol{X}_j^{\top}\text{diag}(\varphi_k'(\boldsymbol{X}_{\text{in}^0(k)}\boldsymbol{W}_{\text{in}^0(k),k}^{0}))\boldsymbol{h}_k\|_\infty > \epsilon) \le 2\exp\left(-\frac{n\epsilon^2}{2\sigma_j^2b_4^2}\right).$$

Applying the union bound and setting $\epsilon = 2\sigma_j b_4\sqrt{\frac{\log(p\widetilde{s})}{n}}$ yield $G_2 \le 2\sigma_j b_4\sqrt{\frac{\log(p\widetilde{s})}{n}}$ with probability at least $1 - 2\exp(-2\log p - \log\widetilde{s}) = 1 - 2p^{-2}\widetilde{s}^{-1}$. For simplicity, set $G_2 = o(\sqrt{\frac{\log(p\widetilde{s})}{n}})$.

Finally, combining the two terms in (42) yields:

$$\|\widehat{\boldsymbol{W}}_{\text{in}^0(k),k}^{ml} - \boldsymbol{W}_{\text{in}^0(k),k}^{0}\|_2 \le \frac{2}{m}\sqrt{\widetilde{s}}\cdot\|\nabla\mathcal{L}(\boldsymbol{W}_{\text{in}^0(k),k}^{0}|\boldsymbol{Y}_k, \boldsymbol{X}_{\text{in}^0(k)})\|_\infty \le \frac{2}{m}\sqrt{\widetilde{s}}\big(2Mb_1\sqrt{\frac{\log(p\widetilde{s})}{n}}\big).$$

Lemma 11 derives the estimation bound for $\|\widehat{\boldsymbol{W}}_{\overline{\text{in}}(k),k} - \boldsymbol{W}_{\text{in}^0(k),k}^{0}\|_\infty$ in root equations.

Lemma 11 (Rate of convergence under the ℓ_∞-norm for root equations)

$$\|\widehat{\boldsymbol{W}}_{\overline{in}(k),k} - \boldsymbol{W}^0_{in^0(k),k}\|_\infty \le \left(2Mb_2 + b_2c_0D\frac{16}{m^2}M^2b_1^2\right)\sqrt{\frac{\log(p\widetilde{s})}{n}},$$

with probability at least $1-4\exp(-2\log p - \log\widetilde{s}) = 1-4p^{-2}\widetilde{s}^{-1}$. *Further, the estimation error of the confounder* $\boldsymbol{h}_k$ *satisfies:* $||\Delta_k||_\infty \le \left(2Mb_3 + b_3c_0D\frac{16}{m^2}M^2b_1^2\right)\sqrt{\frac{\log(p\widetilde{s})}{n}}$.

Proof of Lemma 11. Note that by Theorem 4, $\{l : \widehat{V}_{lk} \neq 0\} = \{l : V^0_{lk} \neq 0\}$, implying that $\overline{\mathrm{in}}(k) = \mathrm{in}^0(k)$ in root equations. Therefore, by construction, $\widehat{\boldsymbol{W}}_{\overline{\mathrm{in}}(k),k} = \widehat{\boldsymbol{W}}^{ml}_{\mathrm{in}^0(k),k}$, as both are GLM estimators constrained on the same set. It suffices to derive the error bound for the oracle estimator.

To establish the ℓ_∞-norm of the oracle estimator, as in Lemma 7, we apply the Taylor series expansion of $\varphi_k(\boldsymbol{X}_{\mathrm{in}^0(k)}\boldsymbol{W}_{\mathrm{in}^0(k),k})$ as:

$$\boldsymbol{X}^\top_{\mathrm{in}^0(k)}\boldsymbol{M}\boldsymbol{X}_{\mathrm{in}^0(k)}(\widehat{\boldsymbol{W}}^{ml}_{\mathrm{in}^0(k),k} - \boldsymbol{W}^0_{\mathrm{in}^0(k),k}) = \boldsymbol{X}^\top_{\mathrm{in}^0(k)}(\boldsymbol{Y}_k - \varphi_k(\boldsymbol{X}_{\mathrm{in}^0(k)}\boldsymbol{W}^0_{\mathrm{in}^0(k),k}) - \boldsymbol{r}).$$

This implies that $\widehat{\boldsymbol{W}}^{ml}_{\mathrm{in}^0(k),k} - \boldsymbol{W}^0_{\mathrm{in}^0(k),k} = \boldsymbol{H}(\boldsymbol{Y}_k - \varphi_k(\boldsymbol{X}_{\mathrm{in}^0(k)}\boldsymbol{W}^0_{\mathrm{in}^0(k),k}) - \boldsymbol{r})$, where $\boldsymbol{H} = (\boldsymbol{X}^\top_{\mathrm{in}^0(k)}\boldsymbol{M}\boldsymbol{X}_{\mathrm{in}^0(k)})^{-1}\boldsymbol{X}^\top_{\mathrm{in}^0(k)}$. Then,

$$\begin{aligned}
&\|\widehat{\boldsymbol{W}}^{ml}_{\mathrm{in}^0(k),k} - \boldsymbol{W}^0_{\mathrm{in}^0(k),k}\|_\infty = \|\boldsymbol{H}(\boldsymbol{Y}_k - \varphi_k(\boldsymbol{X}_{\mathrm{in}^0(k)}\boldsymbol{W}^0_{\mathrm{in}^0(k),k} + \boldsymbol{h}_k) \\
&\quad + \varphi_k(\boldsymbol{X}_{\mathrm{in}^0(k)}\boldsymbol{W}^0_{\mathrm{in}^0(k),k} + \boldsymbol{h}_k) - \varphi_k(\boldsymbol{X}_{\mathrm{in}^0(k)}\boldsymbol{W}^0_{\mathrm{in}^0(k),k}) - \boldsymbol{r})\|_\infty \\
&\le \|\boldsymbol{H}(\boldsymbol{Y}_k - \varphi_k(\boldsymbol{X}_{\mathrm{in}^0(k)}\boldsymbol{W}^0_{\mathrm{in}^0(k),k} + \boldsymbol{h}_k))\|_\infty \\
&\quad + \|\boldsymbol{H}(\varphi_k(\boldsymbol{X}_{\mathrm{in}^0(k)}\boldsymbol{W}^0_{\mathrm{in}^0(k),k} + \boldsymbol{h}_k) - \varphi_k(\boldsymbol{X}_{\mathrm{in}^0(k)}\boldsymbol{W}^0_{\mathrm{in}^0(k),k}))\|_\infty + \|\boldsymbol{H}\boldsymbol{r}\|_\infty, \quad \equiv I_1 + I_2 + I_3.
\end{aligned}$$

By Assumption 5, there exists b_2 such that $\|n(\boldsymbol{X}^\top_{\mathrm{in}^0(k)}\boldsymbol{M}\boldsymbol{X}_{\mathrm{in}^0(k)})^{-1}\boldsymbol{X}^\top_{\mathrm{in}^0(k)}\|_\infty \le b_2$. Note that $\mathbb{E}[\boldsymbol{Y}_k|\boldsymbol{X}_{\mathrm{in}^0(k)}, \boldsymbol{h}_k] = \varphi_k(\boldsymbol{X}_{\mathrm{in}^0(k)}\boldsymbol{W}^0_{\mathrm{in}^0(k),k} + \boldsymbol{h}_k)$. Then, by the Bernstein's inequality:

$$\mathbb{P}(I_1 > \epsilon) \le 2\widetilde{s}\exp\left(-\min\left(\frac{n\epsilon^2}{2M^2b_2^2}, \frac{n\epsilon}{2Mb_2}\right)\right).$$

Setting $\epsilon = 2Mb_2\sqrt{\frac{\log(p\widetilde{s})}{n}}$ yields $I_1 \le 2Mb_2\sqrt{\frac{\log(p\widetilde{s})}{n}}$ with probability at least $1-2\exp(-2\log p - \log\widetilde{s}) = 1-2p^{-2}\widetilde{s}^{-1}$. On the other hand, as in Lemma 10, we have

$$\begin{aligned}
I_2 &= \|\boldsymbol{H}\left(\boldsymbol{h}_k \odot \varphi'_k(\boldsymbol{X}_{\mathrm{in}^0(k)}\boldsymbol{W}^0_{\mathrm{in}^0(k),k})\right)\|_\infty \\
&= \|(\boldsymbol{X}^\top_{\mathrm{in}^0(k)}\boldsymbol{M}\boldsymbol{X}_{\mathrm{in}^0(k)})^{-1}\boldsymbol{X}^\top_{\mathrm{in}^0(k)} \cdot \left(\boldsymbol{h}_k \odot \varphi'_k(\boldsymbol{X}_{\mathrm{in}^0(k)}\boldsymbol{W}^0_{\mathrm{in}^0(k),k})\right)\|_\infty = o(\sqrt{\frac{\log(p\widetilde{s})}{n}}).
\end{aligned}$$

Finally, as in Theorem 4 and Lemma 7,

$$\begin{aligned}
|\boldsymbol{H}_{k\bullet}\boldsymbol{r}| &= |\sum_{i=1}^n H_{ki}r_i| \le \sum_{i=1}^n |H_{ki}||r_i| \le b_2\sum_{i=1}^n |r_i|/n \\
&\le b_2D(\widehat{\boldsymbol{W}}^{ml}_{\mathrm{in}^0(k),k} - \boldsymbol{W}^0_{\mathrm{in}^0(k),k})^\top(\boldsymbol{X}^\top_{\mathrm{in}^0(k)}\boldsymbol{X}_{\mathrm{in}^0(k)}/n)(\widehat{\boldsymbol{W}}^{ml}_{\mathrm{in}^0(k),k} - \boldsymbol{W}^0_{\mathrm{in}^0(k),k}) \\
&\le b_2c_0D\|\widehat{\boldsymbol{W}}^{ml}_{\mathrm{in}^0(k),k} - \boldsymbol{W}^0_{\mathrm{in}^0(k),k}\|_2^2.
\end{aligned}$$

By Lemma 10, $\|\widehat{\boldsymbol{W}}^{ml}_{\mathrm{in}^0(k),k} - \boldsymbol{W}^0_{\mathrm{in}^0(k),k}\|_2 \le \frac{2}{m}\sqrt{\widetilde{s}}\left[2Mb_1\sqrt{\frac{\log(p\widetilde{s})}{n}}\right]$. Therefore,

$$I_3 = \max_k |\boldsymbol{H}_{k\bullet}\boldsymbol{r}| \le b_2c_0D\left(\frac{2}{m}\sqrt{\widetilde{s}}\left[2Mb_1\sqrt{\frac{\log(p\widetilde{s})}{n}}\right]\right)^2 \le b_2c_0D\frac{16}{m^2}M^2b_1^2\sqrt{\frac{\log(p\widetilde{s})}{n}},$$

where the last inequality holds as $n > \widetilde{s}^2\log(p\widetilde{s})$. Therefore, combining I_1, I_2 and I_3 yields

$$\|\widehat{\boldsymbol{W}}^{ml}_{\mathrm{in}^0(k),k} - \boldsymbol{W}^0_{\mathrm{in}^0(k),k}\|_\infty \le a_1\sqrt{\frac{\log(p\widetilde{s})}{n}},$$

with probability greater than $1 - 4\exp(-2\log p - \log\widetilde{s}) = 1 - 4p^{-2}\widetilde{s}^{-1}$. Here, $a_1 = 2Mb_2 + b_2c_0D\frac{16}{m^2}M^2b_1^2$. Lastly, recall that $\overline{\mathrm{in}}(k) = \mathrm{in}^0(k)$ and $\widehat{\boldsymbol{W}}_{\overline{\mathrm{in}}(k),k} = \widehat{\boldsymbol{W}}^{ml}_{\mathrm{in}^0(k),k}$. Therefore, $\|\widehat{\boldsymbol{W}}_{\overline{\mathrm{in}}(k),k} - \boldsymbol{W}^0_{\mathrm{in}^0(k),k}\|_\infty \le a_1\sqrt{\frac{\log(p\widetilde{s})}{n}}$.

To compute the estimation error of the confounder $\boldsymbol{h}_k$, note that, by construction,

$$\widehat{\boldsymbol{h}}_k = \boldsymbol{Y}_k - \varphi_k(\boldsymbol{X}_{\overline{\mathrm{in}}(k)}\widehat{\boldsymbol{W}}_{\overline{\mathrm{in}}(k),k}) = \boldsymbol{Y}_k - \varphi_k(\boldsymbol{X}_{\mathrm{in}^0(k)}\widehat{\boldsymbol{W}}^{ml}_{\mathrm{in}^0(k),k}).$$

On the other hand, by (2) and following (A5)-(A7) in Appendix A of Johnston et al. (2008),

$$\mathbb{E}[\boldsymbol{Y}_k|\boldsymbol{X}_{\mathrm{in}^0(k)},\boldsymbol{h}_k] = \varphi_k(\boldsymbol{X}_{\mathrm{in}^0(k)}\boldsymbol{W}^0_{\mathrm{in}^0(k),k} + \boldsymbol{h}_k) = \varphi_k(\boldsymbol{X}_{\mathrm{in}^0(k)}\boldsymbol{W}^0_{\mathrm{in}^0(k),k}) + \varphi_k'(\boldsymbol{\xi})\odot\boldsymbol{h}_k.$$

Rearranging terms yields $\boldsymbol{h}_k = \left[\mathbb{E}[\boldsymbol{Y}_k|\boldsymbol{X}_{\mathrm{in}^0(k)},\boldsymbol{h}_k] - \varphi_k(\boldsymbol{X}_{\mathrm{in}^0(k)}\boldsymbol{W}^0_{\mathrm{in}^0(k),k})\right]\odot(\varphi_k'(\boldsymbol{\xi}))^{-1}$. We now use $\mathbb{E}[\boldsymbol{Y}_k|\boldsymbol{X}_{\mathrm{in}^0(k)},\boldsymbol{h}_k] - \varphi_k(\boldsymbol{X}_{\mathrm{in}^0(k)}\boldsymbol{W}^0_{\mathrm{in}^0(k),k})$ to approximate $\boldsymbol{h}_k$ as we estimate the coefficient of the confounder in subsequent child equations. This reparametrization and approximations permits a comparison of $\widehat{\boldsymbol{h}}_k$ and $\boldsymbol{h}_k$ at the same scale; see Appendix A of Johnston et al. (2008) for some details about such approximations. Hence,

$$\begin{aligned}
\widehat{\boldsymbol{h}}_k - \boldsymbol{h}_k &= -\varphi_k(\boldsymbol{X}_{\mathrm{in}^0(k)}\widehat{\boldsymbol{W}}^{ml}_{\mathrm{in}^0(k),k}) + \varphi_k(\boldsymbol{X}_{\mathrm{in}^0(k)}\boldsymbol{W}^0_{\mathrm{in}^0(k),k}) + (\boldsymbol{Y}_k - \mathbb{E}[\boldsymbol{Y}_k|\boldsymbol{X}_{\mathrm{in}^0(k)},\boldsymbol{h}_k])\\
&= -\varphi_k'(\boldsymbol{\xi})\odot\boldsymbol{X}_{\mathrm{in}^0(k)}(\widehat{\boldsymbol{W}}^{ml}_{\mathrm{in}^0(k),k} - \boldsymbol{W}^0_{\mathrm{in}^0(k),k}) + (\boldsymbol{Y}_k - \mathbb{E}[\boldsymbol{Y}_k|\boldsymbol{X}_{\mathrm{in}^0(k)},\boldsymbol{h}_k])\\
&= -\varphi_k'(\boldsymbol{\xi})\odot\boldsymbol{H}_2(\boldsymbol{Y}_k - \varphi_k(\boldsymbol{X}_{\mathrm{in}^0(k)}\boldsymbol{W}^0_{\mathrm{in}^0(k),k}) - \boldsymbol{r}) + (\boldsymbol{Y}_k - \mathbb{E}[\boldsymbol{Y}_k|\boldsymbol{X}_{\mathrm{in}^0(k)},\boldsymbol{h}_k])\\
&= \Delta_k + (\boldsymbol{Y}_k - \mathbb{E}[\boldsymbol{Y}_k|\boldsymbol{X}_{\mathrm{in}^0(k)},\boldsymbol{h}_k]),
\end{aligned} \tag{43}$$

where $\boldsymbol{H}_2 = \boldsymbol{X}_{\mathrm{in}^0(k)}(\boldsymbol{X}^\top_{\mathrm{in}^0(k)}\boldsymbol{M}\boldsymbol{X}_{\mathrm{in}^0(k)})^{-1}\boldsymbol{X}^\top_{\mathrm{in}^0(k)}$ and $\Delta_k = -\varphi_k'(\boldsymbol{\xi})\odot\boldsymbol{H}_2(\boldsymbol{Y}_k - \varphi_k(\boldsymbol{X}_{\mathrm{in}^0(k)}\boldsymbol{W}^0_{\mathrm{in}^0(k),k}) - \boldsymbol{r})$. Hence, the estimation error of the confounder consists of two terms: the prediction error Δ_k and an approximation error. For the approximation error, by Assumption 3, Y_{ik} is sub-exponential and $Y_{ik} - \mathbb{E}[Y_{ik}|\cdot]$ is sub-exponential with mean zero. Therefore, we have $\boldsymbol{\epsilon} = \boldsymbol{Y}_k - \mathbb{E}[\boldsymbol{Y}_k|\boldsymbol{X}_{\mathrm{in}^0(k)},\boldsymbol{h}_k]$, where ϵ_i is sub-exponential with mean zero. We show in the proof of Theorem 5 and Lemma 9 that the aggregate impact of this term across all samples is of the order of $o(\sqrt{\frac{\log(p(2s+\widetilde{s}))}{n}})$ when calculating the estimation error of parameters in the subsequent child equations. For the prediction error Δ_k, by the triangular inequality,

$$\begin{aligned}
\|\Delta_k\|_\infty &\le L_1\cdot\|\boldsymbol{H}_2(\boldsymbol{Y}_k - \varphi_k(\boldsymbol{X}_{\mathrm{in}^0(k)}\boldsymbol{W}^0_{\mathrm{in}^0(k),k}) - \boldsymbol{r})\|_\infty\\
&\le L_1\cdot\big(\|\boldsymbol{H}_2(\boldsymbol{Y}_k - \varphi_k(\boldsymbol{X}_{\mathrm{in}^0(k)}\boldsymbol{W}^0_{\mathrm{in}^0(k),k} + \boldsymbol{h}_k))\|_\infty\\
&\quad + \|\boldsymbol{H}_2(\varphi_k(\boldsymbol{X}_{\mathrm{in}^0(k)}\boldsymbol{W}^0_{\mathrm{in}^0(k),k} + \boldsymbol{h}_k) - \varphi_k(\boldsymbol{X}_{\mathrm{in}^0(k)}\boldsymbol{W}^0_{\mathrm{in}^0(k),k}))\|_\infty + \|\boldsymbol{H}_2\boldsymbol{r}\|_\infty\big).
\end{aligned}$$

By the bounded domain for interventions condition, there exists b_3 such that $\|n\boldsymbol{X}_{\mathrm{in}^0(k)}(\boldsymbol{X}_{\mathrm{in}^0(k)}^\top \boldsymbol{M}\boldsymbol{X}_{\mathrm{in}^0(k)})^{-1}\boldsymbol{X}_{\mathrm{in}^0(k)}^\top\|_\infty \leq b_3$. Similarly, $||\Delta_k||_\infty \leq a_2 L_1\sqrt{\frac{\log(p\widetilde{s})}{n}}$, with probability greater than $1-4\exp(-2\log p-\log\widetilde{s}) = 1-4p^{-2}\widetilde{s}^{-1}$. Here, $a_2 = 2Mb_3+b_3c_0D\frac{16}{m^2}M^2b_1^2$. This completes the proof.
